# Intestinal long non-coding RNAs in response to simulated microgravity stress in *Caenorhabditis elegans*

**DOI:** 10.1038/s41598-021-81619-4

**Published:** 2021-01-21

**Authors:** Lingmei Sun, Dan Li, Yujie Yuan, Dayong Wang

**Affiliations:** Medical School, Southeast University, Nanjing, 210009 China

**Keywords:** Environmental impact, Biomarkers

## Abstract

Long non-coding RNAs (lncRNAs) are important in regulating the response to environmental stresses in organisms. In this study, we used *Caenorhabditis elegans* as an animal model to determine the functions of intestinal lncRNAs in regulating response to simulated microgravity stress. Among the intestinal lncRNAs, *linc-2*, *linc-46*, *linc-61*, and *linc-78* were increased by simulated microgravity treatment, and *linc-13*, *linc-14*, *linc-50*, and *linc-125* were decreased by simulated microgravity treatment. Among these 8 intestinal lncRNAs, RNAi knockdown of *linc-2* or *linc-61* induced a susceptibility to toxicity of simulated microgravity, whereas RNAi knockdown of *linc-13*, *linc-14*, or *linc-50* induced a resistance to toxicity of simulated microgravity. In simulated microgravity treated nematodes, *linc-50* potentially binds to three transcriptional factors (DAF-16, SKN-1, and HLH-30). RNAi knockdown of *daf-16*, *skn-1*, or *hlh-30* could suppress resistance of *linc-50(RNAi)* nematodes to the toxicity of simulated microgravity. Therefore, our results provide an important basis for intestinal lncRNAs, such as the *linc-50*, in regulating the response to simulated microgravity in nematodes.

## Introduction

During or after spaceflight, some toxic effects on health of humans can be observed due to existence of microgravity and irradiation^1–5^. Nematode *Caenorhabditis elegans* is highly sensitive to various stresses or toxicants^6–10^. Due to this sensitivity, *C. elegans* has been frequently used to assess toxic effects of spaceflight on organisms^11–14^. Microgravity during the spaceflight potentially induced toxicity on locomotion behavior, muscle development, and reproduction in nematodes^11–14^. Moreover, microgravity during spaceflight could also induce dysregulation of some genes involved in control of metabolisms, cytoskeletal development, and etc.^15–17^.

Investigations on effects of simulated microgravity can reflect the potential influences of microgravity during spaceflight to a great degree. In nematodes, treatment with simulated microgravity could inhibit locomotion behavior, induce intestinal damage, and activate oxidative stress reflected by reactive oxygen species (ROS) production^18–20^. Meanwhile, simulated microgravity also activated some protective responses, such as mitochondrial unfolded protein response (mt UPR)^21^. More importantly, *C. elegans* is a powerful animal model for the study of molecular toxicology^22^. In *C. elegans*, insulin, p38 mitogen-activated protein kinase (MAPK), and Wnt signaling pathways have been identified to be involved in controlling toxicity of simulated microgravity^23–25^. Nevertheless, the molecular basis for response of nematodes to simulated microgravity still remains largely unclear.

Long non-coding RNAs (lncRNAs) are molecules having at least 200 nucleotides. The lncRNAs are a type of noncoding RNAs with many important biological functions^26,27^. In *C. elegans*, some of lncRNAs have been identified to be required for control of response to environmental toxicants, such as nanopolystyrene and graphene oxide (GO)^28,29^. We assumed that lncRNAs play important functions in regulating response to simulated microgravity. In *C. elegans*, our previous studies have suggested the crucial function of intestinal barrier in response to simulated microgravity^21,23–25^. In this study, we focused on the identification of intestinal lncRNAs involved in control of response to simulated microgravity. Moreover, we determined the underlying mechanism for *linc-50*, one of the identified lncRNAs, in regulating response to simulated microgravity.

## Results

### Effect of simulated microgravity treatment on ROS production and locomotion behavior in wild-type nematodes

**Figure 1:**
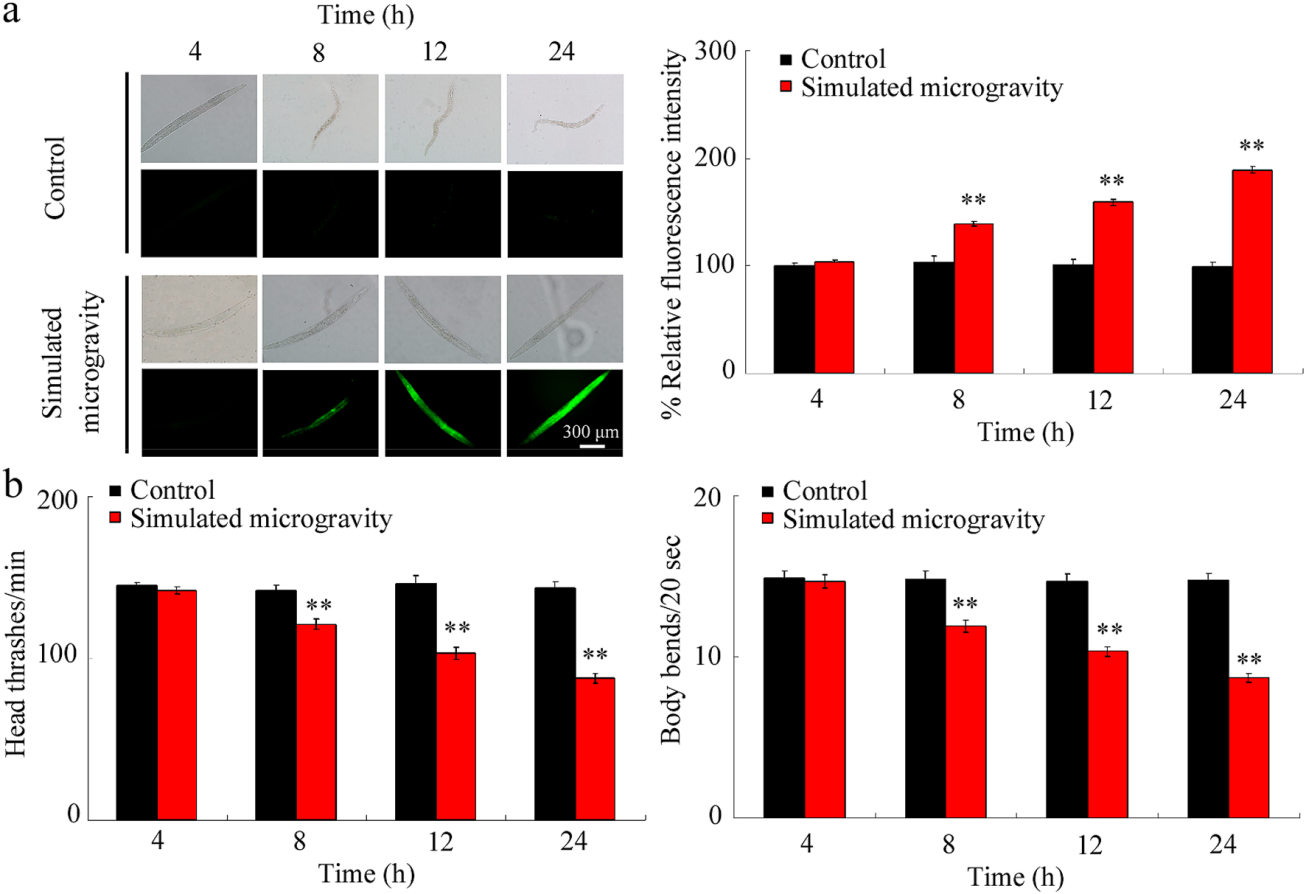
Effects of simulated microgravity treatment on wild-type nematodes. (**a**) Effects of simulated microgravity treatment on ROS production. N = 50. (**b**) Effects of simulated microgravity treatment on locomotion behavior. N = 40. Simulated microgravity treatment was performed in RCCS system at 30 rpm for 4, 8, 12, or 24 h. Bars represent means ± SD. **P < 0.01 *vs* control.

We first investigated the effects of simulated microgravity treatment for 4, 8, 12, or 24 h on two aspects, induction of ROS production and locomotion behavior. Simulated microgravity treatment for 4 h did not induce significant ROS production and alteration in locomotion behavior (Fig. 1; Table S1). In contrast, simulated microgravity for 8, 12, or 24 h could cause significant ROS production and decrease in locomotion behavior (Fig. 1; Table S1).

### Effect of simulated microgravity on expressions of intestinal lncRNAs

**Figure 2:**
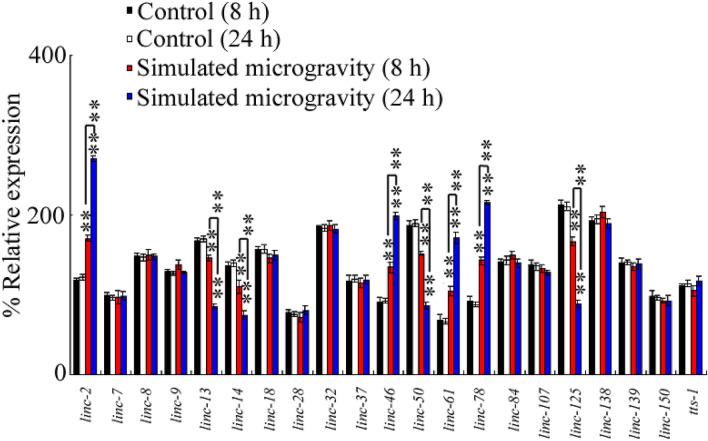
Effect of simulated microgravity on expressions of intestinal lncRNAs in wild-type nematodes. N = 3. Simulated microgravity treatment was performed in RCCS system at 30 rpm for 8 or 24 h. Bars represent means ± SD. **P < 0.01 control (8 or 24 h) (if not specially indicated).

In nematodes, some lncRNAs can be expressed in the intestine^28–31^. We further determined the effect of simulated microgravity for 8 h and 24 h on expressions of these intestinal lncRNAs in wild-type nematodes. Among these intestinal lncRNAs, simulated microgravity for 8 or 24 h significantly increased expressions of *linc-2*, *linc-46*, *linc-61*, and *linc-78*, and decreased expressions of *linc-13*, *linc-14*, *linc-50*, and *linc-125* (Fig. 2; Table S2). Moreover, simulated microgravity for 24 h caused more significant increase in expressions of *linc-2*, *linc-46*, *linc-61*, and *linc-78* and decrease in expressions of *linc-13*, *linc-14*, *linc-50*, and *linc-125* compared with those in nematodes treated with simulated microgravity for 8 h (Fig. 2; Table S2).

### Effect of RNAi knockdown of *linc-2, linc-13, linc-14, linc-46, linc-50, linc-61, linc-78,* or *linc-125* on toxicity of simulated microgravity

Using ROS production and locomotion behavior as the endpoints, we next examined the effect of RNAi knockdown of *linc-2*, *linc-13*, *linc-14*, *linc-46*, *linc-50*, *linc-61*, *linc-78*, or *linc-125* on toxicity of simulated microgravity in wild-type nematodes. Under the normal conditions, RNAi knockdown of *linc-2*, *linc-13*, *linc-14*, *linc-46*, *linc-50*, *linc-61*, *linc-78*, or *linc-125* did not induce the obvious ROS production and affect the locomotion behavior (Fig. S1; Table S3). After simulated microgravity treatment, RNAi knockdown of *linc-46*, *linc-78*, or *linc-125* did not noticeably influence the toxicity of simulated microgravity in inducing ROS production and in decreasing locomotion behavior (Fig. S1; Table S3). Different from this, after simulated microgravity treatment, RNAi knockdown of *linc-2* or *linc-61* led to the more severe toxicity in inducing ROS production and in decreasing locomotion behavior compared with those in wild-type nematodes (Fig. S1; Table S3), suggesting the susceptibility to toxicity of simulated microgravity in *linc-2(RNAi)* and *linc-61(RNAi)* nematodes. Additionally, although simulated microgravity for 24 h did not affect brood size in wild-type nematodes, we observed the significant reduction in brood size in *linc-2(RNAi)* and *linc-61(RNAi)* nematodes (Fig. S2; Table S4). In contrast, RNAi knockdown of *linc-13*, *linc-14*, or *linc-50* suppressed the toxicity of simulated microgravity (Fig. S1; Table S3), suggesting the resistance to toxicity of simulated microgravity in *linc-13(RNAi)*, *linc-14(RNAi)*, and *linc-50(RNAi)* nematodes. Data on efficiency for RNAi knockdown of *linc-2*, *linc-13*, *linc-14*, *linc-46*, *linc-50*, *linc-61*, *linc-78*, or *linc-125* in wild-type nematodes is shown in Fig. S3 and Table S5.

### Intestine-specific RNAi knockdown of *linc-2, linc-13, linc-14, linc-50,* or *linc-61* affected the toxicity of simulated microgravity

**Figure 3:**
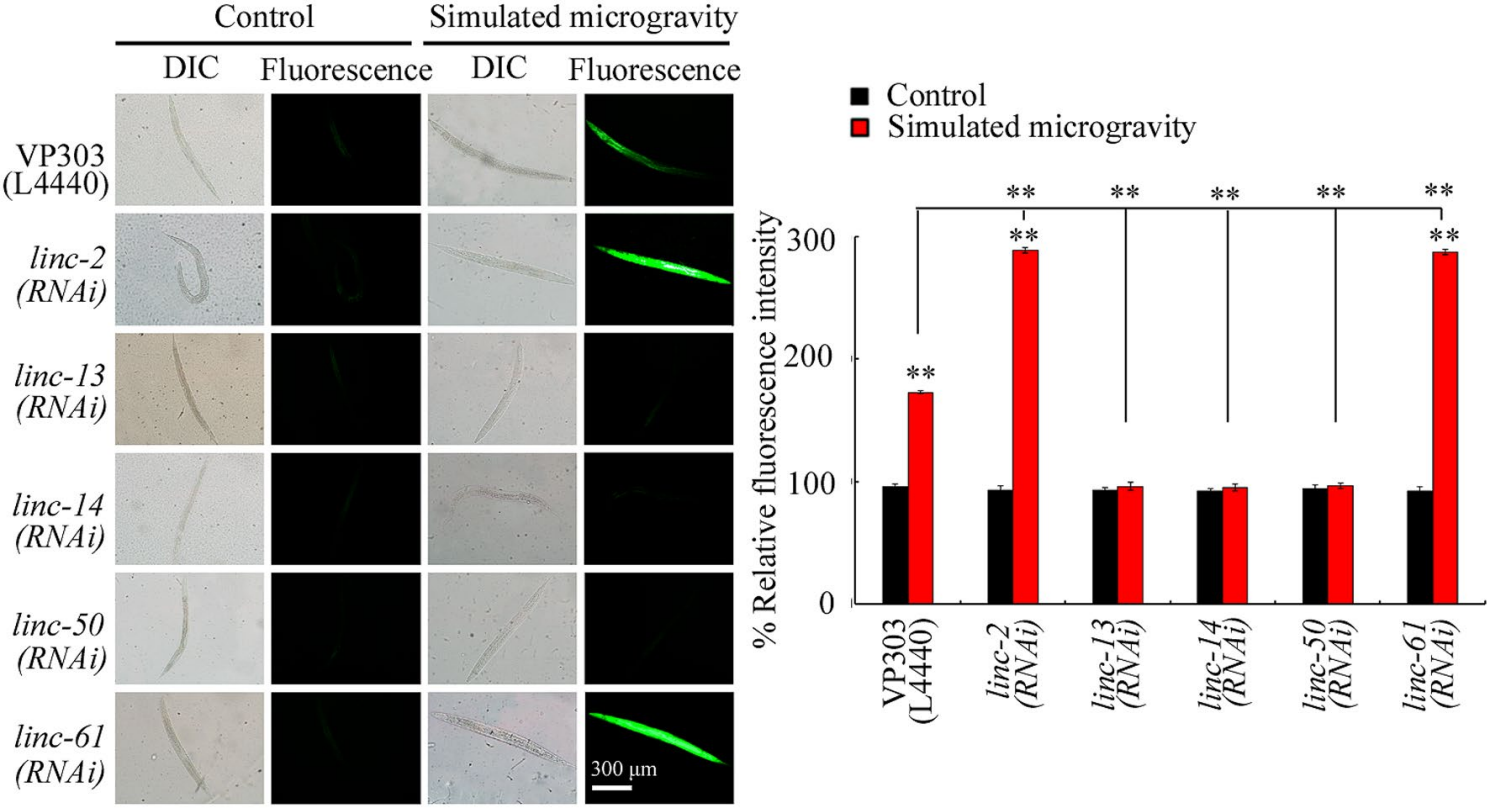
Effect of intestine-specific RNAi knockdown of *linc-2*, *linc-13*, *linc-14*, *linc-50*, or *linc-61* on ROS production in simulated microgravity treated nematodes. N = 50. L4440, empty vector. Simulated microgravity treatment was performed in RCCS system at 30 rpm for 24 h. Bars represent means ± SD. **P < 0.01 *vs* control (if not specially indicated).

We used VP303 strain to perform RNAi knockdown of certain lncRNA in the intestine^32^. ROS production was used as the endpoint, since the severe defect of locomotion is formed in VP303^32^. Intestinal RNAi knockdown of *linc-2* or *linc-61* enhanced the ROS production in simulated microgravity treated VP303 nematodes (Fig. 3; Table S6), suggesting the susceptibility to toxicity of simulated microgravity in animals with intestinal RNAi knockdown of *linc-2* or *linc-61*. However, intestinal RNAi knockdown of *linc-13, linc-14*, or *linc-50* suppressed the ROS production in simulated microgravity treated VP303 nematodes (Fig. 3; Table S6), suggesting the resistance to toxicity of simulated microgravity in animals with intestinal RNAi knockdown of *linc-13, linc-14*, or *linc-50*. Data on efficiency for RNAi knockdown of *linc-2*, *linc-13*, *linc-14*, *linc-50*, or *linc-61* in the intestine is shown in Fig. S3 and Table S5.

### Identification of dysregulated genes induced by intestine-specific RNAi knockdown of *linc-50* in simulated microgravity treated nematodes

**Figure 4:**
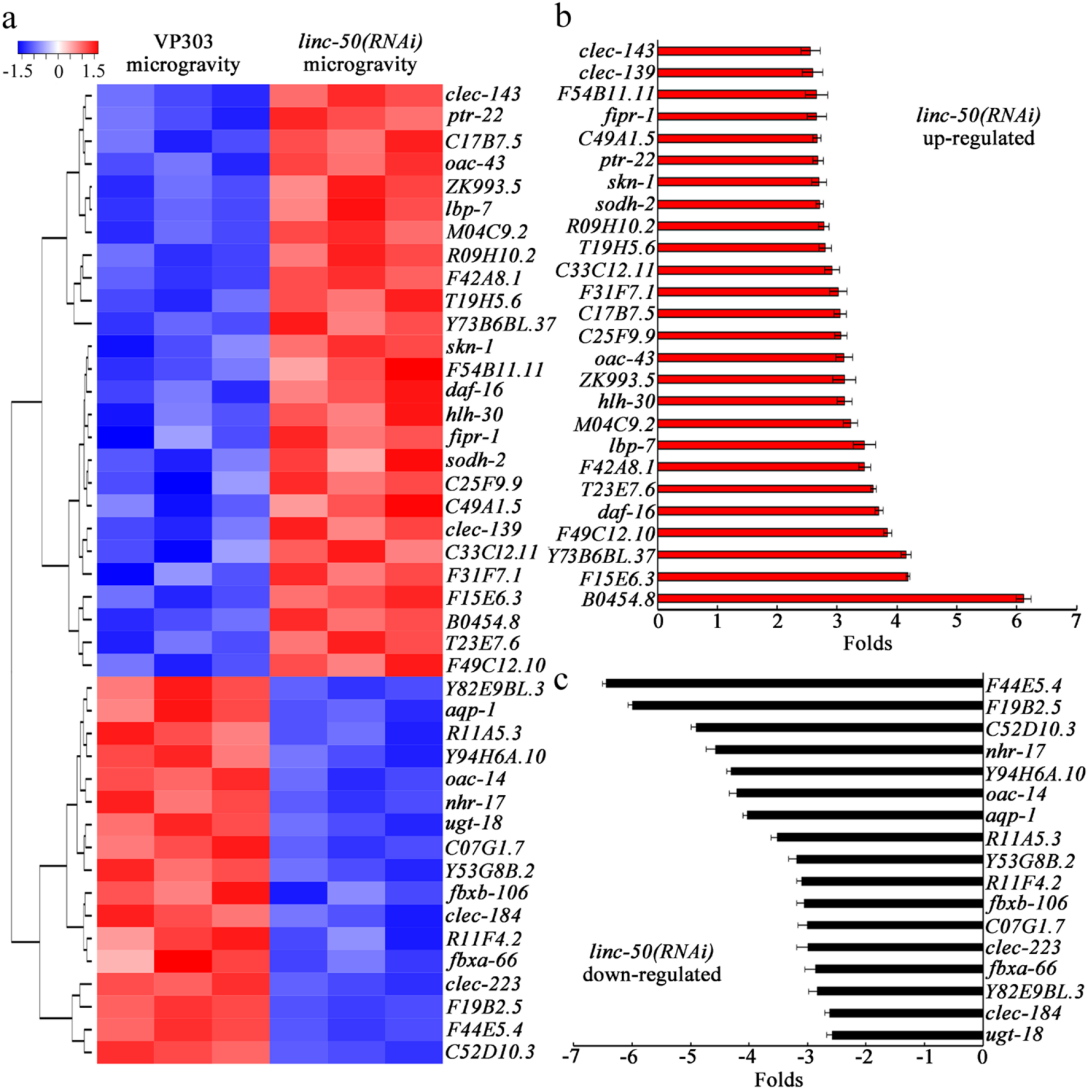
Identification of dysregulated genes by intestine-specific RNAi knockdown of *linc-50* in simulated microgravity treated nematodes. (**a**) Heatmap showing expression of dysregulated genes in simulated microgravity treated nematodes with intestine-specific RNAi knockdown of *linc-50*. The heatmap was drawn using Omicshare (www.omicshare.com/tools/), a free access online analysis tool of bioinformatics. (**b**) Upregulated genes induced by intestine-specific RNAi knockdown of *linc-50* in simulated microgravity treated nematodes. N = 3. (**c**) Downregulated genes induced by intestine-specific RNAi knockdown of *linc-50* in simulated microgravity treated nematodes. N = 3. Simulated microgravity treatment was performed in RCCS system at 30 rpm for 24 h.

We next focused on the identification of downstream genes of *linc-50*, one of the candidate lncRNAs whose RNAi knockdown induced a resistance to toxicity of simulated microgravity. Based on HiSeq 2000 sequencing, 43 genes were dysregulated by intestine-specific RNAi knockdown of *linc-50* in nematodes treated with simulated microgravity for 24 h (Fig. 4a; Table S7). Meanwhile, expressions of these 43 genes were also affected by simulated microgravity treatment (Table S8). Among these 43 genes, after simulated microgravity treatment, 26 genes (*clec-143*, *clec-139*, *F54B11.11*, *fipr-1*, *C49A1.5*, *ptr-22*, *skn-1*, *sodh-2*, *R09H10.2*, *T19H5.6*, *C33C12.11*, *F31F7.1*, *C17B7.5*, *C25F9.9*, *oac-43*, *ZK993.5*, *hlh-30*, *M04C9.2*, *lbp-7*, *F42A8.1*, *T23E7.6*, *daf-16*, *F49C12.10*, *Y73B6BL.37*, *F15E6.3*, and *B0454.8*) were upregulated, and 17 genes (*F44E5.4*, *F19B2.5*, *C52D10.3*, *nhr-17*, *Y94H6A.10*, *oac-14*, *aqp-1*, *R11A5.3*, *Y53G8B.2*, *R11F4.2*, *fbxa-106*, *C07G1.7*, *clec-223*, *fbxa-66*, *Y82E9BL.3*, *clec-184*, and *ugt-18*) were downregulated (Fig. 4b,c; Table S9).

### Potential transcriptional factors targeted by intestinal *linc-50* in simulated microgravity treated nematodes

**Figure 5:**
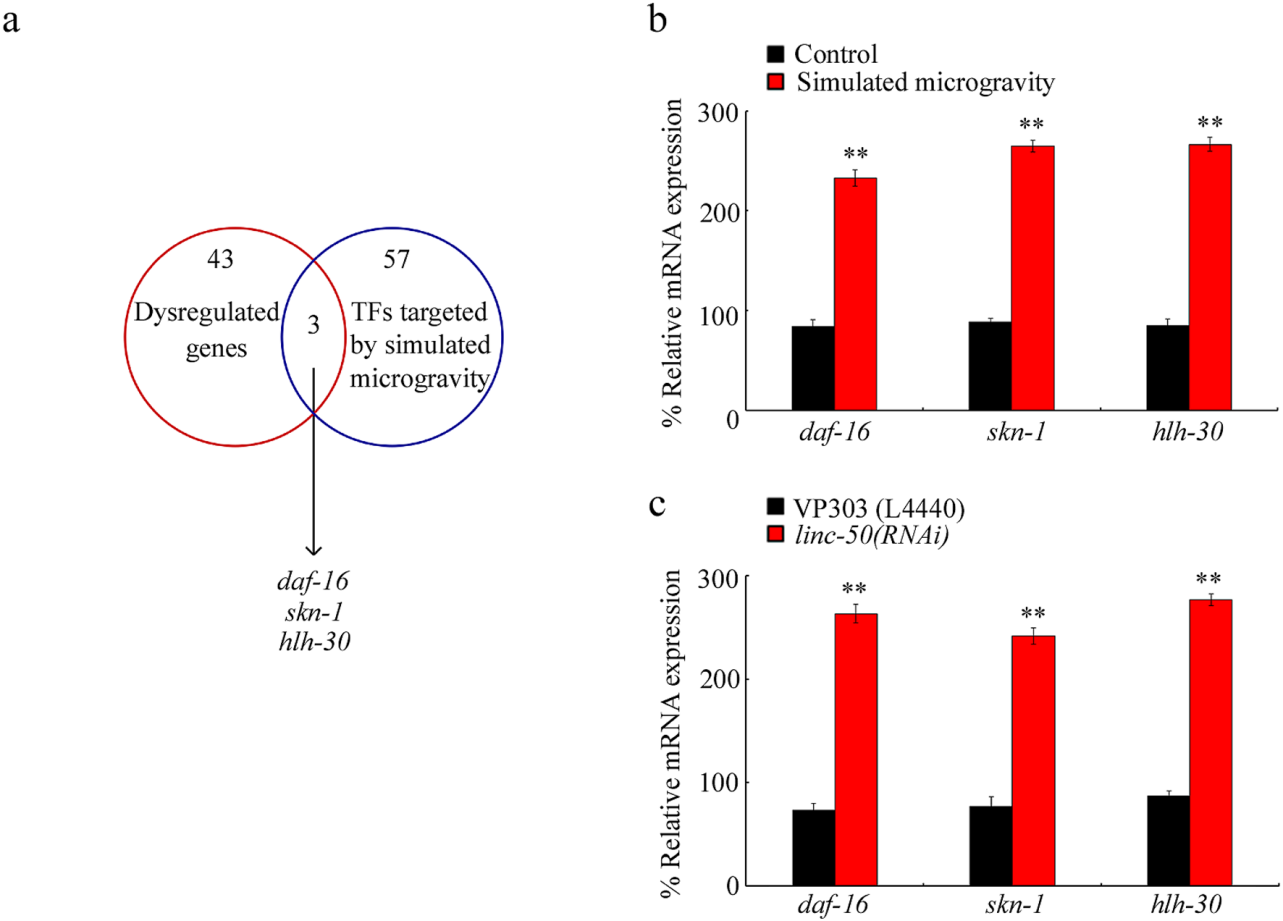
Screen of possible downstream transcriptional factors targeted by intestinal *linc-50* in simulated microgravity treated nematodes. (**a**) DAF-16, SKN-1, and HLH-30 are candidate transcriptional factors targeted by *linc-50* in simulated microgravity treated nematodes. TFs, transcriptional factors. (**b**) Effect of simulated microgravity on expressions of *daf-16*, *skn-1*, and *hlh-30*. N = 3. Bars represent means ± SD. **P < 0.01 *vs* control. (**c**) Effect of intestine-specific RNAi knockdown of *linc-50* on expressions of *daf-16*, *skn-1*, and *hlh-30* in simulated microgravity treated nematodes. N = 3. L4440, empty vector. Bars represent means ± SD. **P < 0.01 *vs* VP303. Simulated microgravity treatment was performed in RCCS system at 30 rpm for 24 h.

During the control of biological processes, lncRNAs can act as ‘ligands’ of transcriptional factors^33^. In nematodes, the candidate transcriptional factors targeted by lncRNAs have been identified by ChIP-SEQ technique to determine transcriptional factors binding site regions of lncRNAs, together with antibodies against transcriptional factors and immunoprecipitate nucleic acids^34,35^. Among the dysregulated genes induced by simulated microgravity treatment, 3 genes (*daf-16*, *skn-1*, and *hlh-30*) encode transcriptional factors potentially targeted by *linc-50* (Fig. 5a). The treatment with simulated microgravity could significantly increase expressions of *daf-16*, *skn-1*, and *hlh-30* (Fig. 5b; Table S10). Simulated microgravity treatment further increased both expression and nucleus localization of DAF-16::GFP in intestinal cells (Fig. S4a; Table S11). Similarly, the simulated microgravity also increased both expression and nucleus localization of SKN-1::GFP in intestinal cells (Fig. S4b; Table S11). Moreover, in simulated microgravity treated nematodes, intestine-specific RNAi knockdown of *linc-50* significantly increased expressions of *daf-16*, *skn-1*, and *hlh-30* (Fig. 5c; Table S10). Additionally, after simulated microgravity treatment, both expression and nucleus localization of DAF-16::GFP were increased by RNAi knockdown of *linc-50* (Fig. S4; Table S11). These data implied that DAF-16, SKN-1, and HLH-30 might function as the targeted transcriptional factors by intestinal *linc-50* in simulated microgravity treated nematodes. DAF-16 is a FOXO transcriptional factor in insulin signaling pathway involved in the regulation of response to simulated microgravity^21^. SKN-1 is a Nrf protein in p38 MAPK signaling pathway, and was required for the regulation of response to simulated microgravity^23^. HLH-30 is a transcriptional factor governing the autophagy induction, and required for the regulation of stress response^22^.

PRIdictor (protein–RNA interaction predictor) is a tool to predict mutual binding sites in RNA and protein at nucleotide or residue level (http://bclab.inha.ac.kr/pridictor). PRIdictor was further used to predict binding sites between *linc-50* and DAF-16, SKN-1, or HLH-30. The DAF-16 protein contains 25 possible nucleotide binding sites for *linc-50* (Fig. S5a), and the *linc-50* contains 40 possible residue binding sites for DAF-16 (Fig. S5b). The SKN-1 protein contains 25 possible nucleotide binding sites for *linc-50* (Fig. S5c), and the *linc-50* contains 82 possible residue binding sites for SKN-1 (Fig. S5d). The HLH-30 protein contains 2 possible nucleotide binding sites for *linc-50* (Fig. S5e), and the *linc-50* contains 5 possible residue binding sites for HLH-30 (Fig. S5f).

### Genetic interaction between *linc-50* and DAF-16, SKN-1, or HLH-30 in the intestine to regulate response to simulated microgravity

**Figure 6:**
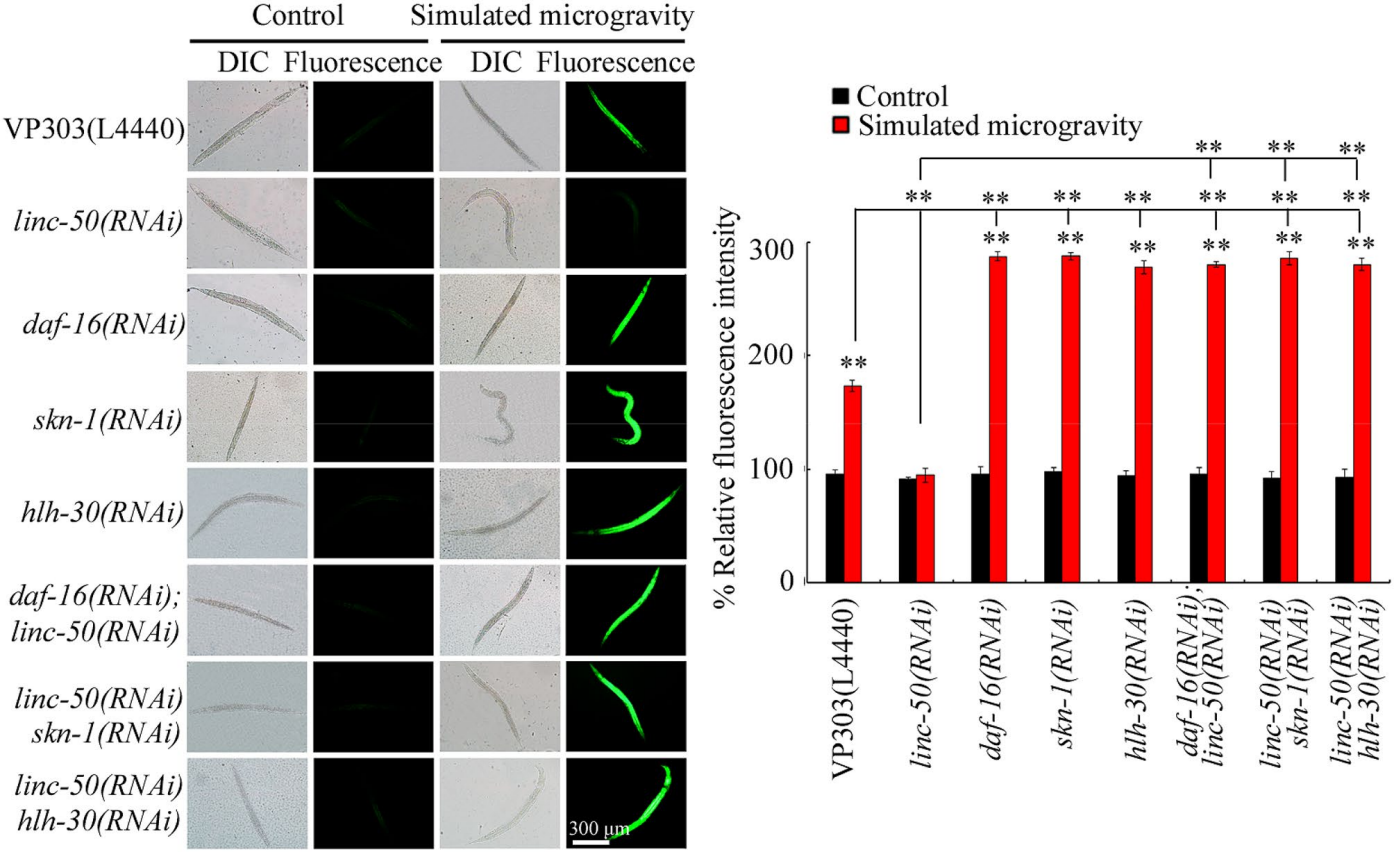
Genetic interaction between *linc-50* and DAF-16, SKN-1, or HLH-30 in the intestine to regulate ROS production in simulated microgravity treated nematodes. N = 50. L4440, empty vector. Simulated microgravity treatment was performed in RCCS system at 30 rpm for 24 h. Bars represent means ± SD. **P < 0.01 *vs* control (if not specially indicated).

RNAi knockdown of *daf-16*, *skn-1*, or *hlh-30* in the intestine increased ROS production in simulated microgravity treated VP303 nematodes (Fig. 6; Table S12), suggesting the susceptibility to toxicity of simulated microgravity in nematodes with intestinal RNAi knockdown of *daf-16*, *skn-1*, or *hlh-30*. Moreover, intestinal RNAi knockdown of *daf-16*, *skn-1*, or *hlh-30* inhibited the resistance to toxicity of simulated microgravity in *linc-50(RNAi)* nematodes (Fig. 6; Table S12). Therefore, in the intestine, DAF-16, SKN-1, and HLH-30 functioned downstream of *linc-50* to control response to simulated microgravity. Data on efficiency for intestinal RNAi knockdown of *linc-50*, *daf-16, skn-1*, or *hlh-30* is shown in Figs. S6, S7 and Tables S13, S14.

### Genetic interaction among DAF-16, SKN-1, and HLH-30 in the intestine to regulate response to simulated microgravity

**Figure 7:**
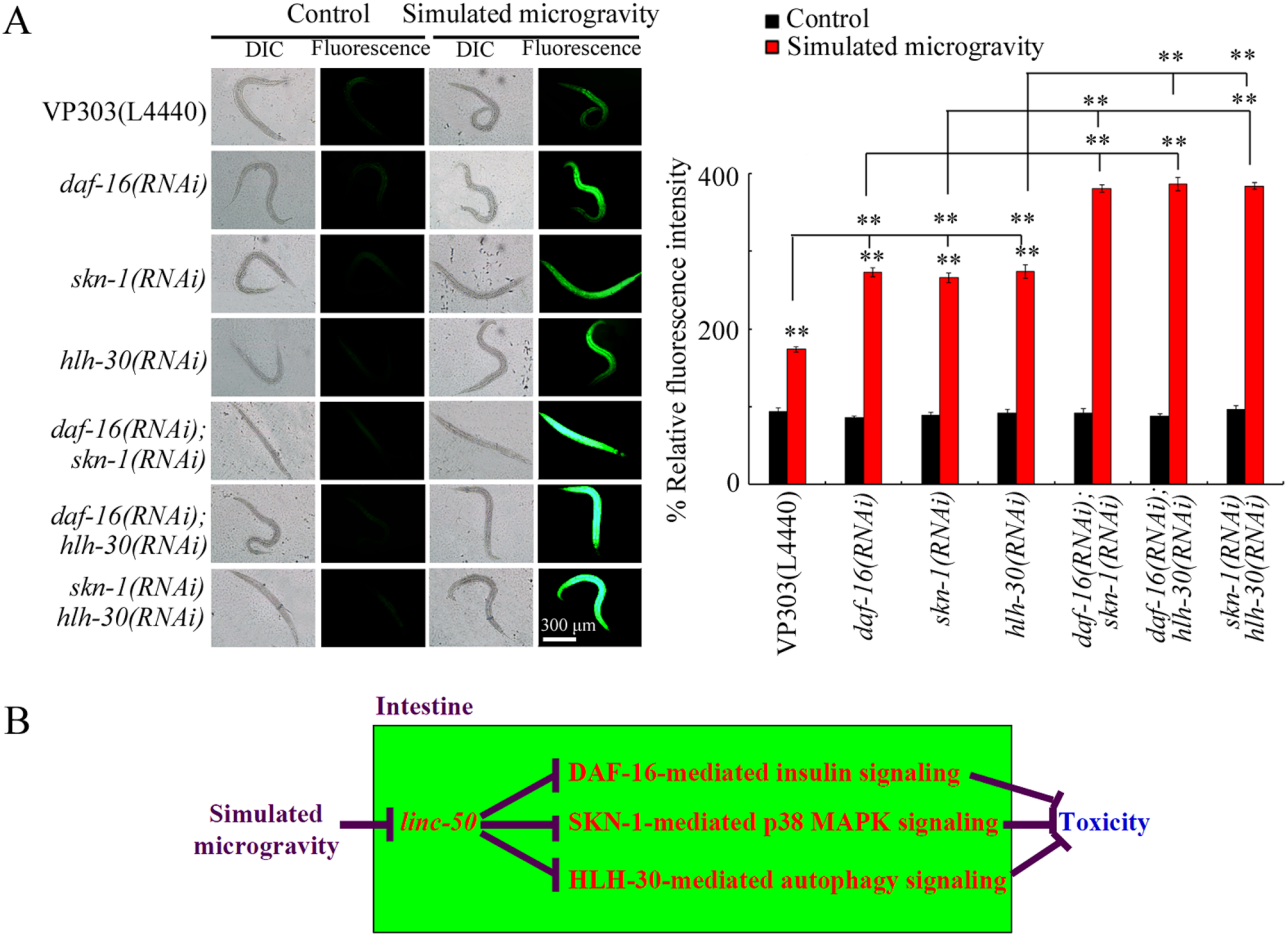
Genetic interaction among DAF-16, SKN-1, and HLH-30 in the intestine to regulate the response to simulated microgravity in nematodes. (**a**) Genetic interaction among DAF-16, SKN-1, and HLH-30 in the intestine to regulate the toxicity of simulated microgravity in inducing ROS production. N = 50. L4440, empty vector. Simulated microgravity treatment was performed in RCCS system at 30 rpm for 24 h. Bars represent means ± SD. **P < 0.01 *vs* control (if not specially indicated). (**b**) A diagram showing the molecular basis of intestinal *linc-50* in regulating the response to simulated microgravity in nematodes.

After simulated microgravity treatment, genetic interaction analysis indicated that double intestine-specific RNAi knockdown of *daf-16* and *skn-1* could cause the more severe ROS production than that in *daf-16(RNAi)* or *skn-1(RNAi)* nematodes (Fig. 7a; Table S15), suggesting that DAF-16 and SKN-1 acted in parallel pathways to regulate response to simulated microgravity. Similarly, after simulated microgravity treatment, double intestine-specific RNAi knockdown of *daf-16* and *hlh-30* could cause the more severe ROS production than that in *daf-16(RNAi)* or *hlh-30(RNAi)* nematodes (Fig. 7a; Table S15), suggesting that DAF-16 and HLH-30 also acted in parallel pathways to regulate response to simulated microgravity. Moreover, double intestine-specific RNAi knockdown of *skn-1* and *hlh-30* led to the more severe ROS production in simulated microgravity treated nematodes than that in simulated microgravity treated *skn-1(RNAi)* or *hlh-30(RNAi)* nematodes (Fig. 7a; Table S15), suggesting that SKN-1 and HLH-30 further acted in parallel pathways to regulate response to simulated microgravity. Data on efficiency for intestinal RNAi knockdown of *daf-16, skn-1*, or *hlh-30* is shown in Figs. S6, S7 and Tables S13, S14.

## Discussion

Our previous studies have implied the crucial role of intestinal barrier in response to simulated microgravity in nematodes^19,21,23–25^. Firstly, simulated microgravity could activate the protective responses (such as mt UPR) in intestinal cells^21^. Secondly, simulated microgravity could cause damage on intestinal barrier, such as the enhanced intestinal permeability^19^. Thirdly, some important signaling pathways (such as insulin, p38 MAPK, and Wnt signaling pathways) could be activated in the intestine, and were involved in the regulation of response to simulated microgravity^23–25^. Thus, in this study, we tried to identify the intestinal lncRNAs involved in the regulation of response to simulated microgravity in nematodes.

Among intestinal lncRNAs, simulated microgravity treatment for 8 or 24 h could increase expressions of *linc-2*, *linc-46*, *linc-61*, and *linc-78*, and decrease expressions of *linc-13*, *linc-14*, *linc-50*, and *linc-125* (Fig. 2; Table S2). Previous study has also suggested that exposure to 1 mg/L graphene oxide (GO) could decrease expression of *linc-14*^28^. Additionally, exposure to 1–100 μg/L nanopolystyrene (100 nm) could also increase expressions of *linc-2* and *linc-61*, and decrease *linc-50* expression^29^. These results implied the possible conserved property of *linc-2*, *linc-14*, *linc-50*, and *linc-61* in response to environmental toxicants or stresses.

Among the 8 dysregulated intestinal lncRNAs in simulated microgravity treated nematodes, we further observed the susceptibility of *linc-2(RNAi)* and *linc-61(RNAi)* nematodes to toxicity of simulated microgravity and the resistance of *linc-13(RNAi)*, *linc-14(RNAi)*, and *linc-50(RNAi)* nematodes to toxicity of simulated microgravity (Fig. S1; Table S3). These observations suggested that the alteration in expressions of *linc-2*, *linc-13*, *linc-14*, *linc-50*, and *linc-61* mediated a protective response to simulated microgravity. The resistance to toxicity of GO could also be detected in *linc-14(RNAi)* nematodes^28^. Additionally, RNAi knockdown of *linc-2* or *linc-61* could result in the susceptibility to toxicity of nanopolystyrene, and RNAi knockdown of *linc-50* caused the resistance to toxicity of nanopolystyrene^29^.

Moreover, our results demonstrated the important functions of *linc-2*, *linc-13*, *linc-14*, *linc-50*, and *linc-61* in the intestine to regulate response to simulated microgravity. Nevertheless, besides the expression in the intestine, *linc-2*, *linc-13*, *linc-50*, and *linc-61* can also be expressed in other tissues in nematodes^31^. Thus, we did not exclude the possibility that these 5 candidate lncRNAs can also act in other tissues to regulate the response to simulated microgravity.

For the *linc-50*, we found that it functioned upstream of three transcriptional factors (DAF-16, SKN-1, and HLH-30) to control response to simulated microgravity (Fig. 5a,b, Fig. S5; Table S10). In nematodes, DAF-16, SKN-1, and HLH-30 could act in the intestine to regulate response to simulated microgravity (Fig. 6; Table S12)^23,24^. Therefore, *linc-50*-DAF-16, *linc-50*-SKN-1, and *linc-50*-HLH-30 could be formed in the intestine to regulate response to simulated microgravity.

Moreover, our data suggested that intestinal *linc-50* regulated the response to simulated microgravity by targeting and suppressing transcriptional factors of DAF-16, SKN-1, and HLH-30 (Figs. 5c, 6; Tables S10, S12). Our results have indicated the important role of *linc-50* in response to both environmental stress and environmental toxicants^29^. These observations provided an important molecular basis for our understanding the role of *linc-50* in response to environmental stress or toxicants. Under the normal conditions, intestinal RNAi knockdown of *linc-50* could increase expressions of *daf-16* and *hlh-30*, but did not affect *skn-1* expression (Fig. S8; Table S16).

It was further found that DAF-16, SKN-1, and HLH-30 functioned synergistically to regulate response to simulated microgravity (Fig. 7a; Table S15). Therefore, intestinal *linc-50* regulated response to simulated microgravity by simultaneously suppressing functions of downstream DAF-16-medaied insulin signaling, SKN-1-mediated p38 MAPK signaling, and HLH-30-medaited autophagy signaling (Fig. 7b; Table S15). Insulin signaling, p38 MAPK signaling, and autophagy signaling reflect three forms of protective responses to environmental toxicants or stresses^7,22^, which further indicated the crucial role of intestinal *linc-50* in regulating the response to simulated microgravity.

In conclusion, we used *C. elegans* as an animal model to determine the functions of intestinal lncRNAs in regulating response to simulated microgravity stress. We identified 5 intestinal lincRNAs with protective functions during the control of response to simulated microgravity. Moreover, we found that intestinal *linc-50* could regulate response to simulated microgravity by suppressing functions of downstream three transcriptional factors (DAF-16, SKN-1, and HLH-30). Our study provides an important molecular basis for lncRNAs in the intestine to regulate response to environmental exposures in nematodes.

## Methods

### Strains and maintenance

The used wild-type nematodes are Bristol N2. Wild-type, *zIs356*[P*daf-16::daf-16a/b::GFP*], *ldEx9[skn-1::GFP]*, and VP303/*rde*-*1(ne219);kbIs7* strains were from *Caenorhabditis* Genetics Center (CGC). Nematodes were normally maintained on normal nematode growth mediate (NGM) plates fed with *Escherichia coli* OP50 as a food source^36^. To obtain synchronized L1-larvae or young adults, the gravid nematodes were treated with bleaching solution (5% NaOCl/1 N NaOH, 2:5) to release enough eggs. After that, the eggs were transferred onto another plate to allow the further development into L1-larvae or young adult population.

### Simulated microgravity

Simulated microgravity was carried out as described^19^. In cultivation chamber of the Rotary System (Synthecon), the vessels containing soft and movable 0.2% agar medium with suspended young adults were half filled. In vessels containing soft and movable 0.2% agar medium, no *E. coli* OP50 was added. The simulated microgravity was generated after balancing sedimentation-induced gravity with centrifugation by Rotary Cell Culture System (RCCS) vessel rotation. To prepare this balancing state, different vessels were placed symmetrically on RCCS system. To set up simulated microgravity, RCCS rotated chamber horizontally at 30 rpm for 4, 8, 12, or 24 h at 20 °C. Control young adults were grown in soft and movable 0.2% agar medium without simulated microgravity treatment at 20 °C.

### Quantitative real-time polymerase chain reaction (qRT-PCR)

After simulated microgravity treatment, total RNAs were isolated using Trizol (Sigma-Aldrich). Synthesis of cDNAs using reverse transcriptase reaction was prepared in Mastercycler gradient PCR system (Eppendorf). Transcriptional expressions of examined lncRNAs (*linc-2*, *linc-7*, *linc-8*, *linc-9*, *linc-13*, *linc-14*, *linc-18*, *linc-28*, *linc-32*, *linc-37*, *linc-46*, *linc-50*, *linc-61*, *linc-78*, *linc-107*, *linc-125*, *linc-138*, *linc-139*, *linc-150*, and *tts-1*) and genes (*daf-16*, *skn-1*, and *hlh-30*) were determined using SYBR Green qRT-PCR master mix (TOYOBO, Japan) in StepOnePlus real-time PCR system (Applied Biosystems). *tba-1* encoding an alpha-tubulin protein was used as a reference gene. Three replicates were carried out for all the reactions. Primer information for qRT-PCR T is shown in Table S17.

### RNA interference (RNAi)

*Escherichia coli* HT115 was cultured in LA broth (LB broth containing 100 μg/L ampicillin) with the addition of 5 mM isopropyl 1-thio-β-d-galactopyranoside (IPTG). L1-larvae were maintained on NGM plates fed with *E. coli* HT115 for RNAi knockdown of *linc-2*, *linc-13*, *linc-14*, *linc-46*, *linc-50*, *linc-61*, *linc-78*, *linc-125*, *daf-16*, *skn-1*, or *hlh-30*^37^. Once the nematodes developed into the gravid, they were transferred onto new RNAi plates. The second generation was used for simulated microgravity treatment. HT115 expressing empty vector L4440 was used as a control. Transgenic strain of VP303 is a genetic tool for intestinal RNAi knockdown of gene(s)^32^. qRT-PCR was performed to confirm RNAi knockdown efficiency.

### Toxicity assessment

Considering the sensitivity in assessing toxicity of simulated microgravity^18,23,24^, locomotion behavior and ROS production were used as toxicity assessment endpoints.

Locomotion behavior reflects functional state of motor neurons, and was evaluated by head thrash and body bend^38^. After the treatment, nematodes were washed using M9 buffer first. The nematodes were allowed for a 1-min recovery on surface of NMG plate. Under a dissecting microscopy, a head thrash is defined as an alteration in direction of posterior bulb part, and the head thrash frequency was recorded during the duration of 1 min. A body bend is defined as an alteration in direction of bending at the middle body, and the body bend frequency was recorded during the duration of 20 s. Forty nematodes were analyzed per treatment. Three replicates were performed.

ROS production was used to assess activation of oxidative stress in simulated microgravity treated nematodes^39,40^. After the treatment, nematodes were labeled with 1 µM CM-H_2_DCFDA for 3-h without light. After that, the nematodes were examined at 510 nm of emission filter and at 488 nm of excitation wavelength under a laser scanning confocal microscope (Leica, TCS SP2, Bensheim, Germany). Before the observations, the nematodes were paralyzed by adding sodium azide (0.01 M) on the agar pad. The 20 × objective was used for the observations. In nematodes, the strongest ROS fluorescent signals can be detected in the intestine^41^. Semi-quantification was determined for fluorescence intensity in the intestine in comparison to autofluorescence. Fifty animals were analyzed per treatment. Three replicates were performed.

Brood size was selected as an endpoint to reflect reproductive capacity^42^. To determine the brood size, number of offspring at all stages beyond the eggs was counted under a stereomicroscopy. For each treatment, thirty nematodes were examined. Three replicates were performed.

### HiSeq 2000 sequencing

HiSeq 2000 sequencing was used to identify dysregulated genes induced by intestinal RNAi knockdown of *linc-50* in simulated microgravity treated nematodes. The mRNA libraries were prepared with RNA-seq Sample Preparation kit (Illumina, Inc., USA) for Illumina HiSeq 2000 sequencing. Fast QC was used to check the quality of reads. Total read numbers of data sets were normalized to equal levels. The dysregulated genes were judged by statistical significance analysis and use of a 2.0-fold change cutoff.

### Statistical analysis

SPSS 12.0 was used for statistical analysis. One-way analysis of variance (ANOVA) was used to determine differences between groups. Two-way ANOVA analysis was used for multiple factor comparisons. Differences within groups were determined using Dunnett-*t* test, and probability level 0.01 was considered as statistically significant.

## Supplementary Information

Supplementary Information.
