# Supplementary material for: Intestinal long non-coding RNAs in response to simulated microgravity stress in Caenorhabditis elegans
Source: Sci Rep. 2021 Jan 21;11:1997. doi: 10.1038/s41598-021-81619-4 (PMC7820273; doi:10.1038/s41598-021-81619-4)
Supplement: Supplementary file 1 — Supplementary Information. [file 41598_2021_81619_MOESM1_ESM.pdf]

Intestinal long non-coding RNAs in response to simulated microgravity stress in *Caenorhabditis elegans*

Lingmei Sun, Dan Li, Yujie Yuan, Dayong Wang*

Medical School, Southeast University, Nanjing 210009, China

*Correspondence and requests for materials should be addressed to D.W (dayongw@seu.edu.cn).

Supporting Information:

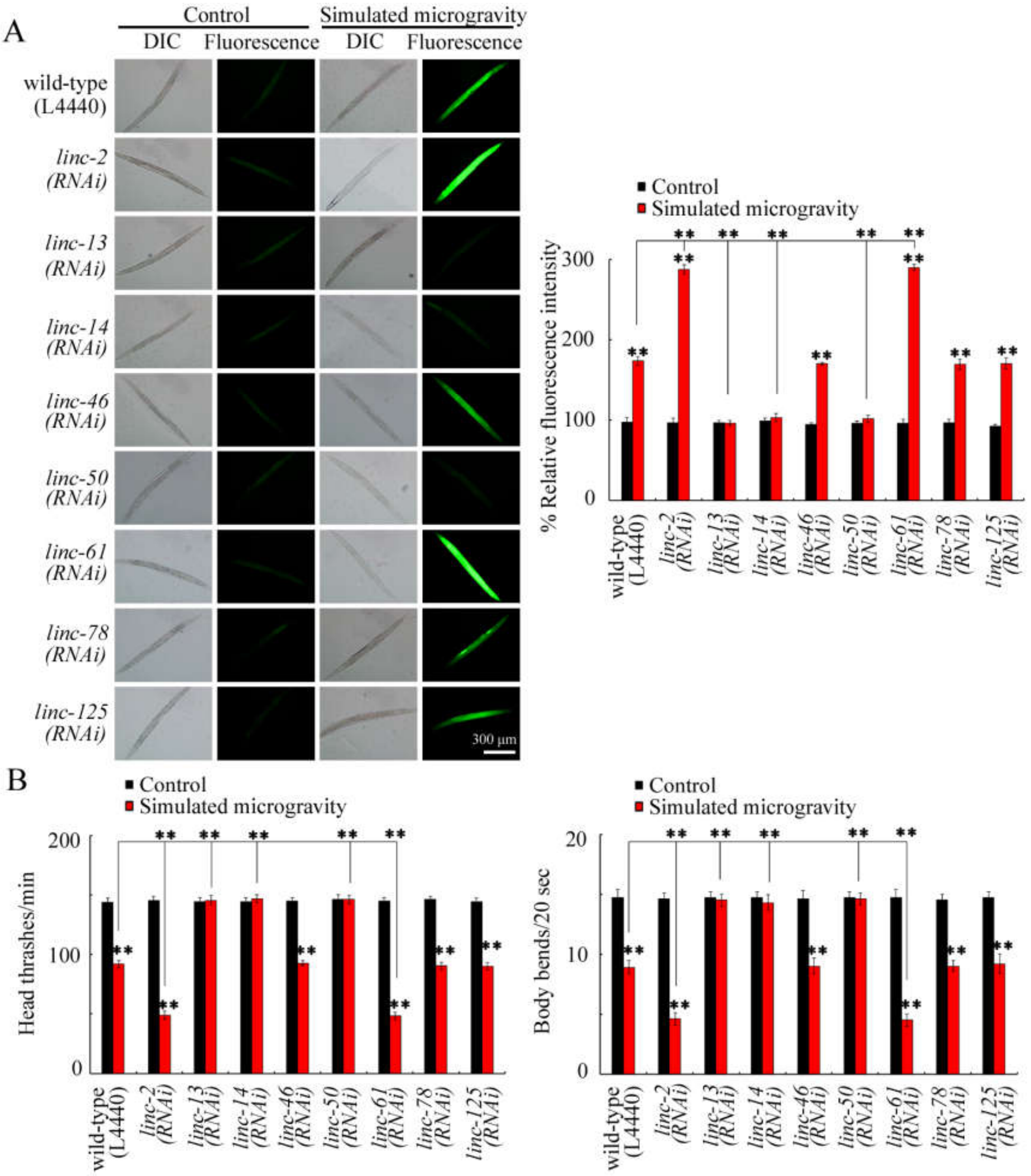

Figure S1. Effect of RNAi knockdown of *linc-2*, *linc-13*, *linc-14*, *linc-46*, *linc-50*, *linc-61*, *linc-78*, or *linc-125* on simulated microgravity treated wild-type nematodes. (a) Effect of RNAi knockdown of *linc-2*, *linc-13*, *linc-14*, *linc-46*, *linc-50*, *linc-61*, *linc-78*, or *linc-125* on ROS production in simulated microgravity treated wild-type nematodes. N = 50. (b) Effect of RNAi knockdown of *linc-2*, *linc-13*, *linc-14*, *linc-46*, *linc-50*, *linc-61*, *linc-78*, or *linc-125* on locomotion behavior in simulated microgravity treated wild-type nematodes. N = 40. L4440, empty vector. Simulated microgravity treatment was performed in RCCS system at 30 rpm for 24 h. Bars represent means ± SD. $^{**}P < 0.01$ *vs* control (if not specially indicated).

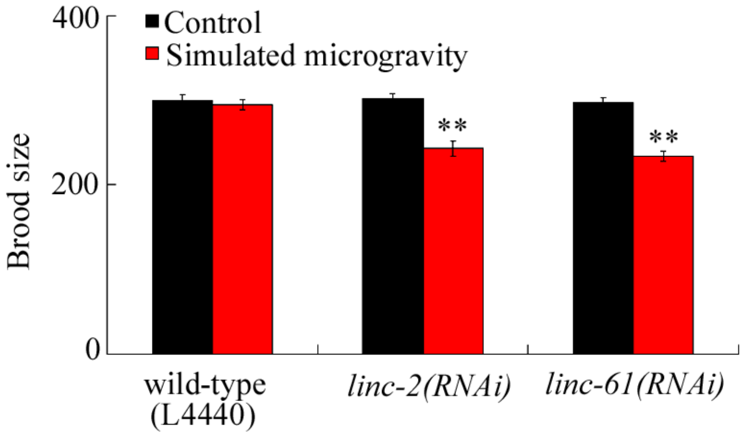

Figure S2. Effect of RNAi knockdown of *linc-2* or *linc-61* on brood size in simulated microgravity treated wild-type nematodes. N = 30. L4440, empty vector. Simulated microgravity treatment was performed in RCCS system at 30 rpm for 24 h. Bars represent means ± SD. $^{**}P < 0.01$ *vs* wild-type.

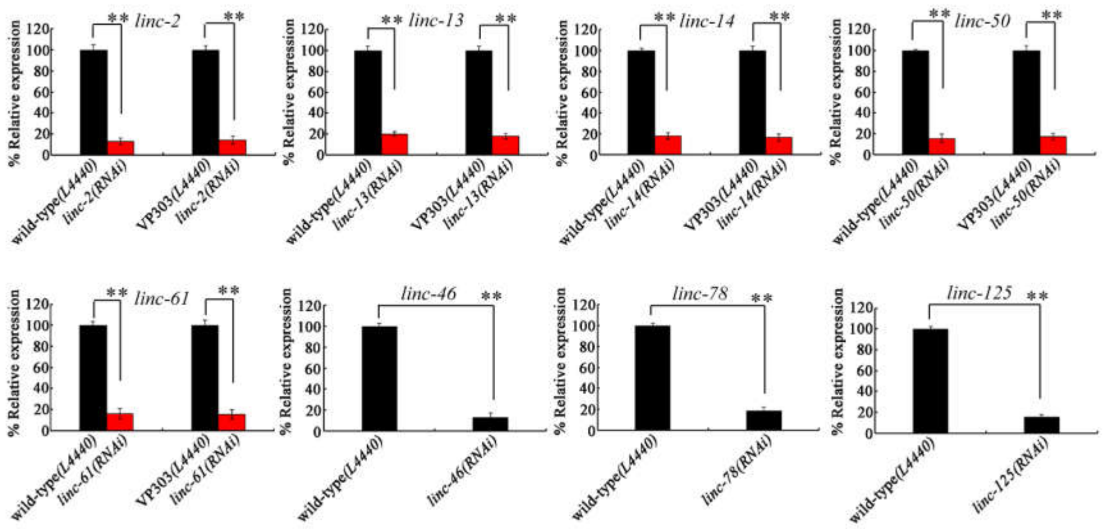

Figure S3. qRT-PCR analysis of efficiency for RNAi knockdown of *linc-2*, *linc-13*, *linc-14*, *linc-46*, *linc-50*, *linc-61*, *linc-78*, and *linc-125*. N = 3. L4440, empty vector. For RNAi knockdown of lncRNAs in wild-type nematodes, the total RNAs were extracted from the whole animals. For RNAi knockdown of lncRNAs in VP303 nematodes, the total RNAs were extracted from 30 isolated intact intestine. Bars represent means ± SD. $^{**}P < 0.01$.

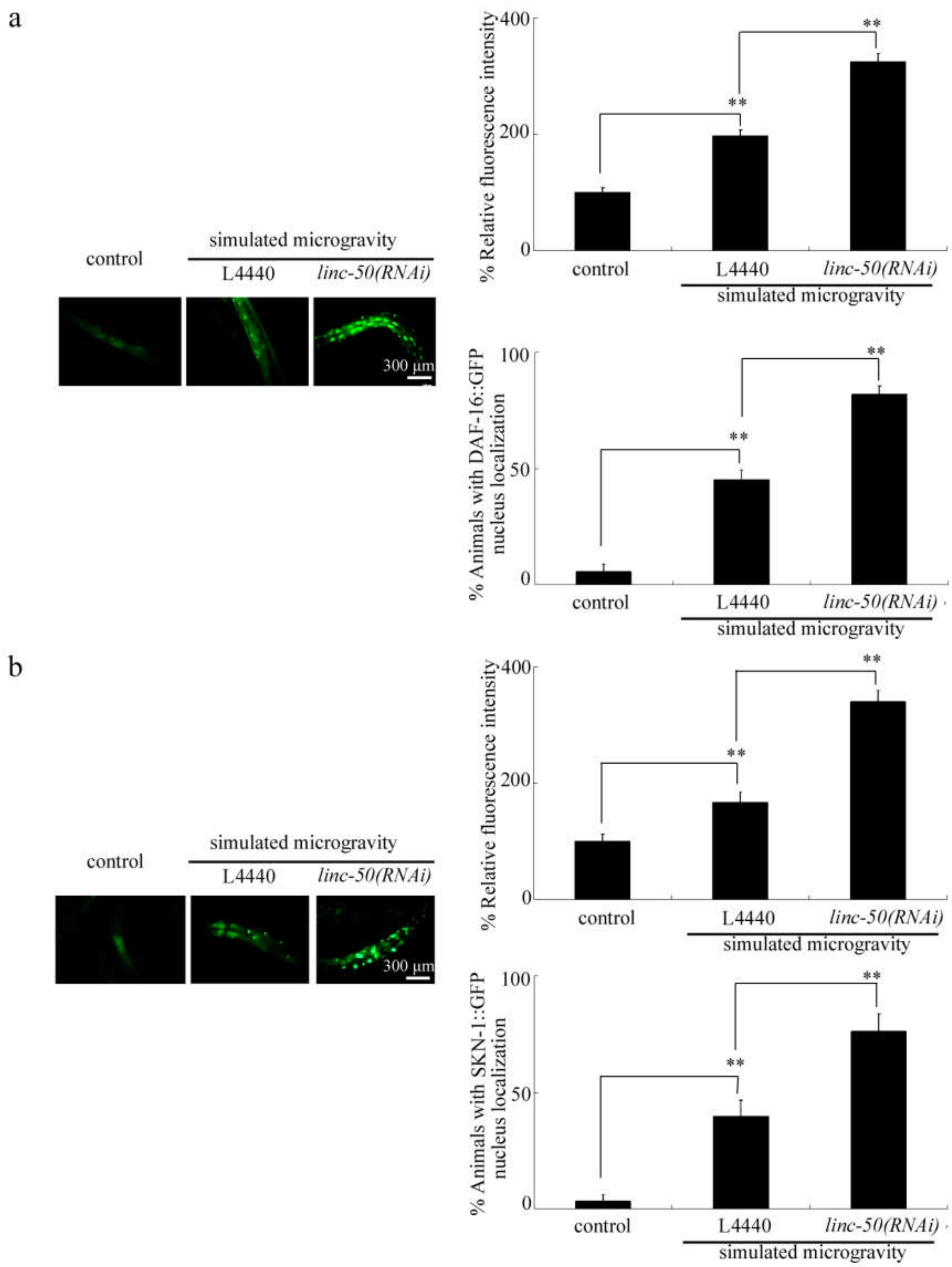

Figure S4. Effect of RNAi knockdown of *linc-50* on expression of DAF-16::GFP (a) or SKN-1::GFP (b) in simulated microgravity treated nematodes. N = 50. L4440, empty vector. Simulated microgravity treatment was performed in RCCS system at 30 rpm for 24 h. Bars represent means ± SD. $^{**}P < 0.01$.

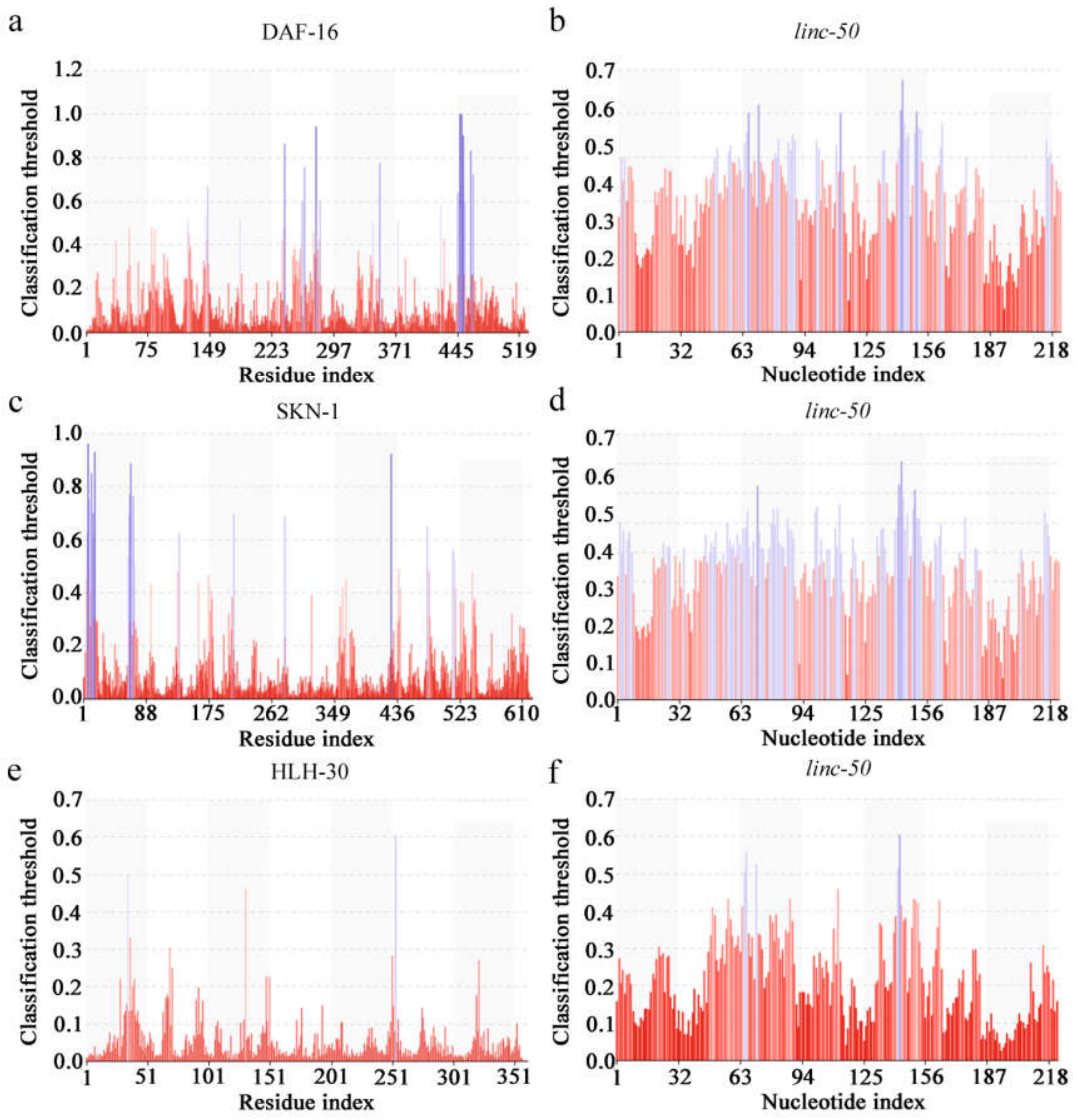

Figure S5. Possible binding sites between *linc-50* and DAF-16, SKN-1, or HLH-30. (A) Possible binding sites of *linc-50* in DAF-16. (B) Possible binding sites of DAF-16 in *linc-50*. (C) Possible binding sites of *linc-50* in SKN-1. (D) Possible binding sites of SKN-1 in *linc-50*. (E) Possible nucleotide binding sites of *linc-50* in HLH-30. (F) Possible nucleotide binding sites of HLH-30 in *linc-50*. Red color, non-binding; blue color, binding.

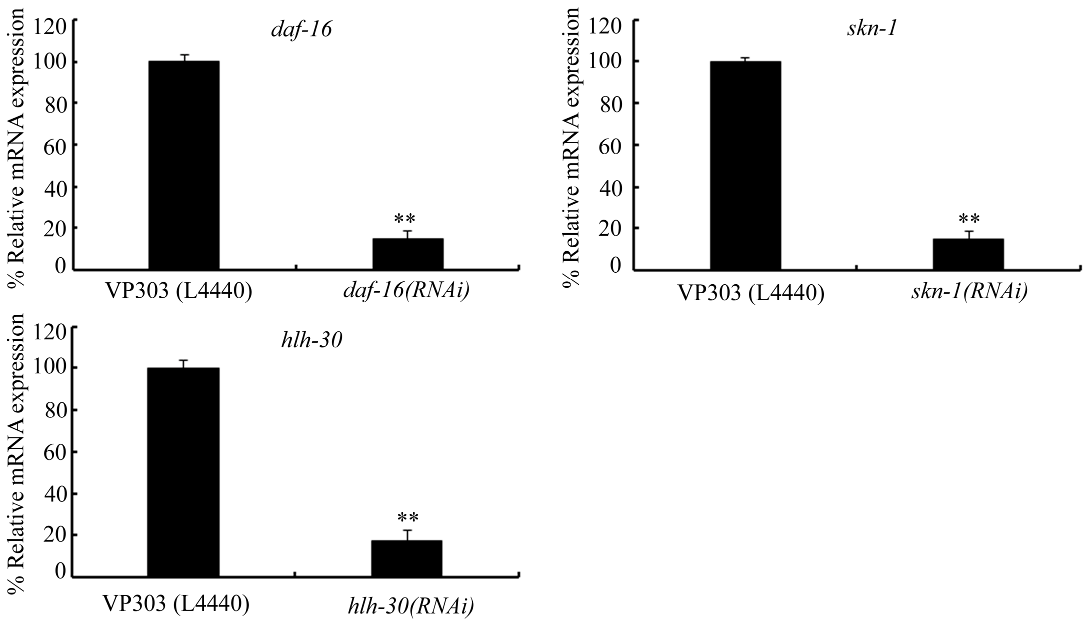

Figure S6. qRT-PCR analysis of efficiency for intestine-specific RNAi knockdown of *daf-16*, *skn-1*, or *hlh-30*. N = 3. L4440, empty vector. For RNAi knockdown of *daf-16*, *skn-1*, or *hlh-30* in VP303 nematodes, the total RNAs were extracted from 30 isolated intact intestine. Bars represent means ± SD. $^{**}P < 0.01$ *vs* VP303.

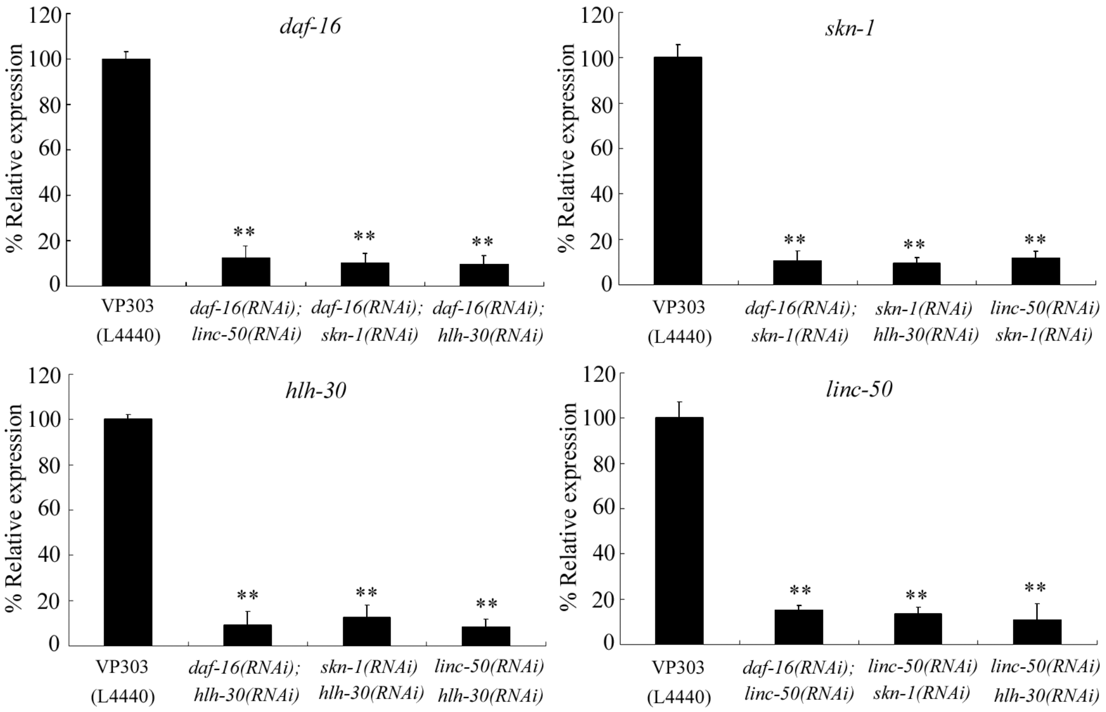

Figure S7. qRT-PCR analysis of efficiency for intestinal RNAi knockdown in *daf-16(RNAi);linc-50(RNAi)*, *daf-16(RNAi);skn-1(RNAi)*, *daf-16(RNAi);hlh-30(RNAi)*, *skn-1(RNAi)hlh-30(RNAi)*, *linc-50(RNAi)skn-1(RNAi)*, and *linc-50(RNAi)hlh-30(RNAi)* nematodes. N = 3. L4440, empty vector. For RNAi knockdown of genes in VP303 nematodes, the total RNAs were extracted from 30 isolated intact intestine. Bars represent means ± SD. $^{**}P < 0.01$ *vs* VP303.

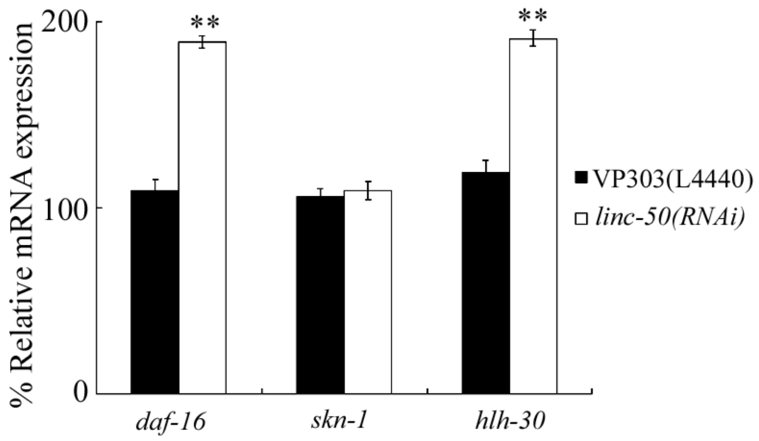

Figure S8. Effect of intestinal RNAi knockdown of *linc-50* on expressions of *daf-16*, *skn-1*, and *hlh-30* under the normal condition. N = 3. L4440, empty vector. Bars represent means ± SD. $^{**}P < 0.01$ *vs* VP303.

Table S1. Data on effects of simulated microgravity on ROS production and locomotion behavior

ROS production	4-h	8-8	12-h	24-h
Control<br>Simulated microgravity	100 ± 2.5<br>104 ± 1.2	103 ± 6<br>139 ± 2.6	101 ± 5<br>159 ± 3.2	99 ± 4.2<br>189 ± 3

Head thrash	4-h	8-8	12-h	24-h
Control<br>Simulated microgravity	145 ± 2.2<br>142.1 ± 2.4	142 ± 3.4<br>121.2 ± 3.2	146 ± 5<br>103 ± 3.7	143.5 ± 4.2<br>87.5 ±3.1
**Body bend**	**4-h**	**8-8**	**12-h**	**24-h**
Control<br>Simulated microgravity	14.9 ± 0.42<br>14.7 ± 0.41	14.8 ± 0.51<br>11.9 ± 0.36	14.7 ± 0.44<br>10.3 ± 0.31	14.8 ± 0.4<br>8.67 ± 0.27

Table S2. Data on effect of simulated microgravity on lncRNA expressions

	Control (8 h)	Control (24 h)	Simulated microgravity (8 h)	Simulated microgravity (24 h)
linc-2	119 ± 1.8	122 ± 4	171 ± 3.5	271 ± 3.4
linc-7	100 ± 3.2	97 ± 3	97 ± 8	99 ± 5.2
linc-8	148 ± 4.7	147 ± 5	150 ± 6.9	148 ± 3.4
linc-9	129 ± 3.5	127 ± 2.5	138 ± 5.3	128 ± 1.1
linc-13	168 ± 3.7	170 ± 3.3	146 ± 3.4	86 ± 3.3
linc-14	136 ± 5.1	140 ± 4.4	110 ± 8.4	75 ± 5.7
linc-18	157 ± 3.7	158 ± 5.5	146 ± 5.7	150 ± 5.3
linc-28	78 ± 3.5	76 ± 3	72 ± 5.4	81 ± 5
linc-32	186 ± 0.7	184 ± 4	187 ± 5.9	183 ± 5.3
linc-37	118 ± 5.8	120 ± 4	115 ± 6.2	119 ± 5.2
linc-46	91 ± 6.3	93 ± 3	127 ± 6.3	199 ± 4.6
linc-50	187 ± 5.9	190 ± 4.3	152 ± 2.4	87 ± 3.8
linc-61	69 ± 7.2	67 ± 3.4	92 ± 5.9	172 ± 7.1
linc-78	92 ± 6.3	88 ± 2.9	143 ± 4.7	216 ± 2.4
linc-84	141 ± 4.8	143 ± 5.4	150 ± 4.5	140 ± 5.2
linc-107	138 ± 5	136 ± 4.6	133 ± 5.2	128 ± 3.6
linc-125	182 ± 5.6	180 ± 5.4	167 ± 5.4	89 ± 5.4
linc-138	193 ± 5.4	195 ± 5.2	204 ± 6.7	190 ± 5.7
linc-139	140 ± 6.1	141 ± 3.2	135 ± 5.2	139 ± 6.8
linc-150	98 ± 6.8	97 ± 3.5	93 ± 3.3	94 ± 6.5
tts-1	111 ± 3	114 ± 3.8	105 ± 6.6	118 ± 5.6

Table S3. Data on effect of RNAi knockdown of *linc-2*, *linc-13*, *linc-14*, *linc-46*, *linc-50*, *linc-61*, *linc-78*, or *linc-125* on simulated microgravity treated wild-type nematodes

ROS production	Wild-type (L4440	*linc-2 (RNAi)*	*linc-13 (RNAi)*	*linc-14 (RNAi)*	*linc-46 (RNAi)*	*linc-50 (RNAi)*	*linc-61 (RNAi)*	*linc-78 (RNAi)*	*linc-125 (RNAi)*
Control	97.4 ± 5.7	96.6 ± 5.2	96.4 ± 2.7	98.8 ± 3.6	94.2 ± 2.4	95.4 ± 3	96 ± 4.5	96.6 ± 4.3	92.2 ± 2.4
Simulated microgravity	173.8 ± 5.5	287.6 ± 5.8	95.8 ± 3.7	102.8 ± 4.7	170.4 ± 1.8	101.2 ± 4.6.	290 ± 4	169.8 ± 6.6	170.8 ± 7
Head thrash	Wild-type (L4440	*linc-2 (RNAi)*	*linc-13 (RNAi)*	*linc-14 (RNAi)*	*linc-46 (RNAi)*	*linc-50 (RNAi)*	*linc-61 (RNAi)*	*linc-78 (RNAi)*	*linc-125 (RNAi)*
Control	143.7 ± 3.3	145.5 ± 3.2	144.3 ± 3.5	143.9 ± 3.1	144.9 ± 2.8	146.4 ± 3.7	144.2 ± 2.4	146.1 ± 2.3	144.5 ± 2.9
Simulated microgravity	91.8 ± 3.2	48.9 ± 3.4	145.5 ± 4.3	146.7 ± 3.3	92.7 ± 2.2	146.1 ± 3.4	48.3 ± 3.1	90.3 ± 2.8	90 ± 2.7
Body bend	Wild-type (L4440	*linc-2 (RNAi)*	*linc-13 (RNAi)*	*linc-14 (RNAi)*	*linc-46 (RNAi)*	*linc-50 (RNAi)*	*linc-61 (RNAi)*	*linc-78 (RNAi)*	*linc-125 (RNAi)*
Control	14.7 ± 0.6	14.6 ± 0.5	14.7 ± 0.4	14.7 ± 0.3	14.6 ± 0.7	14.7 ± 0.5	14.7 ± 0.7	14.5 ± 0.5	14.7 ± 0.4
Simulated microgravity	8.9 ± 0.6	4.6 ± 0.5	14.5 ± 0.5	14.3 ± 0.7	9 ± 0.7	14.6 ± 0.5	4.5 ± 0.5	9 ± 0.4	9.2 ± 0.8

Table S4. Data on effect of RNAi knockdown of *linc-2* or *linc-61* on brood size in simulated microgravity treated wild-type nematodes

	wild-type(L4440)	*linc-2(RNAi)*	*linc-61(RNAi)*
Control	300 ± 7	302 ± 6	298 ± 5
Simulated microgravity	295 ± 6	243 ± 9	234 ± 5.3

Table S5. Data on qRT-PCR analysis of efficiency for RNAi knockdown of *linc-2*, *linc-13*, *linc-14*, *linc-46*, *linc-50*, *linc-61*, *linc-78*, and *linc-125*

	wild-type(L4440)	*linc-2(RNAi)*
linc-2	100 ± 4.7	12.8 ± 2.9
	VP303(L4440)	*linc-2(RNAi)*
linc-2	100 ± 4.5	13.9 ± 3.7
	wild-type(L4440)	*linc-13(RNAi)*
linc-13	100 ± 4.2	19.9 ± 2
	VP303(L4440)	*linc-13(RNAi)*
linc-13	100 ± 3.9	17.7 ± 2.3
	wild-type(L4440)	*linc-14RNAi)*
linc-14	100 ± 2	17.8 ± 3.3
	VP303(L4440)	*linc-14(RNAi)*
linc-14	100 ± 4.1	16.3 ± 3.6
	wild-type(L4440)	*linc-50(RNAi)*
linc-50	100 ± 1.3	15.4 ± 4.2
	VP303(L4440)	*linc-50(RNAi)*
linc-50	100 ± 4.7	17.5 ± 3.2
	wild-type(L4440)	*linc-61(RNAi)*
linc-61	100 ± 3.9	15.9 ± 5.1
	VP303(L4440)	*linc-61(RNAi)*
linc-61	100 ± 5.2	15.6 ± 4.4
	wild-type(L4440)	*linc-46(RNAi)*
linc-46	100 ± 3.2	12.6 ± 4.5
	wild-type(L4440)	*linc-78(RNAi)*
linc-78	100 ± 2.5	18.2 ± 4.2
	wild-type(L4440)	*linc-125(RNAi)*
linc-125	100 ± 2.7	15.3 ± 2.7

Table S6. Data on effect of intestinal RNAi knockdown of linc-2, linc-13, linc-14, linc-50, or linc-61 on ROS production

	Control	Simulated microgravity
VP303(L4440)	95.8 ± 2.3	172.8 ± 1.1
linc-2(RNAi)	93 ± 3.7	289.4 ± 2.2
linc-13(RNAi)	92.6 ± 2.7	96 ± 3.1
linc-14(RNAi)	92 ± 2	94.8 ± 2.9
linc-50(RNAi)	94.6 ± 2.6	96.2 ± 2.2
linc-61(RNAi)	92.1 ± 3.5	287.8 ± 2.2

Table S7. Dysregulated genes by intestine-specific RNAi knockdown of *linc-50* in simulated microgravity treated nematodes based on HiSeq 2000 sequencing analysis

	VP303 (simulated microgravity)			*Linc-50 (RNAi)* (simulated microgravity)			
Gene name	#1	#2	#3	#1	#2	#3	logFC
clec-143	0.122	0.184	0.142	0.939	0.999	0.772	2.595
clec-139	0.114	0.165	0.186	1.241	0.602	0.903	2.561
F54B11.11	0.45	0.614	0.601	3.944	3.45	3.049	2.649
fipr-1	11.622	15.277	15.221	89.395	96.365	98.35	2.754
C49A1.5	0.987	0.234	0.234	1.916	2.901	3.924	2.587
ptr-22	0.817	0.823	0.9	5.658	4.338	3.998	2.462
skn-1	1.233	1.562	1.432	7.899	10.343	9.566	2.717
sodh-2	0.211	0.202	0.134	1.206	1.243	1.472	2.842
R09H10.2	0.547	0.57	0.345	3.19	3.391	3.094	2.727
T19H5.6	1.562	1.442	1.002	8.878	8.884	8.841	2.731
C33C12.11	0.433	0.715	0.733	5.724	5.78	5.774	3.199
F31F7.1	15.413	16.561	17.613	144.218	140.775	144.3	3.114
C17B7.5	0.058	0.057	0.056	0.799	0.521	0.532	3.443
C25F9.9	0.263	0.234	0.224	2.45	2.75	2.475	3.412
oac-43	0.237	0.212	0.267	1.889	2.324	2.243	3.174
ZK993.5	0.093	0.092	0.091	0.802	0.877	0.824	3.178
hlh-30	0.232	0.231	0.223	2.243	2.153	2.142	3.251
M04C9.2	0.23	0.18	0.23	2.612	2.132	2.132	3.423
lbp-7	0.734	0.762	0.713	8.845	8.649	8.896	3.579
F42A8.1	0.712	0.714	0.727	8.338	8.338	8.338	3.538
T23E7.6	4.835	4.118	4.398	57.533	55.995	55.51	3.662
daf-16	3.778	3.564	3.456	44.233	44.267	45.68	3.635
F49C12.10	2.424	2.436	2.442	36.112	36.104	36.93	3.902
Y73B6BL.37	0.556	0.535	0.593	10.545	10.785	10.86	4.257
F15E6.3	3.466	3.662	3.487	64.855	64.375	64.84	4.192
B0454.8	0.043	0.05	0.042	3.166	3.284	3.008	6.132
F44E5.4	407.56	406.601	405.256	4.751	4.751	4.751	-6.419
F19B2.5	326.87	333.911	336.909	4.51	5.56	5.657	-5.987
C52D10.3	62.916	61.901	60.961	2.223	2.232	2.134	-4.817
nhr-174	156.575	146.549	143.592	7.435	7.346	7.143	-4.349
Y94H6A.10	175.342	178.162	172.163	8.78	8.219	8.239	-4.381
oac-14	13.457	13.217	13.748	0.776	0.732	0.746	-4.164
aqp-1	86.605	85.611	85.606	6.222	4.212	5.218	-4.042
R11A5.3	30.119	30.239	30.94	2.567	2.78	2.742	-3.496
Y53G8B.2	40.677	41.367	42.386	4.888	4.238	4.75	-3.165
R11F4.2	8.566	8.122	8.921	0.919	0.978	0.979	-3.155
fbxb-106	1.232	1.311	1.355	0.126	0.156	0.161	-3.14

C07G1.7	2.11	2.022	2.055	0.224	0.213	0.25	-3.172
clec-223	19.13	19.1	19.24	2.996	2.896	2.56	-2.765
fbxa-66	17.844	16.811	15.806	2.662	2.342	2.202	-2.808
Y82E9BL.3	5.33	5.25	5.008	0.787	0.798	0.711	-2.763
clec-184	0.822	0.879	0.882	0.121	0.123	0.128	-2.793
ugt-18	40.565	43.456	40.483	6.293	6.113	6.934	-2.686

Note: $P < 0.05$

Table S8. Dysregulated genes by simulated microgravity treatment based on HiSeq 2000 sequencing analysis

Gene name	VP303 (control) #1	#2	#3	VP303 (simulated microgravity) #1	#2	#3	logFC
abf-2	0.422	0.411	0.46	0	0	0	-6.052
abt-4	0	0	0	0.108	0.18	0.176	8.278
abu-3	0	0	0	0.222	0.212	0.223	5.716
abu-4	0.179	0.156	0.131	0	0	0	-5.547
acr-5	0.021	0.022	0.021	0.123	0.135	0.199	3.024
lgc-11	0	0	0	0.121	0.131	0.113	5.716
acy-3	0	0	0	0.057	0.06	0.055	6.194
ads-1	0.438	0.464	0.984	0	0	0	-8.236
alh-1	0	0	0	0.096	0.096	0.096	5.716
alh-9	0	0	0	0.171	0.167	0.117	6.194
ape-1	0	0	0	0.226	0.283	0.248	7.765
apn-1	0.012	0.013	0.015	0.228	0.228	0.28	3.925
43922	0.033	0.032	0.034	0	0	0	-5.547
aqp-1	1.786	1.456	1.039	0.134	0.156	0.159	-2.803
arl-6	0.222	0.233	0.233	0	0	0	-5.547
aars-1	0	0	0	0.062	0.062	0.062	5.716
arx-1	0	0	0	0.139	0.199	0.138	6.194
arx-3	0	0	0	0.135	0.191	0.142	5.974
pah-1	0.443	0.445	0.426	6.174	6.714	6.143	3.728
bli-1	0.333	0.232	0.115	0	0	0	-6.89
bpl-1	0.034	0.042	0.046	0	0	0	-5.902
brc-1	0.323	0.444	0.116	0	0	0	-6.311
btf-1	0.033	0.034	0.036	0	0	0	-6.052
bub-1	0.044	0.033	0.05	0	0	0	-5.735
cah-4	0	0	0	0.232	0.272	0.234	6.384
cal-2	1.433	1.545	1.079	0.123	0.113	0.129	-3.142
cal-3	0.122	0.433	0.157	0	0	0	-5.547
cca-1	0	0	0	0.073	0.075	0.073	7.184
cct-4	0	0	0	0.188	0.179	0.123	6.194
cdc-42	0	0	0	0.118	0.18	0.189	5.974
cdh-4	0.004	0.003	0.001	0.052	0.025	0.024	3.789
cdr-1	0.032	0.045	0.042	0.201	0.277	0.291	2.569
ced-10	0	0	0	0.102	0.122	0.103	5.716
ced-12	0	0	0	0.177	0.172	0.128	6.838
ceh-10	1.223	1.453	1.339	0.031	0.033	0.03	-5.478
ceh-19	0.742	0.821	0.604	0	0	0	-7.177
ceh-20	0.663	0.663	0.297	0.073	0.072	0.076	-2.044
ceh-33	0.133	0.124	0.163	0	0	0	-5.547

chs-2	0.024	0.023	0.025	0	0	0	-5.547
chk-2	1.443	1.255	1.294	0.223	0.233	0.252	-2.465
ckb-2	0.134	0.167	0.101	0	0	0	-5.547
cki-2	0.132	0.231	0.12	0	0	0	-5.902
clc-4	0.554	0.664	0.358	0	0	0	-6.187
cnd-1	0.122	0.122	0.17	0	0	0	-5.547
col-17	1.35	1.68	1.098	0.049	0.046	0.089	-3.692
col-41	0.442	0.662	0.153	0	0	0	-6.187
col-48	0.767	0.834	0.69	0.034	0.033	0.036	-4.259
col-73	2.341	2.561	2.122	0.423	0.412	0.499	-2.196
col-74	0.324	0.367	0.455	0.052	0.053	0.06	-2.982
col-81	0.145	0.157	0.183	0	0	0	-6.052
col-97	0	0	0	0.132	0.122	0.137	5.716
col-101	0.558	0.878	0.767	0.144	0.132	0.122	-2.739
col-104	0	0	0	0.134	0.185	0.185	5.716
col-109	0	0	0	0.123	0.143	0.133	5.716
col-125	0.233	0.223	0.282	0	0	0	-6.531
col-152	0.042	0.022	0.021	0.432	0.432	0.317	3.638
col-153	0.062	0.061	0.064	0.432	0.443	0.439	2.613
col-166	0.434	0.422	0.35	0.061	0.061	0.063	-2.533
coq-6	0	0	0	0.334	0.392	0.332	7.184
csb-1	0	0	0	0.044	0.047	0.05	5.716
csn-1	0	0	0	0.141	0.115	0.12	6.194
csn-2	0.033	0.037	0.035	0.25	0.234	0.298	2.918
ctl-2	0.078	0.088	0.093	0.456	0.487	0.418	2.03
cwp-4	1.441	1.661	1.109	0.21	0.223	0.288	-2.052
cyb-3	0.053	0.054	0.052	0.324	0.323	0.358	2.613
cye-1	0.13	0.056	0.099	0	0	0	-6.187
cyk-1	0.021	0.021	0.03	0	0	0	-5.547
cyk-4	0.054	0.064	0.041	0.246	0.356	0.252	2.471
cyn-12	0	0	0	0.65	0.5	0.865	7.375
daf-2	0.066	0.066	0.031	0	0	0	-6.187
daf-6	0	0	0	0.082	0.081	0.089	6.384
daf-7	0.155	0.177	0.127	0	0	0	-6.187
daf-14	0.445	0.555	0.535	0.123	0.124	0.105	-2.443
dao-3	0.125	0.124	0.153	1.922	1.912	1.921	3.511
deg-1	1.233	1.214	1.219	0.243	0.223	0.29	-2.18
dhs-3	0.032	0.032	0.038	0.745	0.732	0.749	4.082
dhs-9	0.123	0.155	0.166	0	0	0	-5.902
dhs-11	2.128	1.986	1.816	0	0	0	-9.12
dhs-17	0.244	0.255	0.116	0	0	0	-5.902
dhs-22	0	0	0	0.352	0.32	0.305	6.838
dim-1	0.334	0.737	0.207	0	0	0	-7.471

dlc-1	0	0	0	0.256	0.286	0.264	5.716
dli-1	1.867	1.823	1.71	0.324	0.32	0.399	-2.207
dlk-1	0.034	0.045	0.043	0	0	0	-5.547
dnc-2	0.222	0.233	0.214	1.453	1.321	1.014	2.115
dnj-11	0.057	0.074	0.074	0	0	0	-5.735
dog-1	0.056	0.053	0.097	0	0	0	-6.722
dpf-7	0.023	0.06	0.061	0	0	0	-5.547
dpl-1	0.788	0.822	0.746	0.123	0.113	0.133	-2.585
dpy-27	0	0	0	0.034	0.032	0.034	5.716
dsc-1	0	0	0	0.412	0.418	0.441	7.078
dsl-4	0	0	0	0.124	0.125	0.127	5.716
dsl-5	0	0	0	0.285	0.248	0.209	5.974
duo-3	0.112	0.091	0.1	0	0	0	-6.722
dyf-5	0.453	0.567	0.265	0	0	0	-7.302
eat-16	0.251	0.205	0.295	0	0	0	-7.999
eat-20	0	0	0	0.044	0.074	0.07	6.194
ech-5	0.331	0.188	0.122	0	0	0	-5.902
efk-1	0.322	0.658	0.198	0	0	0	-7.523
egl-9	0	0	0	0.525	0.247	0.152	7.078
egl-19	0	0	0	0.027	0.028	0.03	5.974
egl-27	0	0	0	0.106	0.121	0.131	7.375
eif-3.F	0	0	0	0.143	0.313	0.13	5.716
elo-1	0.031	0.033	0.035	8.084	8.038	8.068	7.613
elo-3	0	0	0	1.047	1.015	1.081	8.371
elt-3	0.046	0.023	0.057	0.212	0.233	0.292	2.201
emb-30	0.055	0.088	0.047	0	0	0	-5.902
eor-2	0.069	0.079	0.089	0.513	0.516	0.579	2.566
epi-1	0.001	0.001	0.002	0.046	0.048	0.049	4.549
epn-1	0.017	0.018	0.018	0.123	0.112	0.189	3.149
etr-1	0	0	0	0.276	0.176	0.113	6.384
exc-4	0.433	0.565	0.261	0	0	0	-6.311
exp-2	0.455	0.448	0.462	0.053	0.052	0.056	-3.108
far-1	0.083	0.088	0.087	0.51	0.543	0.594	2.598
far-5	47.335	42.454	46.729	4.37	4.17	4.702	-3.427
far-8	0	0	0	0.283	0.226	0.293	6.194
fat-2	0.016	0.017	0.015	0.433	0.466	0.436	4.549
fat-6	0.057	0.055	0.06	0.467	0.443	0.408	2.613
fkb-6	0.134	0.222	0.179	1.345	1.455	1.509	2.946
fkh-6	0.658	0.823	0.784	0.043	0.05	0.033	-4.536
flp-2	4.124	3.456	3.438	0	0	0	-9.438
flp-3	5.313	3.787	4.225	0	0	0	-10.013
flp-15	0	0	0	0.682	0.622	0.658	6.552
fog-2	0.178	0.189	0.164	0	0	0	-6.052

fre-1	0.034	0.023	0.03	0.124	0.121	0.156	2.187
frm-1	0.01	0.007	0.009	0	0	0	-5.547
frm-4	0	0	0	0.171	0.112	0.117	6.552
gab-1	0	0	0	0.213	0.227	0.221	6.963
gar-2	0.1	0.077	0.082	0	0	0	-6.052
gbh-1	0.121	0.113	0.112	0	0	0	-5.735
gcy-7	0.045	0.056	0.068	0	0	0	-6.311
gcy-22	3.023	3.331	3.086	0.32	0.234	0.201	-4.052
gei-1	0.034	0.033	0.031	0	0	0	-5.735
gex-3	0.055	0.034	0.036	0	0	0	-5.547
gfl-1	0.556	0.345	0.388	0	0	0	-6.63
glc-2	0.789	0.8	0.774	0.046	0.047	0.049	-4.032
glc-4	0	0	0	0.158	0.176	0.186	6.702
gln-3	0	0	0	1.651	1.047	1.048	8.893
gln-5	0.255	0.432	0.238	0	0	0	-6.722
gln-6	0.012	0.016	0.013	0.222	0.225	0.258	4.049
gly-8	0.094	0.054	0.097	0	0	0	-5.547
gly-11	0.224	0.443	0.155	0	0	0	-6.722
gob-1	0	0	0	0.284	0.237	0.236	7.184
gon-4	0	0	0	0.049	0.043	0.048	5.974
gpa-4	0.331	0.144	0.106	0	0	0	-6.187
gpb-1	1.779	1.923	1.861	0.31	0.335	0.391	-2.362
gro-1	0.134	0.144	0.178	0	0	0	-6.425
gst-2	0.322	0.452	0.203	0	0	0	-5.547
gst-7	0	0	0	0.232	0.232	0.22	5.716
gst-24	0	0	0	0.363	0.327	0.319	6.194
gst-25	1.938	0.346	0.939	0	0	0	-7.715
gst-27	0	0	0	0.232	0.242	0.219	5.716
gst-40	0	0	0	0.243	0.253	0.23	5.716
haf-6	0.443	0.344	0.279	0.057	0.052	0.06	-2.314
hda-2	0	0	0	0.116	0.255	0.155	6.384
hda-10	0.034	0.033	0.092	0	0	0	-5.735
hel-1	0.089	0.057	0.087	0	0	0	-5.547
hil-3	0	0	0	0.23	0.29	0.297	6.384
hil-4	0.245	0.433	0.254	0	0	0	-6.425
him-1	0.055	0.056	0.033	0	0	0	-5.547
him-10	0.332	0.332	0.11	0	0	0	-6.052
his-5	0	0	0	0.523	0.521	0.515	5.974
his-35	0.034	0.023	0.038	0.523	0.535	0.515	3.47
his-41	0.312	0.233	0.359	0	0	0	-6.052
hlh-12	0.432	0.443	0.664	0	0	0	-6.89
hlh-16	3.344	4.215	2.062	0	0	0	-8.385
hmg-3	4.479	4.457	4.793	1.433	1.422	1.115	-2.218

hmg-4	0	0	0	0.09	0.086	0.087	5.974
hop-1	0.111	0.101	0.132	0	0	0	-5.735
hot-5	0.223	0.213	0.339	0	0	0	-6.052
hpl-1	0	0	0	0.283	0.243	0.243	5.974
hst-2	0.046	0.047	0.043	0.433	0.322	0.319	2.713
hum-8	0	0	0	0.115	0.115	0.117	7.184
ifc-2	0.056	0.067	0.05	0	0	0	-6.187
ifp-1	0	0	0	0.023	0.083	0.088	6.194
ins-11	0	0	0	1.613	1.027	1.061	6.552
ins-13	0	0	0	3.223	3.523	3.225	7.832
ins-17	1.445	1.334	1.019	0.103	0.121	0.109	-3.28
ins-27	0.512	0.544	0.585	0	0	0	-6.531
ins-30	0	0	0	1.161	1.176	1.138	7.896
ins-37	0.223	0.222	0.297	0	0	0	-5.735
inx-7	0.333	0.322	0.169	0	0	0	-6.808
inx-10	0.308	0.322	0.377	0.081	0.081	0.083	-2.283
iars-2	0.066	0.074	0.041	0	0	0	-5.547
ist-1	0.195	0.168	0.168	0	0	0	-7.622
isw-1	0.044	0.066	0.069	0	0	0	-6.311
itx-1	0	0	0	0.035	0.036	0.032	5.716
kgb-1	0.102	0.122	0.145	0	0	0	-6.311
kin-20	0.027	0.028	0.026	0.332	0.212	0.191	2.713
sid-3	0	0	0	0.116	0.161	0.122	7.375
klc-2	0.043	0.035	0.031	0.223	0.213	0.282	3.003
klp-11	0.056	0.057	0.062	0	0	0	-6.311
klp-13	0.062	0.064	0.068	0	0	0	-5.735
klp-19	1.225	1.335	1.472	0.323	0.344	0.362	-2.136
kqt-2	2.331	2.233	2.077	0.533	0.567	0.523	-2.101
lad-2	0.078	0.067	0.047	0	0	0	-5.902
lam-1	0.023	0.012	0.026	0	0	0	-5.735
lat-2	0.045	0.056	0.044	0	0	0	-6.052
lbp-6	0	0	0	0.445	0.444	0.423	6.194
lec-1	0.145	0.154	0.126	0	0	0	-5.547
lec-5	0.133	0.144	0.127	0	0	0	-5.547
lec-10	0	0	0	0.262	0.272	0.221	5.716
let-4	1.579	0.351	0.551	0	0	0	-8.881
let-504	0.113	0.133	0.113	0	0	0	-6.052
let-607	0.064	0.054	0.045	0	0	0	-5.547
let-716	0.066	0.077	0.056	0	0	0	-6.722
let-721	0	0	0	0.101	0.105	0.113	6.194
lig-4	0.066	0.063	0.065	0	0	0	-5.735
lin-13	0.067	0.077	0.053	0.013	0.012	0.013	-2.044
lin-14	0	0	0	0.096	0.092	0.094	5.716

lin-17	0.023	0.032	0.026	0.221	0.123	0.134	2.187
lin-18	0.073	0.072	0.079	0	0	0	-5.735
lin-25	0.037	0.043	0.037	0	0	0	-5.547
lin-26	0	0	0	0.083	0.084	0.08	5.716
lin-33	0	0	0	0.148	0.144	0.181	5.716
lin-59	0.009	0.009	0.008	0.261	0.199	0.126	3.73
lir-3	1.223	1.883	1.294	0.345	0.325	0.311	-2.16
lis-1	0	0	0	0.16	0.16	0.147	6.384
lon-3	1.067	1.551	1.099	0.219	0.259	0.293	-2.009
lst-4	0.067	0.056	0.061	0	0	0	-5.547
lys-2	0.443	0.245	0.227	0	0	0	-6.187
lys-5	0.052	0.051	0.055	0.933	0.932	0.934	3.881
lys-6	0.357	0.389	0.322	0	0	0	-6.311
mab-9	0	0	0	0.18	0.176	0.143	5.974
mab-21	0.785	0.785	0.785	0	0	0	-8.413
mca-3	0.031	0.013	0.012	0.123	0.145	0.128	3.179
mec-2	0.134	0.155	0.105	0	0	0	-6.052
mec-9	0	0	0	0.053	0.054	0.058	5.716
mec-14	0.038	0.064	0.036	0.544	0.455	0.226	2.474
mef-2	0	0	0	0.123	0.133	0.135	5.716
mex-1	0	0	0	0.18	0.118	0.121	6.194
mig-2	0	0	0	0.248	0.28	0.295	6.702
mig-5	0	0	0	0.085	0.08	0.089	5.974
mig-14	0.012	0.013	0.01	0.12	0.22	0.195	3.925
mig-15	0	0	0	0.099	0.096	0.1	6.963
mir-81	1.658	1.668	1.763	0	0	0	-6.052
mir-239.1	0.718	0.728	0.777	19.212	17.663	18.265	4.388
mir-241	1.79	1.677	1.785	0	0	0	-5.902
mir-257	3.656	3.778	3.547	0	0	0	-6.968
mnm-2	0.552	0.544	0.214	0	0	0	-6.052
mod-1	0.089	0.081	0.089	0	0	0	-5.735
mps-1	0.332	0.328	0.32	0	0	0	-6.425
mrp-2	0	0	0	0.032	0.032	0.034	5.716
msp-33	1.564	1.766	1.366	0.072	0.076	0.071	-4.259
mtm-1	0.121	0.333	0.118	0	0	0	-6.311
mtm-3	0.009	0.01	0.01	0.072	0.071	0.074	2.737
mtm-9	0.544	0.512	0.558	0.052	0.051	0.052	-3.496
mua-3	0	0	0	0.013	0.012	0.014	5.716
mup-4	0	0	0	0.022	0.021	0.024	5.716
mut-2	4.455	4.988	4.062	0.899	0.823	0.846	-2.375
nas-6	0.243	0.344	0.238	0	0	0	-6.425
nas-9	0.086	0.087	0.087	0	0	0	-5.735
nas-25	0.082	0.082	0.085	0.324	0.456	0.414	2.133

ncl-1	0	0	0	0.048	0.043	0.044	5.974
ncs-1	0.309	0.311	0.34	0	0	0	-6.311
ncs-2	1.044	1.556	1.056	0.174	0.128	0.178	-2.668
ncx-3	0.013	0.013	0.014	0.091	0.091	0.095	2.569
dcap-2	0.056	0.057	0.058	0	0	0	-5.735
ndx-8	0.432	0.438	0.435	0	0	0	-6.89
ned-8	0.456	0.478	0.453	0	0	0	-6.187
nfi-1	0	0	0	0.183	0.135	0.118	6.838
nfm-1	0.066	0.055	0.056	0	0	0	-5.547
nhl-1	0	0	0	0.071	0.094	0.091	6.552
nhl-3	0.005	0.005	0.009	0.143	0.133	0.132	3.638
nhr-3	0.053	0.073	0.033	0.223	0.211	0.262	2.819
nhr-8	29.433	29.411	29.408	1.226	1.216	1.257	-4.662
nhr-14	0	0	0	0.446	0.462	0.405	7.896
nhr-22	0.007	0.009	0.01	0.346	0.325	0.395	5.044
nhr-28	0.037	0.044	0.04	0.323	0.324	0.355	3.003
nhr-35	0.006	0.006	0.01	0.177	0.176	0.171	3.789
nhr-58	0.443	0.357	0.256	0	0	0	-6.722
nhr-78	0	0	0	0.123	0.213	0.13	5.716
nhr-104	0.094	0.045	0.096	0	0	0	-5.735
nhr-114	1.233	1.212	1.846	0.216	0.316	0.16	-3.625
nhr-133	0.231	0.23	0.104	0	0	0	-5.547
nlp-2	0.491	0.538	0.92	0	0	0	-7.802
nlp-17	1.566	1.566	1.407	0.345	0.543	0.301	-2.314
nlp-18	0.233	0.201	0.246	0	0	0	-5.547
nlp-21	0.566	0.655	0.414	0	0	0	-6.808
nlp-22	0.127	0.447	0.684	0	0	0	-6.311
nlp-23	0.78	0.235	0.792	0	0	0	-7.111
nlp-24	0.845	0.338	0.802	3.99	3.457	3.974	2.186
nlp-27	0	0	0	0.227	0.233	0.27	5.716
nlp-29	0	0	0	0.533	0.517	0.531	5.974
nos-3	0.167	0.176	0.161	4.551	4.341	4.097	4.55
npp-8	0.008	0.007	0.009	0.092	0.035	0.091	3.149
npp-11	0	0	0	0.157	0.173	0.146	6.963
ntl-2	0	0	0	0.103	0.114	0.104	5.974
oma-1	0.078	0.085	0.085	0	0	0	-5.735
osm-8	1.485	0.83	0.49	0	0	0	-7.574
oxi-1	0.424	0.412	0.459	0.014	0.013	0.019	-4.616
pab-2	0	0	0	0.053	0.053	0.051	5.716
pak-1	0	0	0	0.095	0.095	0.098	6.194
pat-3	0.005	0.006	0.007	0.113	0.123	0.125	3.925
pat-12	0.034	0.033	0.039	0	0	0	-5.547
pct-1	0.235	0.299	0.249	0.038	0.046	0.039	-2.762

pdi-2	0	0	0	0.179	0.148	0.105	5.974
pdr-1	0	0	0	0.158	0.158	0.193	6.384
pek-1	0	0	0	0.194	0.141	0.179	7.695
pen-2	0.062	0.061	0.062	0.412	0.42	0.426	2.569
pgp-2	0.021	0.012	0.014	0.09	0.069	0.085	2.474
pho-4	0.133	0.122	0.108	0	0	0	-5.902
pif-1	0.356	0.312	0.288	0.053	0.052	0.058	-2.401
pkc-1	0.168	0.198	0.158	0	0	0	-7.177
pkc-3	0.013	0.012	0.016	0.113	0.122	0.112	2.569
pll-1	0.145	0.157	0.177	0.022	0.023	0.029	-2.672
pmk-2	0	0	0	0.182	0.122	0.148	6.194
pmp-4	0	0	0	0.082	0.085	0.084	5.974
pmr-1	0.076	0.045	0.07	0	0	0	-6.311
ppw-2	0.078	0.089	0.065	0	0	0	-6.187
pqn-18	0.007	0.008	0.009	0.113	0.114	0.178	4.049
pqn-21	0.213	0.223	0.275	0.032	0.034	0.031	-3.239
pqn-32	0.808	0.776	0.709	0.013	0.022	0.017	-5.401
ptrn-1	0.035	0.034	0.04	0	0	0	-5.735
pqn-41	0.022	0.031	0.012	0.067	0.057	0.066	2.315
ifet-1	0.055	0.056	0.047	0	0	0	-5.735
abu-13	0	0	0	0.289	0.289	0.213	6.384
pqn-54	0.134	0.167	0.165	0	0	0	-6.187
larp-5	0.045	0.045	0.048	0	0	0	-5.735
pqn-65	0	0	0	0.032	0.032	0.031	5.716
pqn-74	2.456	2.677	2.328	0.689	0.878	0.536	-2.228
pqn-80	0.067	0.034	0.079	0.011	0.012	0.014	-2.533
pqn-82	0.113	0.123	0.111	0	0	0	-5.735
pqn-96	0.036	0.033	0.038	0.23	0.22	0.296	2.737
prg-1	0.022	0.023	0.021	0.133	0.156	0.134	2.474
T19D2.2	0	0	0	0.131	0.141	0.105	5.716
prx-5	0	0	0	0.13	0.14	0.105	5.716
ptr-5	0	0	0	0.05	0.055	0.055	5.974
ptr-20	0	0	0	0.098	0.096	0.095	6.384
puf-3	0.511	0.501	0.581	0.034	0.044	0.037	-4.005
puf-6	0.066	0.068	0.07	0	0	0	-5.547
rab-7	0.267	0.279	0.234	0	0	0	-6.311
rab-30	0.154	0.134	0.154	0	0	0	-5.735
rad-50	0.033	0.034	0.032	0	0	0	-5.547
ran-1	3.366	3.878	3.954	0.421	0.417	0.407	-3.386
rde-1	0.672	0.272	0.208	0	0	0	-8.236
rde-4	0	0	0	0.97	0.903	0.971	8.54
rfc-1	0.045	0.055	0.056	0	0	0	-5.735
rfc-3	0.144	0.111	0.151	0	0	0	-5.902

rfs-1	0.22	0.13	0.2	1.562	1.588	1.196	2.443
rha-1	0.166	0.112	0.08	0	0	0	-6.808
ric-3	0.443	0.331	0.122	0	0	0	-6.052
ric-19	0.211	0.233	0.25	0	0	0	-6.968
rpl-27	0.087	0.087	0.082	1.277	1.763	1.268	3.73
rpl-37	0.554	0.665	0.611	0	0	0	-6.187
rpn-7	0.067	0.076	0.051	0.333	0.543	0.308	2.421
rps-0	0.091	0.092	0.093	0.789	0.879	0.767	2.884
rps-17	0.488	0.477	0.408	0	0	0	-6.052
rpt-1	0.166	0.177	0.132	0	0	0	-6.187
rpt-2	0.079	0.068	0.09	0	0	0	-5.547
rpt-6	0.088	0.077	0.067	0.543	0.523	0.526	2.823
rsd-2	0.046	0.045	0.037	0	0	0	-5.735
rsp-1	0	0	0	0.12	0.13	0.101	5.716
rsp-2	0.134	0.143	0.142	0	0	0	-5.735
rsp-3	0.112	0.132	0.113	0	0	0	-5.735
rsp-6	0	0	0	0.392	0.323	0.399	6.963
san-1	0.764	0.764	0.466	0	0	0	-7.759
sax-2	0.046	0.035	0.065	0.007	0.003	0.007	-3.188
sca-1	0.067	0.078	0.057	0	0	0	-6.187
scp-1	0.022	0.023	0.022	0.456	0.356	0.573	4.537
sdc-1	0.057	0.068	0.071	0	0	0	-6.531
sdc-3	0	0	0	0.025	0.025	0.03	5.974
sec-5	0.011	0.013	0.013	0.313	0.123	0.127	3.024
sel-5	0.076	0.074	0.074	0	0	0	-6.531
sel-7	0.021	0.013	0.011	0.399	0.368	0.352	4.708
sel-11	0	0	0	0.381	0.311	0.328	7.765
44075	0.009	0.009	0.01	0.045	0.093	0.099	3.149
ser-1	0	0	0	0.068	0.079	0.079	5.716
ser-3	1.656	1.877	1.546	0.128	0.118	0.179	-3.214
seu-1	0	0	0	0.072	0.073	0.071	5.716
shw-3	0.233	0.443	0.299	0.014	0.032	0.02	-3.917
F56B3.4	0	0	0	0.164	0.139	0.131	6.384
skr-21	0	0	0	0.445	0.414	0.411	6.702
sli-1	0.113	0.11	0.163	0.012	0.013	0.015	-3.468
slo-2	0	0	0	0.056	0.052	0.053	5.974
sls-2.2	0	0	0	2.008	2.011	2.071	6.194
sls-2.3	0	0	0	5.65	5.696	5.621	7.622
smo-1	0.767	0.733	0.722	0	0	0	-6.89
smp-1	0.078	0.078	0.083	0	0	0	-6.187
snf-5	2.552	2.232	2.206	0.24	0.25	0.203	-3.545
snf-6	0.322	0.433	0.257	0.022	0.012	0.03	-3.139
snf-10	1.103	0.235	0.951	0	0	0	-9.368

snr-1	0.123	0.167	0.197	0	0	0	-5.547
snt-4	0.078	0.079	0.089	0	0	0	-5.547
spc-1	0.004	0.002	0.01	0.053	0.052	0.058	2.421
spe-5	0.014	0.041	0.012	0.138	0.148	0.182	3.638
spe-6	0.332	0.211	0.112	0	0	0	-5.547
spe-9	0.072	0.068	0.075	0.564	0.366	0.418	2.337
spe-15	0.056	0.044	0.045	0	0	0	-5.902
spe-26	0.323	0.602	0.403	0	0	0	-7.962
spe-29	5.899	5.551	5.128	1.034	1.046	1.05	-2.398
spg-7	0.099	0.086	0.088	0	0	0	-6.187
spo-11	0.222	0.256	0.228	0	0	0	-6.808
spp-8	0.173	0.133	0.178	0	0	0	-6.425
spp-12	0.302	0.122	0.328	0	0	0	-5.547
spr-3	0	0	0	0.194	0.138	0.104	6.384
spr-4	0.056	0.035	0.081	0.003	0.006	0.008	-3.39
sra-12	0.473	0.654	0.425	0	0	0	-7.241
srb-7	0.345	0.667	0.291	0	0	0	-6.722
srb-10	0	0	0	0.126	0.136	0.156	5.716
src-1	0.456	0.132	0.124	0	0	0	-6.808
src-2	0.133	0.123	0.101	0	0	0	-5.902
srd-66	0.234	0.343	0.137	0	0	0	-5.547
sre-1	0.223	0.132	0.125	0	0	0	-5.547
srg-2	0.115	0.123	0.174	0	0	0	-5.902
srg-9	0.223	0.272	0.226	0	0	0	-6.311
srg-34	0.906	0.998	0.6	0	0	0	-7.759
srg-65	0.405	0.556	0.541	0	0	0	-7.471
srg-66	0.606	0.787	0.556	0	0	0	-7.574
srh-74	0	0	0	0.215	0.245	0.291	6.552
srh-112	0.223	0.272	0.226	0	0	0	-6.311
srh-135	0.305	0.307	0.386	0	0	0	-7.111
srh-168	0	0	0	0.053	0.054	0.058	5.716
srh-169	0	0	0	0.242	0.222	0.229	6.194
srh-198	0.078	0.068	0.089	0	0	0	-6.052
srh-217	0.109	0.108	0.172	0	0	0	-5.902
srh-268	10.335	9.534	11.565	2.346	2.347	2.387	-2.39
srh-276	0.014	0.013	0.019	0.326	0.336	0.358	3.925
sri-12	0.437	0.456	0.474	0.123	0.135	0.125	-2.016
srj-16	0.122	0.177	0.13	0	0	0	-5.547
srj-24	0.032	0.031	0.033	0.28	0.28	0.28	2.888
srp-6	0	0	0	0.211	0.131	0.113	5.716
sars-2	0.322	0.222	0.179	0	0	0	-6.425
sars-1	0.021	0.032	0.012	0.222	0.232	0.217	3.925
sru-17	0.146	0.159	0.153	0	0	0	-5.735

srv-32	0	0	0	0.126	0.136	0.158	5.716
srw-3	0.656	0.333	0.127	0	0	0	-5.547
srw-4	0	0	0	0.172	0.124	0.177	5.974
srw-45	0	0	0	0.255	0.253	0.259	6.552
srw-62	0.156	0.133	0.185	0	0	0	-6.187
srw-103	0	0	0	0.21	0.208	0.206	6.194
srx-11	0.255	0.256	0.238	0	0	0	-6.187
srx-41	0	0	0	0.282	0.219	0.231	6.194
srx-125	0.133	0.133	0.172	0	0	0	-5.902
stc-1	1.445	1.667	1.006	0.244	0.455	0.232	-2.223
str-7	0.552	0.253	0.216	0	0	0	-6.808
str-9	0.563	0.432	0.317	0.057	0.068	0.06	-2.456
str-30	0.122	0.133	0.169	0	0	0	-5.902
str-33	0	0	0	0.496	0.457	0.439	7.184
str-67	1.968	0.963	1.12	0	0	0	-8.385
str-118	1.596	1.092	1.397	0	0	0	-9.015
str-139	0	0	0	0.215	0.218	0.219	6.194
str-141	0	0	0	0.269	0.267	0.259	6.384
str-205	0.224	0.553	0.128	0	0	0	-5.547
str-216	0.433	0.646	0.432	0	0	0	-7.111
str-227	0	0	0	0.125	0.145	0.154	5.716
sur-5	0	0	0	0.097	0.093	0.091	6.194
sur-6	0.013	0.014	0.015	0.442	0.432	0.167	3.263
sym-3	0.222	0.279	0.22	0	0	0	-6.531
syx-3	0.026	0.027	0.029	0.234	0.322	0.346	3.369
taf-1	0.057	0.067	0.023	0	0	0	-5.547
taf-9	0	0	0	0.98	1	0.978	7.544
tag-10	0.245	0.212	0.247	0	0	0	-7.041
nck-1	0.177	0.441	0.117	0	0	0	-6.052
sdhb-1	0	0	0	0.673	0.673	0.827	8.278
nrfl-1	0	0	0	0.085	0.081	0.083	5.974
ant-1.1	0.041	0.031	0.014	0.552	0.523	0.519	4.852
pak-2	0	0	0	0.085	0.082	0.087	5.716
tag-97	0.07	0.068	0.064	0	0	0	-5.547
fntb-1	0.672	0.716	0.367	0	0	0	-7.471
cpna-1	0.355	0.324	0.358	0.046	0.043	0.046	-3.061
tag-164	1.403	1.453	1.24	0	0	0	-8.619
tba-6	0.055	0.044	0.046	0.328	0.348	0.448	3.134
tba-8	0.122	0.233	0.111	0	0	0	-5.902
tbx-36	0.133	0.164	0.134	0	0	0	-5.735
tlf-1	0	0	0	0.122	0.116	0.152	6.552
tmd-2	0.026	0.025	0.023	0.232	0.322	0.168	2.713
tni-3	0.443	0.554	0.217	0	0	0	-6.187

tni-4	0.443	0.332	0.365	0	0	0	-6.722
tnt-3	0	0	0	0.042	0.041	0.043	5.716
top-3	0	0	0	0.128	0.184	0.122	6.552
tre-1	0.023	0.027	0.027	0.233	0.245	0.228	2.918
tre-4	0.044	0.046	0.049	0.223	0.223	0.25	2.209
tre-5	1.442	1.322	1.175	0.095	0.094	0.093	-3.758
try-5	10.55	10.336	10.028	2.622	2.634	2.646	-2.036
tsp-15	0.467	0.401	0.478	0.025	0.024	0.029	-4.023
twk-1	0	0	0	0.148	0.176	0.142	6.194
twk-9	0.073	0.035	0.088	0	0	0	-5.735
twk-42	0.122	0.122	0.181	0	0	0	-6.425
twk-45	0.323	0.211	0.328	0.079	0.079	0.072	-2.268
ubc-3	0.411	0.133	0.114	0	0	0	-5.735
ubc-19	0.322	0.332	0.186	0	0	0	-6.052
unc-3	0.056	0.065	0.046	0.234	0.245	0.207	2.004
unc-6	0.068	0.099	0.056	0	0	0	-5.547
unc-43	0.014	0.015	0.014	0.633	0.655	0.621	5.255
unc-57	0.066	0.045	0.078	0	0	0	-5.547
unc-61	0.034	0.035	0.058	0.565	0.615	0.51	2.996
unc-62	0.114	0.124	0.154	0.013	0.012	0.013	-3.611
unc-64	2.656	2.124	2.705	0.454	0.764	0.396	-2.879
unc-79	0.088	0.125	0.077	0	0	0	-7.417
unc-105	2.445	2.675	2.181	0.133	0.193	0.134	-4.132
unc-112	0	0	0	0.861	0.605	0.353	8.278
vab-10	0	0	0	0.012	0.031	0.011	5.716
vem-1	0	0	0	0.123	0.133	0.126	5.716
spon-1	0	0	0	0.124	0.144	0.152	6.963
ver-4	0	0	0	0.034	0.035	0.04	5.716
vha-7	0.055	0.077	0.034	0	0	0	-5.547
vars-2	0.063	0.069	0.069	0	0	0	-6.425
wah-1	0.012	0.012	0.016	0.111	0.122	0.134	2.888
wnk-1	0	0	0	0.024	0.025	0.025	5.716
wsp-1	0.066	0.068	0.067	0	0	0	-6.052
zak-1	0.032	0.014	0.016	0.273	0.283	0.228	3.648
zif-1	0.122	0.223	0.269	0.018	0.018	0.018	-3.861
zyg-1	0.122	0.103	0.105	0	0	0	-6.425
alx-1	0.044	0.074	0.074	0	0	0	-6.187
tag-191	0.224	0.343	0.338	0	0	0	-6.968
crn-7	0.244	0.322	0.218	0	0	0	-6.63
vps-52	0	0	0	0.189	0.122	0.129	6.552
acs-10	1.327	0.533	0.433	0	0	0	-8.036
AH10.2	0.222	0.243	0.206	0	0	0	-5.735
B0001.2	0.057	0.052	0.052	0	0	0	-5.735

B0024.15	1.427	2.098	1.454	0	0	0	-9.411
B0035.1	0.332	0.133	0.157	0	0	0	-6.052
pfd-4	0.79	0.879	0.903	0.235	0.246	0.238	-2.016
dhcr-7	0.667	0.869	0.689	0.123	0.124	0.131	-2.493
B0284.3	0.555	0.333	0.498	0	0	0	-6.722
B0334.10	0.233	0.323	0.277	0	0	0	-5.735
B0379.6	2.529	2.179	2.069	0	0	0	-8.173
B0391.10	0.233	0.153	0.279	0.042	0.052	0.018	-3.971
B0393.4	0.078	0.088	0.089	0	0	0	-6.311
B0393.7	0.789	0.545	0.756	0.155	0.133	0.111	-2.846
B0457.2	0.233	0.245	0.215	0	0	0	-6.425
lgc-20	0.133	0.322	0.113	0	0	0	-5.902
B0513.7	0.046	0.022	0.064	0.333	0.322	0.33	2.201
best-1	0.278	0.346	0.253	0	0	0	-7.041
C01B9.1	0.056	0.066	0.026	0	0	0	-5.547
cpna-3	0	0	0	0.153	0.132	0.119	6.194
C01G6.2	0	0	0	0.386	0.356	0.327	6.194
C01G6.3	0.132	0.132	0.119	0	0	0	-5.735
C01G6.5	0.013	0.014	0.01	0.078	0.082	0.08	2.737
C01G10.7	1.066	1.445	1.033	0.124	0.146	0.161	-2.781
C01G10.9	1.212	1.033	1.362	0.117	0.127	0.166	-3.129
C01H6.3	0.335	0.392	0.341	0	0	0	-6.89
C01H6.8	0.344	0.44	0.302	0	0	0	-6.425
C02F4.4	0.442	0.344	0.233	1.451	1.671	1.052	2.042
C03C10.7	44.756	44.888	41.763	12.282	10.218	11.285	-2.003
C03D6.1	0.332	0.334	0.218	0	0	0	-6.052
asfl-1	0.445	0.435	0.135	0	0	0	-5.547
C04F12.1	0.055	0.033	0.068	0	0	0	-6.187
rnh-1.3	0.322	0.333	0.369	0	0	0	-6.052
C04G2.10	0.144	0.133	0.167	0	0	0	-6.052
nlp-41	0	0	0	0.436	0.426	0.46	5.716
C05C10.7	0.654	0.666	0.617	0	0	0	-7.241
C05C12.1	0.337	0.323	0.107	0	0	0	-5.547
C05C12.5	0	0	0	0.24	0.22	0.295	5.716
C05D12.2	0.076	0.074	0.072	0	0	0	-6.311
C05G5.3	0	0	0	0.12	0.22	0.196	5.716
cdc-48.1	0	0	0	0.118	0.183	0.153	7.078
C06B3.1	0.557	0.888	0.572	0	0	0	-7.241
oac-3	0.221	0.278	0.248	0.035	0.037	0.031	-3.036
stdh-3	0.867	0.818	0.416	0	0	0	-7.361
C06C3.5	1.343	1.233	1.345	0.332	0.346	0.328	-2.136
C06C6.8	0.455	0.566	0.356	0	0	0	-5.735
atp-5	0	0	0	0.619	0.694	0.664	7.622

lmtr-3	0	0	0	0.546	0.558	0.505	6.702
C06H2.7	0.234	0.322	0.249	0	0	0	-6.808
tiar-3	0	0	0	0.092	0.093	0.096	5.716
C07A9.2	0.267	0.776	0.233	0	0	0	-6.311
stip-1	0.043	0.066	0.057	0	0	0	-5.735
C07E3.3	0.045	0.033	0.041	0	0	0	-5.735
C08B6.3	0.711	0.749	0.297	0	0	0	-7.361
C08B6.6	0	0	0	0.615	0.153	0.676	7.283
C08B11.8	0.144	0.189	0.174	0	0	0	-6.531
C08E8.2	0	0	0	0.126	0.124	0.16	5.716
C08F8.2	0.765	0.458	0.434	0.036	0.065	0.066	-2.803
fbxa-98	0.167	0.178	0.176	0	0	0	-6.052
ugt-22	0	0	0	0.098	0.095	0.095	5.974
glb-4	2.33	2.78	2.004	0.175	0.158	0.153	-3.801
C09H10.9	1.949	1.24	1.99	0.346	0.346	0.392	-2.45
cbl-1	0.124	0.133	0.158	0	0	0	-6.425
B0334.12	2.436	2.456	2.176	0	0	0	-5.735
nhr-154	0	0	0	0.121	0.131	0.114	5.716
spin-1	0.441	0.554	0.112	0	0	0	-6.187
C14B1.8	0.333	0.378	0.328	0	0	0	-6.311
C14B4.2	0.055	0.055	0.036	0	0	0	-6.052
C14H10.3	0.023	0.055	0.044	0	0	0	-5.547
xbx-9	0.334	0.234	0.379	0.042	0.052	0.022	-4.074
C15C8.4	0.166	0.188	0.103	0.566	0.676	0.603	2.411
C17E4.4	0.345	0.567	0.511	0.061	0.079	0.067	-2.925
C17G1.5	0.123	0.113	0.149	0	0	0	-6.052
maa-1	0.278	0.289	0.269	0	0	0	-6.311
tomm-40	0.541	0.341	0.101	0	0	0	-5.547
C25A1.1	0.157	0.333	0.13	0	0	0	-5.547
clec-87	0.457	0.445	0.49	0.069	0.079	0.087	-2.576
C25A1.15	0.111	0.112	0.138	1.123	1.134	1.179	2.888
C25F9.2	0.333	0.374	0.325	0	0	0	-9.015
C25F9.4	0.063	0.064	0.067	0	0	0	-5.547
C26E1.2	0.124	0.145	0.192	0	0	0	-6.63
ceh-79	0.035	0.057	0.053	0.223	0.225	0.29	2.302
C27B7.2	0.237	0.227	0.268	1.998	1.785	1.785	2.603
tofu-1	0.334	0.133	0.172	0	0	0	-6.311
C28D4.10	0.511	0.532	0.554	0	0	0	-6.187
C31H5.7	0	0	0	0.402	0.423	0.444	5.974
C33A12.1	0.035	0.036	0.033	0.91	0.912	0.954	4.549
nlp-35	0.272	0.227	0.275	4.568	4.345	4.772	3.97
C33A12.19	2.441	2.433	2.113	9.122	9.244	9.208	2.007
C34C12.7	0.443	0.553	0.23	0	0	0	-6.187

C34C12.9	0.239	0.663	0.29	0	0	0	-5.547
C34D1.4	0	0	0	0.156	0.124	0.158	5.716
zip-5	0.433	0.489	0.466	0	0	0	-6.808
C34F6.1	0	0	0	0.091	0.099	0.094	6.702
C34F6.9	0.045	0.067	0.076	0	0	0	-6.722
C35A5.4	0.99	0.558	0.45	0	0	0	-7.759
C35A5.5	0	0	0	0.104	0.101	0.112	5.974
hda-11	0	0	0	0.223	0.229	0.231	6.384
irx-1	0	0	0	0.185	0.15	0.159	6.552
C38C10.6	5.422	4.999	2.017	0	0	0	-7.802
C38D4.7	0.332	0.232	0.182	0	0	0	-5.902
fbxa-172	0	0	0	0.153	0.129	0.175	6.552
C39E9.8	0.143	0.156	0.134	0	0	0	-5.547
ctns-1	0.433	0.411	0.472	0.345	0.234	0.119	-2.086
vesa-1	0.877	0.7	0.677	0.045	0.055	0.051	-3.814
C44B9.2	1.631	1.434	1.41	0	0	0	-9.137
C44C10.5	0	0	0	0.155	0.124	0.177	5.716
lron-1	0.44	0.45	0.24	0	0	0	-7.361
C44H4.4	0.767	0.811	0.838	0.022	0.024	0.02	-5.425
C46F11.6	0.293	0.223	0.292	0	0	0	-6.425
C47A10.12	2.455	2.765	2.049	0.166	0.117	0.17	-3.669
sfxn-1.4	8.113	8.834	8.862	1.668	1.628	1.68	-2.512
C47E12.3	0	0	0	0.268	0.682	0.253	7.283
pyp-1	0.082	0.083	0.083	0.724	0.777	0.752	3.033
acox-5	0	0	0	0.106	0.156	0.109	6.194
rin-1	0	0	0	0.039	0.032	0.037	6.194
ceh-88	0.046	0.043	0.069	0.323	0.351	0.352	2.187
C49C3.7	0.019	0.013	0.019	0.238	0.255	0.266	3.47
C49C3.10	0.433	0.564	0.234	0	0	0	-6.722
set-6	0.045	0.046	0.063	0.312	0.345	0.338	2.28
C52A11.3	0.223	0.234	0.288	0	0	0	-6.425
mans-2	0.074	0.066	0.07	0	0	0	-5.735
C52E4.7	0.057	0.077	0.078	0.356	0.358	0.398	2.201
srt-32	0.145	0.132	0.15	0	0	0	-5.735
C53B4.4	0.004	0.006	0.008	0.079	0.08	0.056	2.569
dct-3	0.289	0.342	0.288	0	0	0	-7.177
C54D10.8	0.611	0.912	0.672	0	0	0	-8.173
C54D10.10	0.223	0.453	0.364	0.042	0.041	0.048	-2.925
C54G10.4	0.031	0.056	0.07	0.333	0.321	0.361	2.215
tpra-1	0.074	0.045	0.073	0	0	0	-5.547
D1054.3	0.663	0.445	0.314	0	0	0	-6.187
cdc-5L	0	0	0	0.07	0.073	0.078	5.974
D2005.3	10.119	10.557	10.95	1.232	1.242	1.222	-3.272

D2013.3	0.188	0.156	0.18	0	0	0	-6.311
D2023.4	0.092	0.093	0.096	0.532	0.523	0.547	2.337
mce-1	0	0	0	0.376	0.306	0.308	6.384
D2030.8	0.046	0.048	0.047	0.211	0.224	0.208	2.004
wdr-23	0.222	0.242	0.216	1.909	1.911	1.939	3.045
43892	1.786	1.345	1.38	0.243	0.245	0.21	-2.804
D2089.3	23.444	23.355	24.38	5.234	5.567	5.651	-2.224
DH11.2	0.031	0.041	0.012	0.139	0.129	0.192	3.638
DY3.8	0.133	0.145	0.176	0	0	0	-5.547
E01G6.3	0	0	0	0.177	0.11	0.133	6.384
E02H1.5	0.145	0.154	0.159	0	0	0	-5.547
nhr-174	0	0	0	0.203	0.243	0.205	6.194
ugt-44	0.033	0.033	0.032	0.489	0.479	0.491	3.762
F01D4.3	0.033	0.045	0.086	0	0	0	-5.735
lipl-7	0.177	0.128	0.129	0	0	0	-5.902
F01G10.9	0.212	0.322	0.116	0	0	0	-6.052
F02D10.3	0.022	0.015	0.018	0.31	0.303	0.377	4.049
F02E9.5	0.357	0.344	0.344	0	0	0	-6.531
scav-6	0.442	0.323	0.157	0	0	0	-6.63
kcnl-2	0.056	0.047	0.048	0	0	0	-5.547
prmn-1	0.013	0.016	0.013	0.093	0.092	0.099	2.737
F08G2.5	0.147	0.157	0.171	1.715	1.723	1.755	3.215
F08G2.7	0	0	0	0.135	0.175	0.125	5.974
dsb-1	0.182	0.101	0.184	0	0	0	-6.425
acl-13	0.333	0.963	0.54	0	0	0	-7.715
F08G12.1	0.11	0.122	0.197	0.056	0.045	0.046	-2.18
F08H9.2	0.566	0.445	0.262	0	0	0	-5.547
F09A5.4	0.044	0.042	0.043	0.607	0.67	0.603	3.653
F09B9.5	0.998	0.689	0.873	0.126	0.116	0.16	-2.526
F09E8.8	1.122	1.322	1.195	0	0	0	-6.63
pch-2	0.643	0.789	0.607	0.123	0.231	0.142	-2.198
F10D11.6	0	0	0	0.063	0.069	0.068	5.974
lips-3	0.523	0.511	0.543	0.051	0.052	0.053	-3.409
lron-5	0.222	0.89	0.499	0	0	0	-8.467
clec-154	1.189	1.289	0.892	0	0	0	-9.033
clec-152	0.453	0.433	0.202	0	0	0	-6.89
clec-151	0.094	0.091	0.092	0	0	0	-5.902
clec-153	0.318	0.599	0.416	0	0	0	-7.999
ercc-1	0.278	0.428	0.205	0	0	0	-6.311
F10G8.9	0.881	0.76	0.561	0	0	0	-7.999
F11A1.2	1.334	1.456	1.644	0.043	0.042	0.049	-5.045
acs-14	0	0	0	0.199	0.17	0.111	5.974
F11A3.2	1.221	1.441	1.102	5.512	5.524	5.588	2.226

gba-3	0.222	0.277	0.231	0	0	0	-7.177
F11E6.9	0	0	0	1.461	1.464	1.44	8.179
gnrr-6	0.747	0.747	0.747	0	0	0	-8.413
F13E9.11	2.455	2.877	2.068	0.322	0.311	0.31	-2.845
ztf-2	0.211	0.322	0.109	0	0	0	-6.052
F13G3.12	0.897	0.565	0.334	0.032	0.031	0.034	-3.309
F13H10.5	0.123	0.133	0.124	0	0	0	-5.902
rpoa-2	0	0	0	0.058	0.054	0.052	5.974
git-1	0.145	0.168	0.148	0.922	0.912	0.944	2.544
cyp-13A11	0.322	0.433	0.123	0	0	0	-6.052
shw-1	0.056	0.078	0.091	0	0	0	-6.052
fbxa-100	0	0	0	0.121	0.139	0.188	5.974
F15G9.1	0.123	0.178	0.124	0	0	0	-5.547
F15G9.5	0.494	0.545	0.359	0	0	0	-6.722
ppfr-1	0.055	0.043	0.046	0.222	0.223	0.237	2.23
F16B12.4	0	0	0	0.304	0.338	0.34	7.078
F16B12.6	0.079	0.099	0.06	0	0	0	-6.531
ser-5	3.678	4.041	3.931	0	0	0	-10.999
vps-36	0	0	0	0.134	0.139	0.139	6.194
F17H10.1	0.062	0.064	0.062	0.322	0.334	0.301	2.133
F17H10.4	0	0	0	0.439	0.474	0.472	5.974
srz-33	0.152	0.134	0.157	0	0	0	-5.735
F19B2.5	0	0	0	0.319	0.243	0.219	5.716
F19C6.3	0	0	0	0.321	0.307	0.353	7.184
nekl-3	0.031	0.032	0.035	0.443	0.333	0.301	2.888
mltn-10	0.056	0.045	0.019	0.134	0.124	0.132	2.569
F20G2.1	0.032	0.032	0.023	0.38	0.36	0.398	3.789
glb-14	1.236	1.545	1.58	0.128	0.113	0.183	-3.193
arrd-28	1.454	1.766	1.172	0.065	0.063	0.065	-4.263
F21D5.4	0.738	0.737	0.647	0	0	0	-7.999
F21G4.1	3.145	3.422	3.449	0.709	0.723	0.739	-2.336
F22B8.4	0.015	0.014	0.016	0.258	0.258	0.278	3.789
F22D6.2	0.043	0.035	0.033	1.713	1.988	1.769	5.531
nduf-6	0.441	0.561	0.116	0.512	0.524	0.595	2.201
ekl-1	0.007	0.006	0.009	0.155	0.175	0.151	3.789
chw-1	0.076	0.077	0.071	0.609	0.678	0.692	3.097
F22G12.3	0.122	0.178	0.165	0	0	0	-5.902
fipr-1	0.979	0.779	0.785	0	0	0	-6.722
pyk-1	0.082	0.084	0.085	0.456	0.678	0.563	2.593
mrpl-54	0.052	0.052	0.052	0.439	0.429	0.488	3.024
F25H8.2	0.145	0.178	0.135	0	0	0	-6.052
lurp-2	0.188	0.133	0.123	0	0	0	-5.547
F25H9.2	0.178	0.148	0.157	0	0	0	-6.052

lurp-3	0.122	0.132	0.144	0	0	0	-5.735
bath-39	0.442	0.353	0.235	0	0	0	-6.722
F25H9.7	0.781	0.978	0.772	0	0	0	-7.111
F26A3.4	0.013	0.012	0.019	0.554	0.675	0.512	4.462
gpx-1	0.334	0.455	0.302	0	0	0	-5.902
F26H9.5	0.043	0.042	0.045	1.432	1.452	1.175	4.528
nurf-1	0.222	0.123	0.123	0.823	0.822	0.826	2.166
aptf-3	0.277	0.349	0.201	0	0	0	-6.968
acs-20	0.541	0.541	0.108	0	0	0	-6.531
rict-1	0.056	0.068	0.034	0	0	0	-5.902
F29G6.1	0.067	0.088	0.052	0	0	0	-6.052
F29G6.2	0.063	0.061	0.068	0.444	0.345	0.376	2.315
F30A10.3	1.259	0.697	0.598	0	0	0	-8.173
F31B12.4	0.332	0.366	0.337	0	0	0	-5.735
F31C3.3	0.033	0.038	0.033	0	0	0	-6.311
F31F6.2	0	0	0	0.173	0.137	0.131	5.974
maph-1.1	3.145	2.092	2.197	0.435	0.355	0.346	-2.777
F32G8.2	0.014	0.015	0.011	0.127	0.137	0.172	3.638
F32H2.7	0.331	0.144	0.101	0	0	0	-5.547
F32H5.1	0.014	0.015	0.016	0.323	0.317	0.374	4.27
F33A8.7	2.565	2.15	2.05	0	0	0	-8.901
F33E2.6	0.054	0.044	0.062	0	0	0	-5.902
glb-15	1.215	1.145	1.154	5.71	5.724	5.799	2.211
F35B12.10	0	0	0	0.108	0.182	0.152	6.194
clec-62	0.221	0.299	0.257	0	0	0	-6.808
F35C11.6	0.032	0.043	0.024	0.678	0.71	0.79	4.708
F35G2.1	0.346	0.672	0.151	0	0	0	-7.111
mlcd-1	0.326	0.562	0.271	0	0	0	-7.111
F35G12.5	0.555	0.445	0.165	0	0	0	-6.187
F35G12.11	0.877	0.977	0.706	0.112	0.109	0.115	-2.676
F35H8.4	1.567	1.546	1.313	0	0	0	-7.177
F37H8.3	0.322	0.311	0.307	0	0	0	-6.425
F38A1.11	0.334	0.135	0.614	0	0	0	-7.574
ccch-1	0	0	0	0.191	0.129	0.183	7.184
F38H4.5	2.453	2.773	2.314	0.236	0.226	0.261	-3.244
mig-38	0.07	0.08	0.099	1.489	1.487	1.47	3.765
F41D3.11	0	0	0	0.231	0.207	0.273	6.838
F41E7.7	0	0	0	0.488	0.425	0.488	5.974
F42A8.1	0.043	0.042	0.044	0.441	0.422	0.449	3.179
F42E11.3	0.444	0.544	0.368	0	0	0	-6.63
F42F12.3	0	0	0	0.284	0.228	0.22	5.974
F42G10.1	0	0	0	0.175	0.146	0.127	6.552
lmd-1	0.867	0.789	0.702	0.112	0.124	0.141	-2.401

F43G9.13	0.385	0.322	0.389	0	0	0	-6.052
nucb-1	0	0	0	0.128	0.109	0.117	5.974
F44A6.5	0	0	0	0.2	0.207	0.2	5.974
ent-7	0.045	0.121	0.091	0	0	0	-5.735
F44G4.3	0.673	0.356	0.307	0	0	0	-5.735
dep-1	0.008	0.009	0.009	0.173	0.183	0.131	3.73
F46A8.5	0.133	0.134	0.193	0	0	0	-5.547
F46A8.7	0.464	0.564	0.641	6.224	6.344	6.387	3.192
lipl-2	0	0	0	0.287	0.271	0.229	6.702
F46C5.1	2.474	2.754	2.739	18.117	17.612	16.69	2.49
F46G10.2	0.013	0.014	0.017	0.134	0.125	0.173	3.149
F46G10.4	0.255	0.442	0.21	0	0	0	-6.808
ipla-2	0	0	0	0.045	0.042	0.045	5.716
F47B10.3	0	0	0	0.229	0.22	0.228	6.384
lsy-27	0	0	0	0.246	0.246	0.252	6.384
F48F5.2	0.632	0.566	0.156	0	0	0	-7.884
F49B2.6	0.233	0.543	0.48	0.043	0.042	0.048	-3.404
F49C12.5	0.133	0.544	0.172	0	0	0	-6.531
F49C12.10	3.123	3.1	3.51	0.455	0.765	0.503	-2.909
F49C12.11	0.123	0.145	0.198	1.477	1.587	1.691	2.933
skpo-1	0.123	0.122	0.118	0	0	0	-6.425
F49E12.7	0.566	0.755	0.547	0	0	0	-6.968
F49E12.12	0	0	0	0.227	0.273	0.273	6.552
F49H6.8	0	0	0	1.439	1.409	1.439	7.078
F53A2.9	0	0	0	0.158	0.18	0.153	5.974
F53B6.5	0.333	0.554	0.381	0	0	0	-6.187
F53B6.7	0.213	0.234	0.166	0	0	0	-5.902
F53C11.1	0.078	0.098	0.085	0	0	0	-5.547
swan-2	0.033	0.032	0.035	0.677	0.577	0.388	3.31
cutl-1	0	0	0	0.134	0.125	0.136	5.716
F53F1.4	0	0	0	0.322	0.332	0.308	5.716
F53F1.6	0	0	0	0.124	0.123	0.164	5.716
F53F4.4	0	0	0	0.086	0.086	0.089	5.974
F53F4.13	0	0	0	0.4	0.43	0.434	5.974
F53F4.15	0.458	0.433	0.484	0	0	0	-5.902
F54B3.2	0	0	0	0.143	0.112	0.147	5.716
nhr-282	0	0	0	0.344	0.34	0.384	6.552
F54B11.11	0.144	0.126	0.125	0	0	0	-6.311
F54C8.1	10.006	8.343	10.665	0.418	0.413	0.481	-4.577
F54D5.11	2.222	2.662	2.233	0.765	0.456	0.494	-2.285
F54D5.15	0.133	0.133	0.105	0	0	0	-6.052
F54F11.1	0	0	0	0.124	0.115	0.178	5.716
nep-17	0	0	0	0.055	0.051	0.06	6.552

sre-45	0.438	0.658	0.377	0.026	0.022	0.029	-3.678
F54F12.2	0.133	0.155	0.136	0.456	0.486	0.64	2.097
F55A11.11	0.278	0.322	0.229	0	0	0	-6.311
F55H2.7	0.432	0.442	0.417	3.722	3.743	3.703	3.03
F55H12.3	0	0	0	0.033	0.038	0.036	6.702
F56A8.3	0.136	0.145	0.111	0	0	0	-6.052
F56A8.8	0.322	0.326	0.324	0	0	0	-6.425
F56D5.3	0.145	0.132	0.113	0	0	0	-5.735
F56D5.5	1.785	0.777	0.728	0	0	0	-7.802
F56G4.6	0.132	0.189	0.124	0	0	0	-6.808
F56H11.6	0.146	0.189	0.157	0	0	0	-5.735
F57A8.1	1.551	1.441	1.057	9.99	9.87	9.014	2.976
yif-1	0.134	0.124	0.139	1.105	1.205	1.048	2.786
F57A10.2	0.424	0.465	0.444	0	0	0	-6.425
F57C2.4	0.214	0.559	0.934	0	0	0	-6.722
F58A3.3	0.067	0.065	0.083	0.55	0.455	0.498	2.421
F58B3.4	0.013	0.01	0.01	0.125	0.213	0.139	3.47
F58B3.7	0.033	0.03	0.03	0.678	0.7	0.676	4.258
F58B4.5	0	0	0	0.192	0.118	0.162	6.194
F58D5.2	0.052	0.053	0.057	0.534	0.524	0.538	3.024
F58E6.11	3.374	3.724	3.745	32.923	31.229	33.924	3.063
droe-4	2.555	2.785	2.48	0.533	0.523	0.593	-2.172
F58G11.4	0.233	0.542	0.204	0	0	0	-6.63
F58H1.2	23.333	21.783	22.32	3.144	3.771	3.136	-2.945
clpf-1	0.124	0.156	0.139	0	0	0	-6.052
F59B10.3	0.061	0.061	0.062	0.454	0.345	0.353	2.337
glo-3	0.066	0.056	0.066	0	0	0	-5.547
H02I12.5	0.332	0.332	0.214	0	0	0	-6.531
enu-3.1	0	0	0	0.114	0.115	0.161	5.716
H04D03.4	0.045	0.078	0.091	0	0	0	-6.187
H06A10.1	0	0	0	0.196	0.116	0.194	6.384
H06O01.4	0.788	0.978	0.709	0.198	0.158	0.115	-2.676
H08M01.1	0.897	0.833	0.549	0	0	0	-7.715
H12D21.10	0	0	0	0.113	0.137	0.115	5.974
H21P03.2	0.079	0.083	0.091	0	0	0	-5.547
mcrs-1	0.442	0.322	0.218	0.056	0.046	0.041	-2.456
hpo-11	0.048	0.056	0.068	0	0	0	-5.547
H38K22.4	1.455	1.325	1.518	8.916	8.01	8.962	2.431
H39E23.2	0	0	0	0.369	0.394	0.33	6.194
magi-1	0	0	0	0.057	0.053	0.057	5.974
K01A6.6	0.254	0.178	0.112	0	0	0	-5.902
K01A11.2	0.237	0.246	0.244	0	0	0	-6.052
K01B6.4	1.861	1.893	1.679	0	0	0	-7.962

mrpl-10	0.051	0.051	0.05	0.434	0.344	0.431	2.888
hrg-2	0.164	0.277	0.145	0	0	0	-5.547
meg-2	0.034	0.036	0.04	0.177	0.155	0.185	2.046
K02B12.6	0.256	0.356	0.258	0	0	0	-5.547
K03B8.6	0.144	0.177	0.142	0	0	0	-6.187
mys-2	0	0	0	0.073	0.082	0.099	5.974
smg-8	0	0	0	0.025	0.084	0.084	6.194
vacl-14	0.046	0.068	0.059	0	0	0	-5.547
K04G2.7	0.322	0.345	0.336	0.034	0.033	0.032	-3.39
K05C4.10	0.452	0.412	0.456	0	0	0	-6.425
sol-2	0.078	0.057	0.09	0	0	0	-5.547
swt-5	0.059	0.086	0.089	0.628	0.788	0.833	3.07
K07A1.9	0.167	0.188	0.132	0	0	0	-6.187
K07A1.13	0.345	0.565	0.887	0.055	0.055	0.047	-4.215
K07A12.1	0.079	0.089	0.075	0	0	0	-5.735
K07G5.4	0.124	0.111	0.126	0	0	0	-5.547
fecl-1	0	0	0	0.112	0.115	0.126	5.716
K08C7.6	0.023	0.024	0.02	0.318	0.328	0.379	3.925
K08E4.2	1.448	1.998	1.792	0.434	0.324	0.369	-2.384
K08E7.4	0.377	0.378	0.373	0	0	0	-5.547
K08E7.5	0.033	0.03	0.034	0	0	0	-5.547
K08F8.7	0.145	0.129	0.195	0	0	0	-5.547
uda-1	0.083	0.078	0.081	0.262	0.622	0.624	2.805
trpp-6	0.054	0.052	0.054	0.336	0.534	0.559	3.149
nipi-3	0	0	0	0.249	0.286	0.205	7.283
uso-1	0.006	0.006	0.007	0.12	0.13	0.104	3.638
K09E4.1	1.563	1.453	1.223	0.123	0.189	0.191	-2.839
K10C3.5	0.012	0.012	0.013	0.132	0.122	0.125	3.024
frpr-15	0.321	0.332	0.203	0.021	0.022	0.023	-3.13
K10H10.7	0	0	0	0.439	0.487	0.458	6.194
K11D2.4	0.044	0.047	0.041	0.235	0.223	0.284	2.598
twk-48	1.756	1.346	1.629	0.325	0.335	0.348	-2.334
K12D12.4	0.183	0.189	0.16	0	0	0	-5.902
sodh-2	0.131	0.121	0.107	1.334	1.544	1.315	3.467
M01A8.1	0.11	0.123	0.177	0	0	0	-5.902
sepa-1	0	0	0	0.035	0.036	0.074	5.716
M01F1.4	0.049	0.041	0.05	0	0	0	-5.547
M01G12.14	0	0	0	0.984	0.942	0.953	8.371
chl-1	0.223	0.155	0.124	0.023	0.025	0.025	-2.374
M03C11.6	0.263	0.256	0.203	0	0	0	-6.052
M04B2.2	0.432	0.231	0.231	0	0	0	-6.63
M04B2.4	0	0	0	0.137	0.137	0.13	6.194
M05D6.2	0.072	0.077	0.097	0	0	0	-5.902

M05D6.9	1.453	1.789	1.456	0	0	0	-8.799
dhhc-6	0	0	0	0.351	0.341	0.112	5.716
lact-3	0	0	0	0.249	0.29	0.255	7.078
M117.4	0.056	0.081	0.082	0	0	0	-5.902
rle-1	0.126	0.127	0.132	0.031	0.013	0.011	-3.611
M153.3	0.411	0.872	0.418	0	0	0	-6.808
M163.1	0	0	0	0.482	0.42	0.478	7.462
nduo-1	0.134	0.127	0.174	0	0	0	-5.735
ctc-2	0	0	0	0.449	0.494	0.465	6.702
R01H10.7	0.062	0.068	0.063	0	0	0	-5.902
metr-1	0.043	0.055	0.039	0	0	0	-5.735
ugt-47	0.034	0.034	0.035	0.544	0.654	0.433	3.467
R05D11.4	0.056	0.057	0.06	0	0	0	-5.547
R05H5.3	0.922	0.789	0.946	0.132	0.142	0.118	-3.074
rbm-28	0	0	0	0.077	0.074	0.076	5.716
wago-1	0	0	0	0.054	0.037	0.053	5.716
R06C7.2	0.11	0.111	0.198	0	0	0	-6.425
vps-11	0.055	0.058	0.054	0	0	0	-5.902
R07B1.9	0.332	0.554	0.498	0.013	0.014	0.012	-5.341
R07E3.7	0	0	0	0.378	0.384	0.338	6.963
R07H5.9	0.232	0.276	0.204	0	0	0	-6.052
R09H10.2	0	0	0	0.218	0.219	0.228	6.194
swt-6	2.439	2.967	2.943	0.411	0.414	0.472	-2.749
R10E4.9	0.11	0.178	0.196	0	0	0	-5.547
R10E8.8	0.243	0.202	0.209	0	0	0	-6.311
usp-46	0.088	0.09	0.082	0	0	0	-5.547
nud-2	1.228	0.89	0.844	0	0	0	-8.44
R11A8.2	0	0	0	0.137	0.134	0.106	5.716
mpz-5	1.239	0.856	1.858	0	0	0	-7.999
pho-8	0.104	0.099	0.187	0	0	0	-6.311
R107.5	0	0	0	0.313	0.325	0.361	6.838
mnk-1	0.031	0.031	0.031	0.478	0.488	0.486	3.817
cdt-2	0.102	0.122	0.108	0	0	0	-6.425
T01E8.1	0	0	0	0.315	0.35	0.332	7.462
ubxn-5	0.389	0.356	0.365	0	0	0	-6.187
T01G1.2	1.777	2.128	1.834	0	0	0	-9.015
sec-31	0	0	0	0.043	0.043	0.045	5.716
dma-1	1.873	1.341	1.776	0	0	0	-10.294
perm-1	0	0	0	0.116	0.163	0.133	6.194
T01H3.5	0.367	0.378	0.339	0	0	0	-6.052
T02E1.2	0.095	0.092	0.094	0	0	0	-6.052
gla-3	1.123	1.234	1.849	0.138	0.138	0.184	-3.432
T03D8.6	0.344	0.366	0.292	0.065	0.062	0.062	-2.314

T03F7.7	0.432	0.432	0.295	0	0	0	-7.177
T04C12.3	0	0	0	0.412	0.415	0.411	6.194
T04D3.5	0.392	0.492	0.293	0	0	0	-6.968
T05A6.5	0.653	0.455	0.289	0.044	0.047	0.041	-2.867
ttr-14	0.447	0.567	0.666	3.222	3.233	3.206	2.149
decr-1.3	0.205	0.203	0.234	0	0	0	-6.311
enpl-1	0.123	0.108	0.132	0	0	0	-6.89
imp-2	0.327	0.313	0.368	1.633	1.645	1.615	2.014
rmd-1	5.128	5.038	5.847	1.036	1.016	1.057	-2.579
gcc-2	0.123	0.167	0.168	0.032	0.032	0.03	-2.533
gpcp-2	0.346	0.379	0.566	0.048	0.043	0.05	-3.595
T05H10.8	0	0	0	0.143	0.123	0.143	5.716
cgt-1	0	0	0	0.121	0.132	0.105	5.716
cox-15	0.166	0.166	0.127	0	0	0	-5.902
acl-2	0.245	0.246	0.249	0	0	0	-6.531
T06G6.8	0.122	0.121	0.14	0	0	0	-6.052
jmjd-4	0	0	0	0.597	0.569	0.58	7.765
mam-5	0.167	0.178	0.112	0	0	0	-6.187
T08D2.1	0	0	0	0.657	0.674	0.646	6.702
T08D2.7	0.117	0.199	0.162	0	0	0	-6.311
T08G5.7	0.051	0.051	0.054	0.457	0.477	0.458	2.888
ztf-17	0.094	0.093	0.09	0	0	0	-6.425
T09A5.14	1.011	1.077	1.025	0	0	0	-6.311
slc-17.9	0.031	0.021	0.01	0.109	0.124	0.188	3.925
T09B9.3	0.053	0.056	0.053	0.453	0.353	0.303	2.337
T09B9.5	0.166	0.177	0.154	0	0	0	-5.902
T10B10.3	0.063	0.066	0.068	0	0	0	-6.052
T10G3.1	0.122	0.188	0.196	0.023	0.023	0.026	-2.925
srab-19	0	0	0	0.124	0.124	0.166	5.716
T12A7.2	0	0	0	0.122	0.132	0.105	5.716
T12A7.7	0.278	0.256	0.296	0	0	0	-6.187
T13F2.6	0.106	0.106	0.106	0	0	0	-6.425
T13F3.6	0.445	0.456	0.478	0	0	0	-6.722
oac-46	0.189	0.192	0.174	0	0	0	-7.177
T14G8.4	1.568	1.788	1.527	0.125	0.215	0.151	-3.436
T15H9.6	0	0	0	0.244	0.24	0.244	7.184
T16G12.3	0	0	0	0.123	0.321	0.105	5.716
T16G12.9	0.167	0.189	0.152	0	0	0	-5.902
T16H12.1	1.334	1.234	1.07	0.322	0.342	0.248	-2.211
T16H12.2	2.189	2.009	2.057	0.053	0.052	0.06	-5.095
T18D3.7	0	0	0	0.269	0.289	0.205	6.194
T19A6.1	0	0	0	0.583	0.683	0.616	8.415
cyp-29A2	0.09	0.1	0.077	0	0	0	-5.547

T20B3.1	0.433	0.243	0.317	1.89	1.989	1.742	2.337
clec-183	0	0	0	0.222	0.344	0.25	5.716
T20D3.5	0.232	0.553	0.255	0	0	0	-6.722
T20D3.8	0.178	0.134	0.148	0	0	0	-5.547
T20G5.12	0	0	0	0.899	0.888	0.9	7.544
del-6	5.1	5.565	5.183	0.533	0.553	0.526	-3.412
T21C9.13	0.882	0.552	0.189	0	0	0	-6.311
hsp-12.1	0	0	0	0.818	0.879	0.812	6.838
T22B3.3	0.3	0.267	0.284	0	0	0	-5.902
T22C1.6	0.342	0.445	0.15	0	0	0	-6.311
T22C1.11	1.255	1.786	1.265	0.161	0.131	0.111	-3.594
scrm-1	0	0	0	0.119	0.119	0.184	6.194
pgrn-1	0.133	0.166	0.128	0	0	0	-5.735
T23B5.4	0.456	0.566	0.423	0	0	0	-6.052
ppm-2	0.092	0.094	0.092	0	0	0	-5.902
T23F11.4	0	0	0	0.132	0.145	0.123	5.716
saeg-2	0.177	0.179	0.105	0	0	0	-5.547
lron-9	4.235	4.345	4.598	1.344	1.044	1.096	-2.182
T23G11.7	0.144	0.179	0.111	0	0	0	-5.547
T24D1.2	0.034	0.067	0.096	0	0	0	-5.735
rsf-1	0.092	0.096	0.095	0	0	0	-6.052
inpp-1	2.266	2.111	2.033	0.016	0.022	0.019	-6.725
dkf-2	0	0	0	0.054	0.052	0.043	5.716
T26C5.2	0	0	0	0.119	0.147	0.114	6.194
T26G10.1	0.144	0.176	0.182	0	0	0	-6.63
T27F6.7	0.042	0.043	0.04	0.433	0.422	0.415	3.149
del-4	0.1	0.084	0.082	0	0	0	-5.547
T28B11.1	0.044	0.034	0.014	0.068	0.09	0.094	2.569
T28C6.5	0.177	0.145	0.134	0	0	0	-5.547
T28C6.8	22.224	21.323	20.36	3.831	3.821	3.809	-2.532
T28D6.5	0.922	0.978	0.919	0.11	0.122	0.154	-2.686
asic-2	0.333	0.441	0.314	0.057	0.053	0.06	-2.474
T28F4.3	0	0	0	0.244	0.243	0.204	6.702
inos-1	0.533	0.522	0.983	0	0	0	-8.267
VF13D12L.3	0.345	0.655	0.499	0.032	0.022	0.024	-4.384
W01A8.2	1.665	1.545	1.355	0.433	0.452	0.24	-2.595
W01B6.2	0	0	0	0.223	0.229	0.231	6.384
W01D2.3	0.663	0.883	0.934	0.146	0.126	0.192	-2.382
W01F3.2	0.346	0.388	0.364	0	0	0	-7.041
mlt-11	0.066	0.047	0.037	0	0	0	-6.968
W02A11.1	0.145	0.124	0.129	0	0	0	-5.547
bath-34	0.426	0.326	0.263	2.722	2.712	2.727	3.246
W03G11.2	0	0	0	0.134	0.123	0.136	5.716

W03H9.1	0.09	0.098	0.079	0	0	0	-6.187
W04A4.5	0.325	0.312	0.347	0.025	0.033	0.029	-3.645
W04G3.5	0.188	0.341	0.114	0	0	0	-5.735
W06A7.4	13.733	12.443	15.7	4.661	4.456	4.052	-2.068
nmat-1	0	0	0	0.664	0.644	0.606	7.283
srxa-1	0.993	0.657	0.652	0	0	0	-7.759
srxa-3	0.187	1.127	1.087	0	0	0	-8.519
srxa-6	1.845	1.559	1.89	0.031	0.03	0.033	-5.825
oac-54	0	0	0	0.203	0.235	0.2	6.552
W07A12.8	1.618	1.667	1.681	9.218	9.318	9.769	2.421
W07E11.1	0.048	0.042	0.042	0	0	0	-6.63
W09C5.7	0.133	0.145	0.143	0	0	0	-6.968
W09D12.1	0	0	0	0.156	0.171	0.131	5.974
fbxa-93	1.332	1.452	1.247	0.333	0.321	0.316	-2.09
W09G3.6	0.022	0.045	0.053	0	0	0	-6.052
ttr-17	0	0	0	0.821	0.852	0.815	7.078
Y7A5A.3	0.233	0.257	0.209	0	0	0	-5.902
Y10G11A.1	0.221	0.221	0.127	0	0	0	-5.735
Y11D7A.3	0.222	0.322	0.22	0	0	0	-6.968
Y17D7B.5	0.678	0.633	0.609	0.035	0.034	0.037	-4.023
Y17G7B.8	0.335	0.555	0.463	0.034	0.033	0.03	-3.917
Y17G7B.10	0.046	0.043	0.033	0.222	0.123	0.169	2.187
Y17G7B.12	0.334	0.434	0.453	0.091	0.091	0.097	-2.305
Y18D10A.2	2.229	1.97	1.97	0	0	0	-8.881
Y18D10A.8	0.088	0.087	0.085	0	0	0	-5.902
Y32B12B.1	0	0	0	0.224	0.212	0.292	5.716
Y32B12C.1	3.45	3.46	3.976	0.521	0.531	0.507	-3.077
Y32F6A.5	0.485	0.453	0.281	0	0	0	-7.417
Y32F6B.1	0.431	0.345	0.117	0.012	0.014	0.012	-3.222
Y37A1A.2	0	0	0	0.399	0.31	0.32	7.283
Y37D8A.8	2.453	2.256	2.301	0.432	0.421	0.407	-2.576
hpo-21	0	0	0	0.288	0.219	0.274	5.974
Y37H9A.2	0.945	0.91	0.99	0.127	0.163	0.166	-2.669
Y38F1A.1	0.678	0.622	0.439	0	0	0	-7.177
Y38H6C.7	1.045	1.229	1.091	0	0	0	-5.902
Y38H6C.23	0	0	0	0.431	0.439	0.425	5.974
Y38H8A.1	3.133	3.443	3.104	0.034	0.031	0.035	-6.438
epg-6	0.045	0.067	0.063	0.322	0.312	0.3	2.11
lron-10	0.348	0.233	0.28	0	0	0	-6.425
swt-4	2.232	2.466	2.239	0.533	0.552	0.547	-2.142
clec-163	0.433	0.633	0.263	0	0	0	-6.722
mlt-3	0.232	0.224	0.216	0	0	0	-6.531
sre-17	0.268	0.246	0.242	0	0	0	-5.547

Y39E4B.6	0.004	0.004	0.009	0.124	0.134	0.142	3.638
faah-6	0.056	0.077	0.08	0	0	0	-5.547
Y41E3.8	7.991	7.344	7.11	1.234	1.467	1.475	-2.381
fcd-2	0.007	0.005	0.009	0.057	0.059	0.059	2.569
srt-47	0.134	0.433	0.155	0	0	0	-5.735
Y42A5A.1	0.031	0.012	0.011	0.441	0.342	0.121	3.263
lact-8	0.121	0.543	0.193	0	0	0	-7.041
Y42A5A.3	0.467	0.566	0.489	0	0	0	-6.425
Y42A5A.5	0.333	0.344	0.387	0	0	0	-5.735
Y43F8B.1	0	0	0	0.294	0.235	0.289	7.765
Y43F8B.2	0	0	0	0.219	0.289	0.272	6.702
Y43F8B.3	0.019	0.018	0.019	0.134	0.143	0.137	2.692
Y43F11A.4	0.435	0.445	0.468	0	0	0	-6.531
srxa-14	0.378	0.434	0.337	0.061	0.061	0.068	-2.374
srxa-15	0.255	0.289	0.215	0	0	0	-6.187
Y44A6C.2	1.234	1.112	1.308	0.123	0.113	0.126	-3.47
Y44A6D.3	0.046	0.036	0.039	0.245	0.256	0.269	2.569
Y44F5A.1	0.178	0.2	0.156	0	0	0	-6.187
Y45F10A.7	1.345	1.887	1.493	0.162	0.132	0.117	-3.769
Y46G5A.14	0	0	0	0.53	0.503	0.554	6.194
cpt-1	0	0	0	0.086	0.084	0.081	6.194
fbxa-128	0.672	0.983	0.518	0	0	0	-7.471
Y47D3A.29	0.099	0.122	0.075	0	0	0	-6.89
Y47D3A.31	2.443	2.233	2.281	0.324	0.356	0.363	-2.753
Y47H9C.8	0.133	0.165	0.144	0	0	0	-6.052
Y47H9C.9	0.335	0.345	0.582	0.231	0.123	0.102	-2.613
Y48A6B.9	0.556	0.243	0.26	0	0	0	-6.808
Y48A6C.2	0.267	0.268	0.294	0	0	0	-5.547
efhd-1	0	0	0	0.068	0.063	0.066	5.716
ztf-22	0.433	0.457	0.261	0.04	0.035	0.037	-2.867
Y48E1B.8	0.417	0.475	0.472	3.445	3.322	3.064	2.578
glrx-5	1.653	1.433	1.29	0.143	0.133	0.13	-3.367
Y49E10.10	0.567	0.756	0.529	0.233	0.133	0.122	-2.207
Y50E8A.8	0.344	0.256	0.225	0	0	0	-5.735
Y51A2B.4	0.133	0.167	0.125	0	0	0	-5.547
hmit-1.1	0.671	0.481	0.693	0	0	0	-8.44
hmit-1.2	0.143	0.167	0.169	0	0	0	-7.041
ttr-25	0.288	0.223	0.24	0	0	0	-5.547
Y51H4A.15	0.418	0.466	0.416	0	0	0	-6.63
Y51H4A.19	0.144	0.133	0.197	0	0	0	-5.547
Y51H4A.25	1.342	1.345	1.491	0.128	0.118	0.185	-3.116
rga-2	0.066	0.046	0.051	0	0	0	-5.735
mop-25.2	0.052	0.055	0.06	0.565	0.595	0.53	2.999

Y53F4B.9	0.023	0.034	0.016	0.057	0.077	0.08	2.187
Y53F4B.18	0.168	0.154	0.142	0	0	0	-6.311
Y53H1B.2	0.233	0.267	0.229	0	0	0	-5.902
Y54E2A.5	0.876	0.776	0.563	0	0	0	-7.241
Y54E2A.7	0.567	0.787	0.69	0.033	0.031	0.033	-4.384
ttm-5	0.088	0.098	0.078	0.511	0.523	0.532	2.622
Y54E5A.7	0.766	0.134	0.144	0	0	0	-6.808
Y54E5A.8	0.665	0.344	0.496	0.032	0.053	0.033	-3.917
Y54G9A.4	0.133	0.188	0.154	0	0	0	-5.735
Y54G9A.7	0.226	0.336	0.569	0.321	0.322	0.144	-2.07
Y54G11A.3	0.144	0.156	0.112	0	0	0	-6.052
dmsr-6	0	0	0	0.208	0.277	0.201	6.702
Y56A3A.7	0.346	0.322	0.182	0.037	0.039	0.035	-2.456
sdz-33	0.335	0.533	0.506	0.132	0.136	0.108	-2.305
Y57A10A.29	0.322	0.563	0.303	0	0	0	-6.052
Y57A10B.2	0	0	0	0.773	0.727	0.727	6.963
tag-273	0.047	0.055	0.047	0	0	0	-5.547
irld-18	0	0	0	0.315	0.368	0.323	6.194
Y57G11C.9	0.167	0.212	0.156	0	0	0	-6.808
Y57G11C.18	0.892	1.227	0.721	0	0	0	-8.978
anoh-2	0	0	0	0.064	0.067	0.069	5.974
Y57G11C.43	0	0	0	0.218	0.217	0.233	6.194
gck-3	0	0	0	0.115	0.115	0.133	6.384
gcl-1	0.405	0.487	0.507	0.017	0.018	0.016	-4.994
Y62F5A.10	0.998	1.011	1.011	0.022	0.021	0.023	-5.44
ceh-91	0.443	0.334	0.203	0	0	0	-7.241
Y66D12A.3	0.678	0.755	0.844	0.123	0.112	0.177	-2.353
ceh-92	0.457	0.348	0.208	0	0	0	-7.417
Y66D12A.7	0.312	0.356	0.368	0.034	0.033	0.035	-3.39
Y66D12A.21	0	0	0	0.324	0.358	0.369	6.194
Y67H2A.7	0.064	0.062	0.069	0	0	0	-5.735
hex-5	0.044	0.026	0.022	0.117	0.127	0.168	2.737
Y71A12B.10	0.33	0.344	0.205	0.031	0.038	0.039	-2.456
Y71A12B.12	0	0	0	0.088	0.081	0.084	5.974
Y71A12B.15	0	0	0	0.115	0.152	0.172	6.963
Y73F8A.5	0	0	0	0.059	0.059	0.057	5.974
Y73F8A.26	0.345	0.432	0.204	0	0	0	-7.111
gyg-3	0.133	0.123	0.133	1.582	1.855	1.821	3.609
Y75B8A.23	0.523	0.533	0.553	0	0	0	-5.902
zip-12	0.133	0.102	0.11	0	0	0	-5.547
Y75B8A.37	0	0	0	0.534	0.522	0.507	5.716
bath-36	0.122	0.133	0.106	0	0	0	-5.547
Y76A2B.4	0.165	0.132	0.146	0.701	0.779	0.799	2.302

Y79H2A.4	2.222	2.127	2.729	0.323	0.321	0.325	-3.162
nhr-243	0.204	0.226	0.276	0	0	0	-6.968
Y87G2A.1	0.034	0.054	0.054	0	0	0	-5.547
gpi-1	0	0	0	0.301	0.35	0.315	7.622
Y105C5A.14	0.223	0.21	0.237	0	0	0	-6.531
Y105C5A.22	0.367	0.379	0.363	0	0	0	-6.052
Y105C5A.24	0	0	0	0.101	0.15	0.158	6.384
Y105C5B.14	0	0	0	0.592	0.52	0.529	6.702
Y105E8A.8	0.014	0.013	0.012	0.674	0.388	0.396	4.708
hpo-13	0.033	0.045	0.051	0	0	0	-5.735
catp-1	0.223	0.432	0.112	0	0	0	-7.111
Y105E8A.13	0.656	0.766	0.505	0.061	0.024	0.069	-2.926
Y105E8B.7	0.354	0.346	0.348	0.062	0.07	0.07	-2.374
Y105E8B.9	0.442	0.672	0.22	0.014	0.032	0.016	-3.741
Y106G6A.1	1.119	0.99	0.933	0	0	0	-8.978
Y106G6D.8	6.566	6.266	6.066	46.133	45.913	45.132	2.779
Y106G6G.1	0.123	0.133	0.149	0	0	0	-5.902
dlc-6	0.878	0.678	0.684	0	0	0	-6.808
ska-1	0	0	0	0.232	0.332	0.194	5.716
Y106G6H.16	0.323	0.344	0.303	0	0	0	-5.735
Y111B2A.27	2.267	2.347	2.667	0.253	0.243	0.228	-3.638
Y113G7A.15	3.446	3.433	3.629	0.627	0.612	0.667	-2.551
fbxa-115	0.177	0.164	0.136	0	0	0	-5.547
clec-194	0.255	0.278	0.204	0	0	0	-6.187
Y116A8C.33	2.344	2.561	2.099	0.326	0.335	0.359	-2.659
Y116F11B.11	0	0	0	0.132	0.934	0.148	6.194
fbxa-37	0.144	0.135	0.134	0	0	0	-5.547
cls-3	0.057	0.069	0.073	0	0	0	-6.311
hhat-1	3.099	2.118	2.16	0.055	0.054	0.058	-5.289
ZC302.3	0.156	0.833	0.528	0	0	0	-7.574
gnrr-3	0.034	0.036	0.032	0.225	0.235	0.247	2.737
atfs-1	0.068	0.079	0.07	0	0	0	-5.547
ZC412.5	0.233	0.234	0.263	0	0	0	-5.547
ZC434.3	0.524	0.512	0.571	0.378	0.433	0.139	-2.122
ugt-18	0	0	0	0.165	0.105	0.117	5.974
best-22	0.122	0.122	0.102	0	0	0	-6.052
ZC518.4	4.021	5.031	4.214	0	0	0	-10.031
clec-97	0	0	0	0.39	0.398	0.309	5.974
ZK287.4	0	0	0	0.051	0.06	0.059	6.194
ztf-9	0.988	0.967	0.944	0.13	0.103	0.101	-3.3
dos-1	0.063	0.068	0.068	0.444	0.422	0.465	2.598
pyk-2	0	0	0	0.103	0.132	0.116	5.974
fic-1	0	0	0	0.134	0.193	0.116	5.974

panl-3	0.068	0.046	0.051	0	0	0	-5.547
ZK632.14	1.229	1.099	0.941	0.053	0.054	0.052	-4.169
asna-1	0.436	0.675	0.384	0	0	0	-7.302
ZK637.12	19.355	18.655	18.552	121.783	122.233	123.291	2.617
glb-1	0.17	0.18	0.104	0.711	0.767	0.711	2.613
ZK669.3	11.552	11.882	11.181	2.567	2.9	2.524	-2.26
ZK669.4	0	0	0	0.109	0.171	0.177	6.552
ZK822.5	0.211	0.21	0.281	0.033	0.036	0.034	-3.089
ZK829.3	0	0	0	0.178	0.185	0.143	5.974
ZK856.11	0.221	0.778	0.333	0	0	0	-6.808
ZK858.2	0.357	0.545	0.306	0	0	0	-6.425
clec-91	0.045	0.046	0.046	0.433	0.447	0.473	3.149
ZK858.5	0.365	0.389	0.334	0	0	0	-7.241
ZK899.7	0.344	0.333	0.266	0	0	0	-5.735
ZK930.4	0.234	0.322	0.226	0	0	0	-5.735
ZK930.7	4.229	4.119	4.881	1.234	1.455	1.245	-2.083
nep-26	0	0	0	0.186	0.163	0.169	7.375
ZK970.7	0.123	0.133	0.172	0	0	0	-5.735
ZK970.8	0	0	0	0.373	0.325	0.376	5.974
ZK1010.8	0.333	0.267	0.284	1.378	1.267	1.344	2.121
nit-1	0.123	0.122	0.13	0	0	0	-5.547
obr-3	0.024	0.025	0.022	0.223	0.222	0.21	3.097
ZK1098.1	0.143	0.156	0.125	0	0	0	-6.63
trpp-3	0.443	0.434	0.431	0	0	0	-6.531
mrps-23	4.312	2.017	2.317	0	0	0	-8.619
ZK1128.4	0.278	0.299	0.215	0	0	0	-6.187
ttll-4	0	0	0	0.197	0.168	0.131	6.384
dcaf-1	0.032	0.023	0.021	0.332	0.223	0.224	3.25
F07H5.4	0	0	0	0.498	0.484	0.422	6.384
F07H5.5	0.046	0.043	0.048	0.239	0.224	0.288	2.421
F13G11.t1	1.543	1.344	1.304	5.118	5.238	5.796	2.004
F34H10.t1	0	0	0	2.346	2.543	2.26	5.716
T10B9.t1	3.455	3.346	3.3	0	0	0	-6.425
Y43F4A.t2	2.223	2.443	2.087	0	0	0	-5.735
Y116F11A.4	0.891	0.401	0.491	0	0	0	-7.361
srz-49	0	0	0	0.146	0.165	0.198	5.974
F01D5.4	0.443	0.367	0.377	0	0	0	-6.311
F02E9.8	4.033	4.122	4.445	0.515	0.555	0.548	-3.127
F22B8.2	0	0	0	0.369	0.386	0.337	7.078
F40F9.11	0.133	0.133	0.152	0	0	0	-5.735
F56D5.4	7.76	7.87	7.025	1.554	1.344	1.384	-2.456
F58D12.2	0.145	0.124	0.162	0.033	0.036	0.038	-2.173
M142.3	0	0	0	0.167	0.173	0.182	6.194

R03D7.3	0.087	0.123	0.071	0	0	0	-7.302
VM106R.1	1.782	1.554	1.638	0.05	0.057	0.052	-5.017
Y49E10.5	0.129	0.139	0.189	1.439	1.419	1.488	2.823
Y51A2A.2	0.442	0.322	0.201	0	0	0	-5.735
Y53C10A.1	0.454	0.674	0.408	0.034	0.035	0.03	-3.741
Y53F4B.38	0.267	0.223	0.278	0	0	0	-5.735
Y60A3A.11	1.23	1.345	1.008	0.212	0.254	0.219	-2.312
Y105C5A.2	1.727	0.278	0.373	0	0	0	-8.036
Y106G6D.5	0.023	0.064	0.064	0	0	0	-5.735
ain-2	0.012	0.021	0.013	0.122	0.122	0.139	3.263
srz-85	0.034	0.032	0.038	0.258	0.288	0.263	2.569
B0212.3	0.084	0.081	0.082	0	0	0	-5.902
faah-2	0	0	0	0.193	0.132	0.101	5.974
B0222.10	0.222	0.282	0.215	0	0	0	-5.902
cpna-2	0.005	0.005	0.006	0	0	0	-5.547
natc-2	0.944	0.733	0.547	0	0	0	-7.622
egg-1	0.076	0.057	0.074	0.425	0.433	0.47	2.526
B0244.9	1.813	1.402	2.401	0	0	0	-8.205
B0261.5	0.697	0.534	0.597	0	0	0	-6.968
B0273.1	0.123	0.194	0.199	0	0	0	-5.735
pot-1	0.123	0.132	0.132	0	0	0	-5.735
B0281.6	0.122	0.166	0.182	0	0	0	-5.547
B0286.3	0	0	0	0.321	0.332	0.112	5.716
anmt-1	34.223	35.212	33.271	8.053	9.013	8.053	-2.161
B0310.2	0.045	0.033	0.061	0.223	0.212	0.29	2.11
abi-1	0.055	0.042	0.049	0.235	0.257	0.27	2.315
hpo-28	2.345	2.248	2.793	0.623	0.646	0.641	-2.234
B0336.12	0.855	0.774	0.743	0	0	0	-6.187
B0336.13	5.235	5.346	5.567	1.223	1.345	1.303	-2.207
B0348.5	0.055	0.045	0.048	0.312	0.323	0.387	2.819
pho-5	0.212	0.322	0.195	0.021	0.023	0.022	-3.13
B0361.9	0.333	0.446	0.455	0	0	0	-7.417
trpp-11	0.078	0.123	0.089	0	0	0	-6.722
srz-4	3.666	3.113	3.632	0.567	0.678	0.768	-2.352
vps-51	0	0	0	0.096	0.093	0.1	6.194
B0432.1	0	0	0	0.27	0.27	0.204	5.974
B0432.10	0.223	0.441	0.205	0	0	0	-6.722
B0511.2	0.098	0.034	0.095	0	0	0	-5.547
B0511.6	0.021	0.031	0.011	0.332	0.432	0.234	4.164
B0545.4	0.676	0.733	0.815	0.132	0.321	0.111	-2.926
B0546.4	0.256	0.222	0.261	0	0	0	-6.63
C01B7.3	0.768	0.878	0.764	0.082	0.086	0.082	-3.222
C01C10.2	0.036	0.035	0.037	0.446	0.444	0.48	3.468

C01F1.3	1.443	1.663	1.286	0.243	0.245	0.233	-2.57
C01G5.5	0.332	0.322	0.31	0	0	0	-6.722
ivd-1	4.33	4.312	4.4	0.912	0.924	0.979	-2.282
slc-17.3	0.067	0.056	0.052	0.263	0.347	0.267	2.209
acdh-6	0	0	0	0.422	0.439	0.424	7.375
C02F5.2	1.67	1.455	1.002	0.156	0.155	0.156	-2.743
C02F5.5	0	0	0	0.631	0.61	0.613	6.963
C03A7.2	1.677	1.457	1.722	0.035	0.035	0.033	-5.686
C03A7.13	0.212	0.122	0.2	0	0	0	-6.052
C03B1.7	0	0	0	0.093	0.1	0.097	6.384
C03B1.10	1.331	1.067	1.085	0	0	0	-5.902
tbc-6	0	0	0	0.348	0.381	0.305	7.078
srz-60	0.256	0.268	0.201	0	0	0	-6.187
C04E6.8	0.579	0.522	0.546	0	0	0	-6.425
C04G6.4	0	0	0	0.172	0.176	0.176	6.384
C05C8.7	0	0	0	0.109	0.101	0.103	6.194
hyls-1	0	0	0	0.28	0.299	0.268	6.552
C05E4.12	0.073	0.075	0.073	1.275	1.715	1.752	4.27
lnp-1	0	0	0	0.321	0.221	0.127	5.716
C05E11.7	0.132	0.321	0.111	0	0	0	-6.052
C06A5.2	0.553	0.655	0.672	0	0	0	-7.361
C06A6.5	0.333	0.944	0.396	0	0	0	-7.574
snpc-1.1	0.222	0.103	0.175	0	0	0	-6.531
spdl-1	0.103	0.144	0.108	0	0	0	-5.902
C06A8.8	0.016	0.015	0.013	0.521	0.271	0.215	3.789
rha-2	0	0	0	0.046	0.037	0.045	5.716
C06E7.4	0.117	0.19	0.179	0	0	0	-6.63
C06G3.3	2.223	2.112	2.087	0.378	0.336	0.334	-2.718
ufl-1	0.089	0.079	0.077	0.334	0.444	0.434	2.363
C07D8.2	0.138	0.176	0.136	0	0	0	-6.052
C07G1.6	3.567	3.457	3.727	0.633	0.667	0.603	-2.735
C07G1.7	0.457	0.568	0.686	0	0	0	-7.302
C07G3.10	0.655	0.785	0.536	0.047	0.048	0.046	-3.541
btb-15	0	0	0	0.273	0.281	0.276	5.974
C08E3.13	0.311	0.321	0.306	2.421	2.431	2.407	2.823
C08G5.1	0.054	0.033	0.044	0	0	0	-5.547
C08G9.2	0.144	0.144	0.144	0	0	0	-8.413
pkg-2	0.068	0.067	0.083	0	0	0	-6.052
gad-2	0.022	0.023	0.024	0.672	0.452	0.206	2.888
C10E2.1	0.057	0.055	0.059	0.345	0.446	0.52	2.996
C10F3.7	0.134	0.156	0.198	0	0	0	-5.547
C10G8.2	0.221	0.224	0.213	1.566	1.877	1.298	2.469
C10G8.8	0.222	0.334	0.163	0	0	0	-6.311

C12D5.4	0	0	0	0.343	0.333	0.352	5.974
sre-11	0.068	0.078	0.083	0.833	0.823	0.855	3.204
C13A2.1	0	0	0	0.152	0.121	0.165	6.552
C13A10.2	0.233	0.233	0.296	0	0	0	-5.735
C13B9.2	0.221	0.268	0.205	0	0	0	-6.63
C13F10.1	3.234	3.676	3.065	0.227	0.225	0.268	-3.614
C13F10.6	0.033	0.031	0.036	0.211	0.209	0.228	2.474
C14A11.5	0.155	0.133	0.1	0	0	0	-5.735
C14E2.3	0	0	0	0.351	0.334	0.263	5.716
C15C7.4	0	0	0	0.45	0.4	0.417	5.974
C16A11.2	0.063	0.045	0.068	0	0	0	-5.547
C16B8.2	0.167	0.187	0.165	0.042	0.034	0.042	-2.044
C16D9.4	0.417	0.759	0.438	0	0	0	-7.574
ncs-7	0	0	0	0.641	0.607	0.614	6.963
C17B7.5	0	0	0	0.077	0.078	0.074	5.974
C17C3.5	0	0	0	0.592	0.523	0.558	5.974
acdh-2	0.044	0.064	0.04	1.428	1.443	1.283	4.811
C17C3.15	0	0	0	0.353	0.333	0.384	6.194
C17F3.1	1.733	1.865	1.07	0	0	0	-7.041
C17G10.6	0	0	0	0.169	0.183	0.127	6.384
dyci-1	0.045	0.067	0.062	0	0	0	-5.735
anat-1	0.333	0.267	0.315	0.021	0.022	0.026	-3.611
C18A3.1	0.122	0.342	0.257	0.022	0.025	0.029	-3.13
osta-2	0.08	0.098	0.096	0	0	0	-5.902
tiar-1	0.154	0.142	0.146	0	0	0	-6.531
C18A3.10	1.333	1.355	1.321	0.433	0.345	0.311	-2.173
C18B2.5	0	0	0	0.174	0.141	0.127	6.552
glb-5	0.496	1.268	0.417	0	0	0	-7.759
C18E3.1	0.206	0.255	0.208	0	0	0	-6.187
C18H2.1	0	0	0	0.038	0.034	0.037	5.974
ift-74	0.199	0.178	0.108	0	0	0	-6.425
C23G10.1	0.022	0.023	0.026	0.135	0.137	0.175	2.569
C23G10.7	0	0	0	0.11	0.17	0.118	6.194
C23H3.5	0.117	0.133	0.117	0	0	0	-5.902
dop-6	0	0	0	0.147	0.172	0.175	6.838
C24A11.2	1.234	1.356	1.799	0.403	0.456	0.406	-2.247
C24H12.4	0.222	0.123	0.162	0	0	0	-6.808
C25A6.1	0.543	0.788	0.422	0	0	0	-7.041
clec-266	0.278	0.289	0.261	0	0	0	-6.311
C25E10.4	0.102	0.12	0.104	0	0	0	-5.902
C25E10.10	0	0	0	0.411	0.431	0.493	6.838
dpyd-1	0.122	0.119	0.091	0	0	0	-6.722
flp-27	2.986	2.607	2.074	0	0	0	-8.642

mdt-26	0.312	0.323	0.353	1.124	1.258	1.674	2.125
C25H3.8	0	0	0	0.023	0.023	0.026	5.974
C26B2.8	2.344	2.774	2.421	0.261	0.241	0.207	-3.645
C26B9.5	0.021	0.027	0.022	0.146	0.322	0.153	2.569
rpb-2	0.056	0.044	0.038	0	0	0	-5.735
smc-5	0	0	0	0.442	0.425	0.421	8.829
C27A12.7	0.123	0.134	0.1	0	0	0	-6.187
C27A12.9	0.322	0.345	0.305	0.035	0.036	0.034	-3.239
C27D6.1	0.901	0.911	0.926	4.56	4.87	4.023	2.002
C27D9.1	0.035	0.036	0.03	0.342	0.242	0.174	2.337
C27F2.1	0.453	0.674	0.363	0	0	0	-8.036
C29F5.3	0	0	0	0.567	0.566	0.507	7.462
mrpl-32	0.029	0.023	0.029	0.334	0.322	0.396	3.47
C30G12.2	0.133	0.443	0.12	0	0	0	-5.547
C31B8.7	0.123	0.122	0.107	0	0	0	-5.735
C32B5.13	0	0	0	0.332	0.322	0.244	5.716
C32D5.10	0	0	0	0.068	0.07	0.073	5.716
C32E12.1	2.995	2.245	2.462	0.232	0.222	0.22	-3.592
C32E12.4	0	0	0	0.05	0.045	0.042	5.974
C32F10.4	3.434	3.478	3.587	0.256	0.556	0.597	-2.695
C33C12.1	0.446	0.544	0.288	0	0	0	-5.547
srb-14	1.163	0.452	0.646	0	0	0	-7.844
C33C12.7	0	0	0	0.822	0.82	0.822	6.963
nhr-139	0.133	0.145	0.139	0	0	0	-5.735
nhr-163	0.134	0.123	0.194	0	0	0	-6.187
C34D4.1	0.123	0.111	0.136	0	0	0	-6.187
C34D10.2	0.006	0.005	0.005	0.245	0.256	0.224	5.102
sumv-1	0.023	0.043	0.029	0.224	0.233	0.268	3.053
C34E10.9	0.581	0.598	0.514	0	0	0	-6.425
C34H4.1	0.222	0.221	0.248	0	0	0	-6.722
C34H4.2	0.223	0.253	0.244	0	0	0	-6.968
C34H4.5	0.042	0.043	0.041	0.256	0.223	0.281	2.598
C35A11.4	0.177	0.167	0.112	0	0	0	-6.052
C35B1.2	0.084	0.057	0.078	0	0	0	-5.547
C35D10.5	0.344	0.432	0.151	0	0	0	-5.547
msd-4	0.303	0.441	0.306	0	0	0	-5.547
vet-2	0	0	0	0.046	0.038	0.066	5.716
C35E7.7	0	0	0	0.578	0.581	0.565	6.384
C36B7.6	0	0	0	0.163	0.13	0.124	6.384
C37A2.8	7.112	7.099	7.239	1.11	1.178	1.168	-2.744
fbxa-47	0.134	0.114	0.143	0	0	0	-5.547
C39F7.1	0.766	0.655	0.664	0.068	0.048	0.078	-3.139
C39H7.4	0.589	0.878	0.562	0.057	0.068	0.099	-2.598

C41A3.2	0.068	0.078	0.074	0	0	0	-6.052
C41D11.6	1.987	1.563	1.307	0.133	0.134	0.138	-3.346
C41G11.1	0.223	0.334	0.264	0.023	0.025	0.023	-3.541
gnrr-4	0	0	0	0.133	0.131	0.137	6.384
C42C1.3	0.228	0.274	0.276	0	0	0	-5.902
ets-5	0	0	0	0.664	0.539	0.566	7.078
C43E11.12	0.115	0.125	0.152	1.228	1.822	1.814	3.426
paqr-1	0.032	0.033	0.031	0.433	0.544	0.34	3.257
dhhc-9	0	0	0	0.184	0.135	0.187	6.194
C44B7.11	1.344	1.654	1.217	0.211	0.215	0.149	-3.14
C44B7.12	0.092	0.093	0.095	2.663	2.563	2.256	4.427
C45E1.4	0.143	0.125	0.146	0	0	0	-5.547
C45E5.1	0.344	0.443	0.314	0	0	0	-7.041
C45E5.3	0	0	0	0.342	0.322	0.153	5.716
C45G9.5	0.564	0.656	0.797	0.104	0.121	0.107	-2.984
C45G9.6	0.045	0.076	0.066	0	0	0	-5.547
C45G9.10	0.122	0.132	0.109	0	0	0	-6.808
C48B6.9	1.335	1.234	1.863	0.335	0.325	0.35	-2.516
C49A9.9	0	0	0	0.046	0.068	0.095	5.716
C49C8.2	0.233	0.256	0.293	0	0	0	-5.735
cyp-33E1	0	0	0	0.527	0.227	0.275	7.078
cyp-35A4	0	0	0	0.291	0.249	0.285	7.078
C49H3.4	0	0	0	0.342	0.322	0.222	5.716
arp-11	1.224	1.099	1.371	0.232	0.222	0.211	-2.802
C50A2.3	0	0	0	0.322	0.311	0.167	5.716
C50C3.1	0.321	0.312	0.206	0	0	0	-6.63
C50E3.6	0.835	0.98	0.802	0	0	0	-8.413
npr-35	0.544	0.453	0.38	0.062	0.064	0.065	-2.625
sucg-1	0.142	0.116	0.115	0	0	0	-5.902
C50F7.5	0.333	0.231	0.131	0	0	0	-5.547
C50F7.6	0.156	0.145	0.144	0	0	0	-6.052
C52A10.1	0	0	0	0.122	0.128	0.114	5.974
C52B9.10	2.224	2.542	2.419	0.434	0.235	0.616	-2.081
lst-5	0.467	0.447	0.471	0.082	0.082	0.089	-2.474
43894	0	0	0	0.22	0.269	0.206	5.974
C53D5.5	0.1	0.059	0.089	0	0	0	-6.187
C54A12.3	1.333	1.012	1.058	0	0	0	-5.547
C54D1.7	0	0	0	0.437	0.437	0.456	6.194
sulp-1	1.645	0.356	0.542	0	0	0	-8.071
ilys-4	2.325	2.148	4.012	0	0	0	-9.186
vps-33.2	2.112	2.433	2.054	0.216	0.226	0.257	-3.109
epg-5	0.021	0.02	0.028	0	0	0	-5.547
C56E6.4	0.091	0.091	0.097	0	0	0	-5.547

lron-15	0.511	0.465	0.498	0.019	0.012	0.017	-4.962
C56G2.3	0.312	0.301	0.298	0	0	0	-6.63
C56G2.9	1.069	1.129	1.915	0.154	0.174	0.144	-3.802
CC8.2	0	0	0	0.289	0.292	0.209	6.552
D1014.5	0.602	0.903	0.301	0	0	0	-7.884
D1022.5	0	0	0	0.234	0.244	0.222	5.716
D1044.6	0.066	0.057	0.069	0	0	0	-6.425
D2007.2	0	0	0	0.802	0.824	0.83	7.283
D2092.6	0.244	0.322	0.274	0	0	0	-6.722
aagr-1	0	0	0	0.066	0.066	0.065	5.974
lec-12	0	0	0	0.123	0.13	0.193	6.194
E01A2.5	0	0	0	0.292	0.289	0.231	5.974
cyp-43A1	0	0	0	0.16	0.104	0.101	5.974
fbxb-78	0	0	0	0.123	0.126	0.161	5.716
E04F6.9	0	0	0	0.642	0.619	0.664	6.552
pink-1	0.079	0.066	0.09	0	0	0	-6.052
EGAP2.1	8.981	8.115	8.506	2.223	2.143	2.3	-2.001
scl-15	0.633	0.445	0.525	0	0	0	-6.89
F07C3.9	0	0	0	2.408	2.478	2.411	6.963
F08F1.4	0.344	0.444	0.39	0	0	0	-6.425
gos-28	0.111	0.11	0.126	0	0	0	-5.547
F09E5.8	0.122	0.231	0.167	0	0	0	-5.547
F09E5.10	0.133	0.166	0.141	0	0	0	-6.425
F09E5.16	1.356	1.964	1.184	0	0	0	-8.236
F09E10.1	0.621	0.613	0.614	6.422	6.554	6.416	3.248
rpc-2	0.044	0.054	0.037	0	0	0	-5.547
F09F7.5	0.432	0.422	0.225	0.78	0.79	0.988	2.01
F09F7.7	0	0	0	0.497	0.41	0.401	6.963
pitr-5	0.433	0.347	0.304	0	0	0	-7.622
F10E7.9	0.046	0.056	0.079	0	0	0	-5.735
F10G7.5	0.433	0.322	0.261	0.043	0.034	0.041	-2.743
F10G7.6	0	0	0	0.269	0.287	0.237	6.702
F10G7.9	0	0	0	0.063	0.061	0.065	5.716
F12A10.7	0.412	0.434	0.416	3.155	3.375	3.476	2.927
slc-17.6	0	0	0	0.082	0.083	0.08	5.716
acp-5	16.23	14.012	15.983	3.344	3.344	3.392	-2.351
F13H6.3	0.223	0.21	0.279	0.051	0.052	0.055	-2.42
F13H6.4	0.167	0.2	0.133	0	0	0	-6.187
F13H8.8	0	0	0	0.249	0.299	0.25	6.963
F13H8.9	0.433	0.344	0.398	0.024	0.027	0.026	-3.917
F14B8.6	0	0	0	0.213	0.233	0.211	6.702
F14D12.1	0.024	0.023	0.02	0.232	0.123	0.186	3.01
F15B10.3	0.144	0.122	0.192	0	0	0	-5.547

F15E6.9	0.404	0.944	0.301	0	0	0	-7.669
pud-4	98.345	94.212	98.279	6.126	6.316	6.162	-4.109
nhr-177	0	0	0	0.168	0.176	0.167	6.194
F16B4.6	0.352	0.352	0.319	0	0	0	-5.902
srbc-41	0.149	0.139	0.192	0	0	0	-5.902
F16F9.1	0.133	0.433	0.141	0	0	0	-5.547
F16H11.1	0.156	0.178	0.124	0	0	0	-6.187
F18C5.10	0.332	0.333	0.109	0	0	0	-5.735
F18E9.4	0.137	0.137	0.171	0	0	0	-5.902
F19B10.6	0	0	0	0.597	0.504	0.571	5.974
F19C7.1	0.147	0.166	0.166	0	0	0	-5.902
F19C7.8	0.346	0.177	0.244	0	0	0	-7.111
ets-6	0.937	0.339	0.139	0	0	0	-8.327
F19F10.12	0.143	0.166	0.128	0	0	0	-6.531
cgt-2	0.032	0.033	0.033	0.322	0.312	0.336	3.179
F20D6.1	0.389	0.322	0.386	0	0	0	-6.187
F20D6.10	0.108	0.17	0.103	0	0	0	-5.902
csr-1	0.045	0.044	0.038	0	0	0	-5.547
F21A9.1	0.344	0.321	0.126	0.612	0.634	0.648	2.201
F21C10.7	0	0	0	0.036	0.037	0.033	6.384
frpr-6	0.333	0.333	0.145	0	0	0	-5.735
F21E9.2	0.203	0.211	0.258	0	0	0	-5.547
F21F3.4	0.322	0.422	0.256	0	0	0	-6.722
ceh-60	0.223	0.223	0.133	0	0	0	-5.735
F22D3.5	5.581	7.452	3.681	0	0	0	-7.574
F22D3.6	0.512	0.335	0.235	0	0	0	-7.361
F22F7.2	0.131	0.141	0.108	0.689	0.634	0.678	2.513
F23F1.2	0	0	0	0.323	0.332	0.363	6.702
rpoa-49	3.223	3.627	3.274	0.224	0.835	0.852	-2.054
F23F12.3	0	0	0	0.179	0.178	0.136	6.194
F23H11.2	0.041	0.046	0.05	0	0	0	-5.547
sucl-2	0.233	0.266	0.209	0	0	0	-6.531
dct-6	0	0	0	0.053	0.054	0.055	5.716
F25E5.9	0	0	0	1.306	1.363	1.356	7.283
slc-17.8	0.009	0.009	0.01	0.168	0.157	0.167	3.789
clec-216	0.334	0.223	0.192	0	0	0	-5.735
F26F12.3	0.041	0.042	0.047	0.674	0.566	0.374	2.86
F27B3.7	0.231	0.342	0.122	0	0	0	-7.111
F27C1.3	7.266	12.222	10.217	2.034	2.055	2.063	-2.421
F27C1.4	0.832	0.922	0.732	0	0	0	-7.111
F27C1.11	0.065	0.089	0.051	0	0	0	-6.187
F27D9.3	1.438	1.644	1.781	0.273	0.283	0.234	-3.002
F27D9.4	0.688	0.878	0.655	0	0	0	-6.968

slc-28.2	0	0	0	0.091	0.092	0.092	5.716
F28E10.1	0	0	0	0.044	0.045	0.047	5.974
F28H1.1	0.012	0.013	0.015	0.234	0.234	0.207	3.47
F29B9.7	0	0	0	0.238	0.248	0.279	5.716
mrps-21	0.499	0.456	0.458	0	0	0	-6.052
F29C4.4	2.823	2.687	2.868	12.322	11.223	13.345	2.098
F30B5.4	0	0	0	0.083	0.082	0.09	5.716
F30H5.3	0.055	0.067	0.036	0	0	0	-6.052
F31A3.5	0.066	0.078	0.059	0	0	0	-5.735
mth-2	0.563	0.764	0.56	0	0	0	-9.186
mth-1	0.543	0.233	0.284	0.046	0.044	0.041	-2.892
F31E3.2	0.088	0.07	0.076	0	0	0	-5.547
ift-81	0.433	0.533	0.233	0.031	0.032	0.032	-2.926
figl-1	0	0	0	0.129	0.122	0.128	6.384
fipp-1	0.099	0.098	0.095	0	0	0	-5.902
clec-180	0.014	0.016	0.013	0.133	0.441	0.136	3.149
F32E10.5	0.666	0.723	0.12	0	0	0	-6.187
F33G12.2	0.665	0.543	0.563	0	0	0	-7.574
F35C8.5	0.177	0.146	0.179	0	0	0	-5.902
F35C8.8	1.822	1.863	0.863	0	0	0	-7.923
F35D2.3	0.166	0.177	0.146	0	0	0	-5.902
clec-137	0.223	0.278	0.293	0	0	0	-6.311
clec-139	0.132	0.111	0.185	0	0	0	-5.735
che-10	0.055	0.057	0.031	0	0	0	-6.052
F35H10.10	0.078	0.045	0.083	0	0	0	-7.041
F36D4.1	1.128	1.234	1.832	0.413	0.422	0.43	-2.191
F36H12.14	0	0	0	0.198	0.148	0.193	5.974
F37A4.6	0.424	0.632	0.504	0	0	0	-7.669
F38A5.2	0	0	0	0.093	0.092	0.099	5.974
F38B6.2	0	0	0	0.114	0.142	0.141	5.716
F40A3.6	1.342	1.675	1.289	0.232	0.222	0.213	-2.691
F40E3.3	0.339	0.203	0.295	0	0	0	-5.735
F40H3.2	0.267	0.234	0.266	0	0	0	-6.311
F40H3.3	0.433	0.667	0.306	0	0	0	-5.547
acs-11	0.044	0.043	0.046	0.566	0.544	0.487	3.254
F41C3.7	2.235	2.446	2.521	0.635	0.614	0.608	-2.157
vps-60	12.626	13.612	11.66	63.723	65.098	62.758	2.313
agr-1	0.817	0.823	0.874	5.012	5.013	5.063	2.419
F41G4.7	0.068	0.067	0.065	0	0	0	-5.547
F41G4.8	0.062	0.063	0.067	0.434	0.423	0.455	2.598
F41H10.3	5.091	5.103	5.141	1.553	1.455	1.286	-2.114
F41H10.5	3.237	2.117	2.714	0.144	0.154	0.142	-4.342
hda-6	0	0	0	0.2	0.192	0.163	7.283

sand-1	0.323	0.364	0.262	0	0	0	-7.111
fbxa-130	0	0	0	0.116	0.126	0.16	5.716
F42A9.9	13.117	12.134	15.169	1.153	1.143	1.133	-3.855
ifo-1	0.068	0.078	0.052	0	0	0	-6.187
fbxc-20	0	0	0	0.164	0.143	0.186	6.194
F43C9.1	0	0	0	0.271	0.206	0.227	6.552
F43C11.11	0.233	0.288	0.232	0	0	0	-6.425
mtch-1	0.655	0.767	0.525	0.022	0.012	0.024	-4.424
F43E2.11	0	0	0	0.333	0.323	0.327	5.716
F44E2.4	0.004	0.004	0.005	0.078	0.076	0.077	3.789
F44E2.7	0.122	0.146	0.182	0	0	0	-6.722
F44E7.5	0.067	0.033	0.095	0	0	0	-5.735
cima-1	4.553	5.773	6.267	0.955	0.966	0.937	-2.855
acs-1	0.013	0.015	0.016	0.551	0.431	0.314	2.888
F46F11.7	0.145	0.199	0.121	0	0	0	-5.547
lact-1	0.013	0.022	0.015	0.256	0.287	0.212	3.47
gadr-3	0.056	0.051	0.057	0	0	0	-5.547
F48E3.2	1.552	1.552	1.22	0.232	0.343	0.319	-2.042
uggt-1	0.045	0.068	0.04	0	0	0	-6.052
F48E8.4	0	0	0	0.067	0.063	0.064	5.974
disl-2	0.034	0.056	0.048	0	0	0	-5.547
prp-17	0	0	0	0.083	0.083	0.082	5.716
F49D11.3	0	0	0	0.172	0.117	0.172	5.716
acl-4	0.007	0.008	0.008	0.255	0.234	0.223	4.462
F52C6.14	0.322	0.234	0.198	0.017	0.018	0.013	-3.917
denn-4	0.077	0.088	0.032	0	0	0	-5.902
F52E1.3	0.266	0.255	0.238	0	0	0	-5.902
pccb-1	8.659	7.659	8.859	0.574	0.554	0.541	-4.146
ztf-13	0	0	0	0.287	0.272	0.219	6.702
F52G3.1	0	0	0	0.116	0.106	0.115	7.184
F52H2.3	0	0	0	0.383	0.328	0.398	6.194
F52H2.7	0	0	0	0.158	0.178	0.106	6.552
polh-1	2.451	2.334	2.083	0.325	0.368	0.369	-2.606
F53A9.7	0	0	0	0.572	0.521	0.559	6.384
F53A10.2	0.077	0.079	0.062	0	0	0	-5.902
F53B3.6	0.322	0.133	0.114	0	0	0	-5.735
F53E10.1	0	0	0	0.212	0.219	0.231	6.384
F53F10.1	0.146	0.534	0.194	0	0	0	-5.735
sdz-21	0.532	0.232	0.181	0	0	0	-6.187
F54E7.9	0.356	0.344	0.366	0	0	0	-6.052
ztf-1	0.877	0.479	0.812	0.083	0.084	0.085	-3.352
F54F2.7	0.888	0.768	0.847	0.141	0.171	0.107	-3.036
F54H5.3	0.143	0.155	0.168	0	0	0	-5.735

F54H12.4	0.496	0.57	0.48	0	0	0	-7.417
nlf-1	0.015	0.015	0.012	0.776	0.786	0.61	5.412
stau-1	0	0	0	0.073	0.071	0.074	5.974
F55A4.7	0	0	0	0.229	0.239	0.291	5.716
F55A4.8	0.073	0.07	0.073	0	0	0	-5.735
F55C7.2	0.543	0.511	0.57	0	0	0	-5.735
F55F8.3	0	0	0	0.066	0.07	0.065	5.974
F55G1.9	0.213	0.431	0.109	0.565	0.457	0.65	2.436
irld-35	1.565	1.788	1.48	0.093	0.093	0.094	-4.064
sago-2	0.114	0.765	0.162	0	0	0	-7.302
eme-1	0.325	0.311	0.146	0	0	0	-6.187
F56A11.6	0.671	0.678	0.612	0.063	0.062	0.07	-3.208
F56C9.7	0.311	0.388	0.27	0	0	0	-6.63
F56D2.2	0.133	0.242	0.137	0	0	0	-6.052
F56D3.1	0	0	0	0.107	0.101	0.117	5.974
F56E10.1	0.032	0.053	0.031	0.123	0.124	0.136	2.02
ttr-55	0	0	0	0.342	0.322	0.317	5.716
F56F4.3	0	0	0	0.073	0.072	0.076	5.716
lgl-1	0	0	0	0.052	0.051	0.053	5.716
ccpp-1	0.607	0.229	0.209	0	0	0	-7.884
F57B9.8	0.888	0.678	0.952	0.043	0.043	0.046	-4.426
F58A6.1	0	0	0	0.609	0.691	0.651	7.622
F58F9.6	0.445	0.544	0.348	0	0	0	-6.722
lgc-30	0.122	0.152	0.135	0	0	0	-6.311
F59A3.7	0	0	0	0.228	0.218	0.278	5.716
F59A3.8	1.076	1.124	1.013	0	0	0	-9.411
F59A6.3	0	0	0	0.088	0.087	0.083	5.974
F59B1.2	0.134	0.212	0.179	1.546	1.346	1.579	2.996
F59C12.3	0.178	0.213	0.155	0.329	0.459	0.893	2.402
F59E12.1	0.054	0.032	0.022	0.123	0.109	0.113	2.187
F59E12.6	1.877	1.988	1.342	0.134	0.124	0.14	-3.362
cgt-3	0.097	0.097	0.092	0	0	0	-5.902
F59H6.2	0.086	0.084	0.081	0	0	0	-5.902
H05C05.1	0.137	0.111	0.127	0	0	0	-6.89
H06I04.3	0.332	0.212	0.166	0.034	0.031	0.035	-2.305
emc-1	0.068	0.079	0.081	0	0	0	-6.425
H18N23.2	0	0	0	0.125	0.145	0.123	6.384
H20E11.1	0.024	0.023	0.029	0.461	0.461	0.408	3.651
bgal-2	0.233	0.333	0.203	0	0	0	-7.111
H23N18.4	0	0	0	0.131	0.123	0.135	5.974
H24G06.1	0.045	0.034	0.021	0.111	0.113	0.139	2.567
H28G03.2	0	0	0	0.336	0.357	0.354	7.765
H31G24.1	0.779	0.934	0.873	0	0	0	-6.722

H41C03.2	0.055	0.054	0.053	0.434	0.433	0.395	2.713
mps-2	0.223	0.276	0.279	0	0	0	-6.425
mcu-1	0.668	0.336	0.582	0.112	0.124	0.105	-2.561
K02D7.1	0	0	0	0.304	0.338	0.36	7.078
K02D10.4	0.293	0.212	0.191	0	0	0	-6.63
K02E7.4	0.121	0.133	0.19	0	0	0	-5.547
K02E7.10	0.086	0.081	0.085	1.727	1.827	1.266	3.737
K02E10.1	0.168	0.155	0.115	0	0	0	-5.547
teg-4	0.038	0.037	0.038	0.245	0.224	0.271	2.68
ceeh-1	0.143	0.177	0.147	0	0	0	-6.052
nep-18	0.165	0.168	0.15	0.033	0.035	0.03	-2.374
lron-7	0.188	0.199	0.134	0	0	0	-6.531
K03H9.3	0.156	0.167	0.139	0	0	0	-6.052
K04C2.2	0.089	0.022	0.085	0	0	0	-6.531
nuo-4	0	0	0	0.106	0.111	0.12	5.974
K05F1.6	0.067	0.056	0.041	0	0	0	-5.547
aptf-1	0.122	0.133	0.156	0	0	0	-5.902
K06A1.2	0.133	0.11	0.132	0	0	0	-6.052
K06A5.2	0.437	0.356	0.369	0	0	0	-7.111
K06A5.3	1.22	1.44	1.015	0.332	0.245	0.208	-2.363
nmtn-1	6.342	6.322	6.063	1.023	1.131	1.077	-2.607
vms-1	0.543	0.544	0.205	0	0	0	-7.177
K06H7.8	0.235	0.338	0.138	0	0	0	-5.735
K07B1.7	0.121	0.134	0.178	0	0	0	-6.63
K07E1.1	0	0	0	0.265	0.262	0.24	6.194
K07H8.5	0	0	0	0.281	0.212	0.234	6.194
K07H8.10	0.043	0.023	0.021	0.134	0.124	0.109	2.187
K08D12.3	0.334	0.389	0.365	0	0	0	-6.425
K08D12.4	0.344	0.357	0.295	1.322	1.343	1.346	2.046
K09C4.5	0	0	0	0.135	0.145	0.132	6.194
K09C6.6	0.21	0.245	0.295	0	0	0	-6.052
K09E2.3	0	0	0	0.313	0.335	0.301	6.552
gpcp-1	0.055	0.056	0.052	0.539	0.439	0.394	2.778
srt-54	1.677	1.345	1.145	0.124	0.124	0.14	-3.124
K10C2.6	0.289	0.244	0.268	0	0	0	-5.547
K10C9.1	0.311	0.3	0.323	0	0	0	-6.052
K10C9.7	0.612	0.347	0.668	0.044	0.054	0.039	-4.074
K10D2.1	0.067	0.047	0.044	0	0	0	-5.547
gsto-3	0.155	0.155	0.143	0	0	0	-5.547
K11C4.2	0	0	0	0.127	0.137	0.173	5.716
K11G9.3	0.084	0.087	0.079	0	0	0	-5.547
K11G12.6	0	0	0	0.135	0.135	0.194	6.963
K12H6.8	0.544	0.653	0.529	0	0	0	-6.311

K12H6.12	0	0	0	0.143	0.127	0.144	5.974
ostd-1	0.445	0.655	0.467	0.035	0.045	0.053	-3.188
M01B12.4	0.057	0.077	0.07	0	0	0	-6.052
M01H9.5	0.373	0.363	0.332	2.334	2.544	2.373	2.706
M02B7.2	0.811	0.507	0.531	0	0	0	-7.471
M02D8.3	0	0	0	0.118	0.181	0.122	6.838
asns-2	0.057	0.045	0.086	0	0	0	-5.902
M02E1.1	0.023	0.026	0.021	0.123	0.124	0.108	2.201
nhr-203	0.021	0.02	0.024	0.135	0.124	0.167	2.569
M03E7.2	1.045	1.065	1.046	0.223	0.233	0.247	-2.173
M03F8.1	0.78	0.79	0.995	0.124	0.174	0.176	-2.595
galt-1	0	0	0	0.158	0.158	0.193	6.384
rpa-2	0	0	0	0.254	0.205	0.221	6.194
M57.1	0.078	0.088	0.065	0	0	0	-5.547
M57.2	0.232	0.19	0.171	0.014	0.022	0.016	-3.39
PDB1.1	0.035	0.036	0.032	0.225	0.325	0.246	2.737
R01B10.2	0	0	0	0.842	0.852	0.822	5.716
jamp-1	0.045	0.047	0.049	0.434	0.426	0.458	3.07
R02D3.1	0	0	0	0.067	0.066	0.062	5.974
R02D3.8	0.228	0.237	0.296	0	0	0	-6.531
R02F2.1	0.032	0.024	0.022	0.244	0.233	0.23	3.149
R02F2.8	0.137	0.889	0.187	0	0	0	-6.722
irld-13	0.057	0.079	0.034	0	0	0	-5.547
R03G5.6	0	0	0	0.308	0.308	0.33	6.702
R05C11.2	0.233	0.43	0.2	0	0	0	-6.311
R05D3.3	0.279	0.434	0.274	0	0	0	-7.241
R05D3.8	5.412	5.225	5.472	1.343	1.455	1.348	-2.135
R05D3.9	0.767	0.568	0.785	0.145	0.135	0.151	-2.484
R05D3.12	0	0	0	0.057	0.057	0.055	5.974
btbd-10	0	0	0	0.121	0.107	0.102	6.194
R05F9.11	0.067	0.081	0.09	0	0	0	-5.735
R05G6.4	0.073	0.11	0.117	0	0	0	-5.547
vdac-1	0.188	0.211	0.13	0.712	0.724	0.762	2.411
R05H11.2	0	0	0	0.13	0.15	0.196	5.716
R06A10.1	0.134	0.167	0.184	0	0	0	-5.547
R06A10.4	0.234	0.254	0.241	1.452	1.542	1.173	2.161
clec-150	0	0	0	0.134	0.124	0.143	5.716
tbc-10	0.787	0.458	0.608	0.025	0.053	0.054	-3.573
fbxc-36	0	0	0	0.125	0.115	0.151	5.716
fbxc-28	0.543	0.549	0.359	0	0	0	-7.177
chat-1	0.189	0.2	0.163	0	0	0	-6.187
ztf-16	0	0	0	0.337	0.371	0.373	7.957
R09A1.2	2.432	2.411	2.511	0.344	0.356	0.307	-3.139

R09F10.8	0.143	0.156	0.107	0	0	0	-5.547
R10A10.1	0	0	0	2.099	2.988	2.21	6.838
R10F2.4	0.545	0.367	0.767	0.103	0.122	0.109	-2.867
R10F2.5	0.689	0.777	0.689	0	0	0	-7.302
R11F4.2	0	0	0	0.248	0.298	0.295	6.963
R12A1.3	0	0	0	0.124	0.134	0.137	5.716
R12E2.11	0.036	0.036	0.036	5.007	5.801	5.48	7.045
mrps-6	0.722	0.733	0.716	3.453	3.673	3.32	2.093
R12E2.13	0.77	0.784	0.995	0.045	0.064	0.044	-4.499
kbp-1	0.423	0.432	0.46	0	0	0	-6.531
dhhc-5	0.042	0.044	0.046	0.443	0.543	0.435	3.024
cra-1	0.015	0.014	0.012	0.092	0.091	0.091	2.737
R144.10	0.432	0.544	0.418	0	0	0	-6.722
igcm-2	0.355	0.445	0.306	0.066	0.06	0.058	-2.483
aakg-4	0.067	0.078	0.093	0	0	0	-5.902
T01B6.4	2.331	2.093	2.133	0	0	0	-8.777
T01D1.4	0.256	0.273	0.273	0	0	0	-6.187
nhr-211	0.124	0.445	0.128	0	0	0	-5.735
mct-5	0.145	0.167	0.125	0	0	0	-6.425
T02H6.1	0.433	0.563	0.226	0.014	0.013	0.018	-3.678
T02H6.4	0	0	0	0.298	0.292	0.296	5.974
T03F1.11	0.164	0.166	0.164	1.226	1.346	1.59	3.097
T03G11.6	0.033	0.032	0.031	0.224	0.234	0.238	2.737
T04C9.1	0	0	0	0.054	0.074	0.079	6.384
T04D1.2	0.211	0.433	0.282	0	0	0	-6.808
T05A7.3	0	0	0	0.642	0.654	0.695	6.702
T05A12.4	0.046	0.044	0.043	0	0	0	-6.311
T05B4.4	0.231	0.241	0.206	1.813	1.823	1.889	3.061
T05B11.4	0.145	0.167	0.129	0	0	0	-6.052
T05C3.2	0.045	0.124	0.088	0	0	0	-7.361
atp-4	0	0	0	0.565	0.549	0.576	6.838
lsy-13	7.663	7.003	7.311	1.443	1.233	1.303	-2.6
brc-2	0.2	0.233	0.228	0	0	0	-6.808
T07F8.1	1.229	1.349	1.866	0.253	0.265	0.25	-3.006
npr-31	0.123	0.155	0.108	0	0	0	-5.547
T07F12.4	0.016	0.017	0.018	0.412	0.482	0.421	4.27
T08B1.4	0.145	0.156	0.139	0	0	0	-6.311
mrpl-23	0	0	0	0.402	0.421	0.452	6.384
T09A12.1	0.168	0.146	0.132	0	0	0	-5.735
T09B4.3	0	0	0	0.326	0.364	0.393	6.552
T09B4.6	0.136	0.146	0.159	1.439	1.449	1.492	3.07
tin-44	0.096	0.033	0.099	0	0	0	-5.735
cct-7	0.012	0.013	0.019	0.133	0.134	0.132	2.569

T10B11.6	0.016	0.018	0.016	0.338	0.398	0.385	4.27
T10E9.2	0.012	0.014	0.016	0.124	0.125	0.112	2.569
syx-18	2.212	2.023	2.21	11.562	12.336	10.57	2.141
T11F8.1	0	0	0	0.433	0.473	0.473	7.078
fbxa-55	0.072	0.077	0.071	0.554	0.654	0.545	2.782
T12F5.2	0.177	0.219	0.191	0	0	0	-6.187
T13C2.6	0	0	0	0.069	0.064	0.068	6.194
T13G4.5	0.235	0.342	0.248	0	0	0	-6.052
T14B4.8	0.095	0.096	0.096	1.529	1.249	1.286	3.595
T15B7.1	0.234	0.333	0.238	0	0	0	-6.89
T15B7.15	0.067	0.077	0.078	0.797	0.737	0.67	2.942
T17H7.1	0.334	0.122	0.138	0	0	0	-6.722
pamn-1	0.013	0.015	0.012	0.091	0.092	0.095	2.737
best-17	0.201	0.331	0.101	0	0	0	-5.735
T19C3.5	0	0	0	0.101	0.114	0.18	6.552
T19C3.7	0.135	0.134	0.176	0	0	0	-5.547
T19D7.7	0.122	0.133	0.102	0	0	0	-5.735
T19D12.4	0.342	0.129	0.154	0	0	0	-7.417
T19D12.5	3.335	3.885	3.516	0.744	0.723	0.792	-2.262
clec-178	11.822	13.811	10.829	0.699	0.499	0.995	-3.553
T20B12.4	0	0	0	0.164	0.16	0.168	5.974
T20B12.7	0.068	0.084	0.081	0.432	0.445	0.414	2.201
T20F7.5	0	0	0	0.091	0.095	0.094	6.702
T21F4.1	0.331	0.231	0.123	0	0	0	-5.902
T21H3.1	0	0	0	0.217	0.117	0.17	5.716
T21H3.5	0.163	0.413	0.482	0	0	0	-7.715
T22D1.3	0.332	0.144	0.113	0	0	0	-6.187
T22D1.5	0.192	0.134	0.197	0	0	0	-6.531
igdb-3	0.244	0.223	0.278	0	0	0	-7.302
T22F7.3	0.068	0.078	0.057	0	0	0	-6.052
phf-30	0.131	0.141	0.114	1.78	1.98	1.013	2.999
cnp-3	0	0	0	0.281	0.21	0.26	7.283
T23C6.4	0.023	0.023	0.03	0.234	0.222	0.253	2.888
T23F2.4	0	0	0	0.731	0.721	0.714	5.716
T23F2.5	0.844	0.988	0.846	0	0	0	-6.89
ucr-2.3	2.113	2.223	2.258	0.423	0.412	0.442	-2.463
T24C12.1	0.325	0.21	0.251	0	0	0	-6.63
eat-17	0.044	0.024	0.057	0	0	0	-5.735
T25D3.4	0	0	0	0.063	0.063	0.07	5.716
T25F10.4	0.945	0.967	0.978	0.134	0.146	0.189	-2.472
acdh-7	0.014	0.031	0.014	0.21	0.212	0.266	3.925
T26C12.1	0	0	0	0.072	0.072	0.077	5.716
T27A1.3	0	0	0	0.886	0.858	0.879	7.896

T27A3.6	0	0	0	0.219	0.294	0.227	6.384
mop-25.3	1.836	0.434	0.84	0	0	0	-8.205
bath-25	0.222	0.344	0.243	0	0	0	-6.531
fip-2	0.556	0.589	0.581	0	0	0	-6.425
T27E4.7	0.323	0.263	0.267	2.224	2.436	2.631	3.346
T28D9.3	0.544	0.342	0.331	0.055	0.053	0.051	-2.743
era-1	0.878	0.656	0.514	0.022	0.023	0.024	-4.424
kel-8	0.002	0.003	0.008	0.133	0.133	0.112	3.47
W02H5.5	0.323	0.312	0.371	0	0	0	-5.902
W03A5.1	0.046	0.011	0.052	0	0	0	-5.547
W03A5.2	0.122	0.145	0.183	0	0	0	-5.902
W03F11.4	0	0	0	0.033	0.032	0.039	5.716
W04B5.3	0.031	0.023	0.032	0.234	0.236	0.202	2.474
W04C9.5	0.532	0.255	0.237	0	0	0	-6.531
W05F2.3	0.222	0.254	0.214	0	0	0	-5.902
fbxa-203	0	0	0	0.168	0.16	0.168	5.974
W05H7.1	2.556	2.446	2.635	0.733	0.723	0.703	-2.017
W06A11.1	0	0	0	0.359	0.389	0.398	6.194
gadr-4	0.07	0.057	0.079	0	0	0	-6.052
dct-11	0.569	0.779	0.916	0.133	0.211	0.146	-2.739
W06H8.4	0	0	0	0.271	0.214	0.277	6.552
W07E6.3	0.211	0.208	0.249	0	0	0	-6.63
unc-132	0	0	0	0.082	0.082	0.082	5.716
W08A12.2	0.357	0.345	0.396	0	0	0	-5.735
W08F4.1	0.455	0.41	0.453	0	0	0	-5.547
W09C3.8	0.774	0.665	0.426	0.061	0.066	0.069	-2.676
ccb-2	0.676	0.457	0.61	0.079	0.088	0.095	-2.772
W10G11.1	0.334	0.419	0.492	0	0	0	-6.722
W10G11.3	0.277	0.289	0.271	0	0	0	-6.052
Y1B5A.1	0.747	0.737	0.767	7.125	7.115	7.153	3.088
Y4C6A.4	0.123	0.311	0.393	0	0	0	-5.547
gba-4	0	0	0	0.114	0.113	0.115	5.974
cyp-33C12	0.342	0.555	0.33	0	0	0	-7.361
srt-40	0.168	0.144	0.152	0	0	0	-5.735
arx-4	0	0	0	0.152	0.126	0.155	5.716
Y9D1A.1	0.133	0.109	0.139	0	0	0	-6.052
math-44	0.012	0.024	0.079	0	0	0	-5.547
Y18H1A.1	0.334	0.443	0.234	0	0	0	-6.89
Y18H1A.2	0	0	0	0.207	0.204	0.204	5.974
hgap-1	0.094	0.096	0.085	0	0	0	-6.968
Y22D7AL.4	0.144	0.166	0.112	0	0	0	-5.735
Y22D7AL.6	0.265	0.234	0.221	0	0	0	-5.547
Y22D7AL.9	0.129	0.139	0.095	0	0	0	-6.425

ell-1	0.053	0.054	0.052	0	0	0	-5.902
Y24D9A.5	2.663	2.366	2.265	0.412	0.442	0.422	-2.53
Y24D9A.6	2.445	2.252	2.852	0	0	0	-8.861
Y32H12A.2	0.069	0.069	0.065	0	0	0	-5.547
rpb-10	0.666	0.678	0.642	0	0	0	-6.311
arl-13	0.166	0.143	0.134	0	0	0	-5.735
Y37E11AL.1	0.155	0.155	0.102	0	0	0	-5.547
Y37E11AL.4	0.533	0.544	0.506	0	0	0	-6.722
Y37E11AL.6	0.123	0.112	0.14	0	0	0	-7.302
taf-6.2	0	0	0	0.096	0.062	0.087	6.384
nape-1	0.693	1.633	0.629	0	0	0	-7.962
Y37E11B.7	0.401	0.432	0.485	0	0	0	-6.187
smut-1	1.555	1.767	1.507	0.322	0.323	0.322	-2.335
pnc-1	0.787	0.856	0.693	0.122	0.112	0.118	-2.635
Y38C1AB.5	0.679	0.555	0.484	0	0	0	-7.041
Y39A3CL.4	1.248	1.874	0.449	0	0	0	-7.759
ttc-7	0.066	0.056	0.05	0	0	0	-5.735
mltn-6	0.066	0.078	0.054	0	0	0	-5.547
mltn-8	0.078	0.063	0.069	0	0	0	-5.735
Y39G10AR.8	0.08	0.08	0.078	0	0	0	-5.547
Y39G10AR.9	0.141	0.166	0.144	0	0	0	-6.187
epg-2	1.099	1.124	1.405	0.061	0.07	0.071	-4.394
Y39G10AR.15	0.325	0.205	0.105	0	0	0	-6.311
Y40A1A.1	0	0	0	0.202	0.223	0.257	6.384
Y40A1A.3	0.351	0.166	0.177	0.621	0.644	0.623	2.215
lbp-9	0	0	0	0.432	0.422	0.403	6.194
Y40C7B.1	0.054	0.047	0.042	0.366	0.354	0.343	2.9
Y40D12A.1	0	0	0	0.238	0.283	0.23	7.283
Y41D4A.7	0.826	0.735	0.526	0	0	0	-7.669
frpr-19	0	0	0	0.134	0.133	0.139	5.716
Y41D4B.14	1.234	1.887	1.997	0.054	0.054	0.059	-5.07
hpo-6	1.344	0.473	0.424	0	0	0	-7.759
Y41D4B.17	0.874	0.488	0.415	0	0	0	-7.523
gbb-1	0	0	0	0.129	0.189	0.123	6.963
Y42G9A.2	2.278	2.178	2.784	0.634	0.676	0.614	-2.282
Y42G9A.3	0.349	0.557	0.873	0.073	0.072	0.075	-3.621
Y44E3A.1	0.335	0.334	0.367	0.069	0.068	0.07	-2.456
Y45G12B.2	0.034	0.037	0.031	0.223	0.245	0.265	2.888
Y46B2A.2	0.132	0.145	0.144	0.024	0.025	0.024	-2.672
Y46E12BL.2	0.002	0.004	0.009	0.084	0.085	0.085	3.024
srt-42	0.014	0.016	0.016	0.224	0.254	0.419	4.369
Y46H3A.4	0	0	0	1.421	1.406	1.492	7.375
Y46H3A.5	0.012	0.021	0.014	0.156	0.156	0.196	3.47

Y47G6A.15	1.335	2.663	1.335	0	0	0	-8.799
Y47G6A.18	0.103	0.11	0.17	0	0	0	-6.187
Y47G6A.21	0.123	0.222	0.183	0	0	0	-5.735
Y48G1A.1	0.542	0.322	0.202	0.021	0.021	0.022	-3.235
Y48G1BL.5	0.123	0.332	0.163	0	0	0	-5.735
Y48G1BM.6	0.057	0.056	0.054	0	0	0	-6.187
pgs-1	0.322	0.222	0.162	0	0	0	-6.311
Y48G1C.6	2.213	2.567	2.023	0.148	0.168	0.177	-3.595
Y48G1C.9	0.489	0.567	0.418	0	0	0	-6.311
Y48G8AL.12	1.885	1.654	1.751	0	0	0	-7.302
Y48G8AL.13	1.679	1.345	1.871	0.334	0.323	0.389	-2.372
Y48G8AL.15	0.978	0.856	0.704	0	0	0	-7.041
gcn-1	0.661	0.654	0.102	0.023	0.022	0.024	-2.198
frl-1	0.122	0.111	0.099	0	0	0	-7.041
cpt-3	0	0	0	0.131	0.185	0.103	6.194
Y48G9A.12	0.722	0.762	0.622	0	0	0	-7.361
Y50D4A.1	0	0	0	0.137	0.131	0.191	7.283
Y50D4A.5	0.344	0.334	0.384	0	0	0	-5.902
Y50D4B.7	0.124	0.145	0.188	0	0	0	-6.052
taco-1	0.121	0.133	0.139	0	0	0	-5.735
Y51F10.2	0.134	0.144	0.155	0	0	0	-5.902
Y51F10.7	0.612	0.624	0.692	0.054	0.064	0.041	-4.074
Y51H7C.10	0.431	0.321	0.491	0	0	0	-7.041
nol-6	0.014	0.016	0.011	0.112	0.122	0.137	3.369
Y54E10A.11	0.056	0.067	0.042	0	0	0	-6.052
Y54E10A.12	0.444	0.286	0.284	0	0	0	-7.523
mab-31	1.239	1.129	1.851	0.196	0.194	0.198	-3.33
Y54F10AL.1	0.335	0.213	0.17	0	0	0	-6.052
fbxa-9	0	0	0	0.384	0.335	0.37	7.184
fbxa-66	0.122	0.155	0.17	0	0	0	-6.187
Y54F10BM.12	0.324	0.344	0.389	0.035	0.036	0.033	-3.541
Y54G2A.12	0	0	0	0.386	0.362	0.339	6.552
Y54G2A.15	1.44	1.564	1.044	0.234	0.231	0.206	-2.42
Y54G2A.16	0.424	0.411	0.475	0.035	0.033	0.039	-3.611
Y54G2A.17	0.088	0.079	0.07	0	0	0	-5.547
ser-6	0.344	0.443	0.216	0	0	0	-6.808
Y55B1AR.3	0.122	0.223	0.274	0.032	0.031	0.036	-2.925
Y55B1BL.1	1.171	1.567	0.712	0	0	0	-8.173
cec-8	0.088	0.086	0.085	0	0	0	-6.052
Y55F3BL.2	0.078	0.078	0.054	0	0	0	-5.547
Y55F3BR.2	0.045	0.057	0.053	0.007	0.005	0.007	-2.925
madf-1	0.612	0.607	0.663	0.322	0.311	0.162	-2.122
lipl-6	0.036	0.066	0.097	0	0	0	-5.902

Y59H11AR.3	0.456	0.501	0.525	0	0	0	-6.187
sut-2	0.033	0.055	0.057	0	0	0	-5.735
vps-20	0.442	0.167	0.175	0	0	0	-5.547
Y65B4BL.4	0.121	0.212	0.136	0	0	0	-5.735
Y69A2AR.1	0.189	0.193	0.172	0	0	0	-6.531
daao-1	0.047	0.057	0.07	0.733	0.733	0.715	3.195
epg-9	0.044	0.064	0.039	0.342	0.252	0.221	2.337
Y69A2AR.18	0.135	0.146	0.179	0	0	0	-6.187
Y69A2AR.19	1.3	1.213	1.24	0.045	0.046	0.047	-4.806
Y69A2AR.22	2.457	2.231	2.844	0.465	0.477	0.417	-2.873
cdh-12	0.023	0.054	0.017	0	0	0	-5.735
Y71F9AL.2	1.24	1.344	1.539	0	0	0	-9.354
Y71F9AL.6	1.433	1.238	1.307	0.032	0.033	0.04	-5.019
Y71F9AL.7	1.11	0.202	1.203	0	0	0	-8.594
supr-1	0.068	0.077	0.057	0	0	0	-5.547
Y71F9B.2	0.101	0.162	0.166	0	0	0	-5.902
Y71F9B.6	0.345	0.543	0.567	0.024	0.025	0.02	-4.798
Y71F9B.13	0.035	0.037	0.033	0.101	0.178	0.188	2.337
Y71G12B.25	1.877	1.346	1.737	0.221	0.241	0.207	-3.169
Y71G12B.26	0.567	0.456	0.754	0.074	0.028	0.079	-3.33
Y71H2AL.2	0.477	0.41	0.444	0	0	0	-5.902
Y71H2AM.14	0	0	0	0.179	0.159	0.176	6.194
Y71H2B.5	0.066	0.077	0.044	0	0	0	-5.547
gpa-17	0.567	0.999	0.679	0	0	0	-8.356
Y72A10A.1	0.557	0.554	0.194	0	0	0	-6.052
Y73B3A.3	0.079	0.076	0.075	0	0	0	-5.735
exos-2	0.222	0.212	0.275	0	0	0	-6.63
Y73B6BL.22	1.333	1.278	1.078	0	0	0	-6.89
Y73B6BL.27	0	0	0	0.272	0.218	0.279	5.716
Y73B6BL.28	1.543	1.444	1.343	0	0	0	-7.241
Y73C8B.1	0.155	0.133	0.138	0	0	0	-5.547
Y74C9A.1	0.433	0.445	0.312	0	0	0	-6.311
Y74C10AR.2	1.812	1.923	1.086	0	0	0	-7.622
Y75B7B.1	0	0	0	0.23	0.203	0.273	6.702
cng-2	0.114	0.178	0.134	0	0	0	-6.89
fbxa-14	0	0	0	0.284	0.208	0.271	6.963
Y82E9BR.5	0.479	0.489	0.459	0	0	0	-5.902
eak-3	0.622	0.601	0.672	0	0	0	-6.89
Y92H12BL.4	0.335	0.215	0.159	0	0	0	-6.052
Y92H12BM.1	0.133	0.322	0.194	0	0	0	-5.547
Y94H6A.5	0.023	0.021	0.022	0.373	0.363	0.325	3.707
Y97E10AL.1	0.023	0.023	0.026	0.514	0.522	0.541	4.049
abhd-12	0	0	0	0.306	0.365	0.321	6.963

srsx-7	0.066	0.065	0.069	0.312	0.31	0.394	2.337
Y102E9.3	0.487	0.455	0.409	0	0	0	-5.735
use-1	0.233	0.256	0.211	0	0	0	-6.052
ift-20	0.355	0.378	0.342	0	0	0	-5.547
bet-1	0.067	0.077	0.059	0	0	0	-5.735
fbxa-21	0.522	0.589	0.525	0	0	0	-7.111
lfi-1	0.566	0.766	0.39	0.025	0.027	0.021	-4.297
ZC21.3	0	0	0	0.122	0.132	0.118	5.716
ZC196.2	0.445	0.665	0.22	0	0	0	-6.311
ZC196.3	0.223	0.213	0.215	0	0	0	-6.63
droe-8	0.014	0.033	0.035	0.234	0.226	0.242	2.598
ZC250.2	0.443	0.655	0.32	0.025	0.026	0.023	-3.802
iglr-2	1.433	1.473	1.426	7.119	7.229	7.918	2.357
ZC328.5	0	0	0	0.393	0.327	0.328	5.974
ZC395.4	1.23	1.833	1.03	0	0	0	-7.574
ztf-8	0.021	0.022	0.024	0.123	0.112	0.137	2.337
ZC395.11	1.335	1.557	1.36	0	0	0	-6.311
sek-3	0	0	0	0.168	0.142	0.148	6.384
nekl-2	0.234	0.444	0.189	0	0	0	-6.311
ZK6.8	0.167	0.133	0.104	0	0	0	-5.902
ZK105.6	0.447	0.545	0.157	0	0	0	-6.311
srh-188	0.244	0.332	0.203	0	0	0	-6.052
ZK154.1	0.807	0.751	0.711	0	0	0	-7.471
ZK180.3	0.044	0.043	0.034	0.157	0.123	0.174	2.187
ZK180.6	0.168	0.188	0.154	0	0	0	-6.968
math-47	0.145	0.174	0.142	0	0	0	-5.547
ZK353.9	0	0	0	0.226	0.242	0.246	5.974
ZK370.4	0.451	0.432	0.132	0.046	0.034	0.03	-2.221
pdhk-2	0.032	0.024	0.024	0.223	0.327	0.269	3.263
ZK370.8	0.522	0.522	0.537	0.453	0.433	0.136	-2.083
ZK418.9	0.154	0.144	0.139	0	0	0	-6.531
toe-1	0.044	0.05	0.041	0	0	0	-6.187
ZK470.1	0.289	0.333	0.281	0	0	0	-5.735
ZK470.2	0	0	0	0.983	0.983	0.989	8.618
ZK484.6	0.333	0.212	0.281	0	0	0	-6.052
ZK484.7	0.011	0.013	0.016	1.022	1.224	1.072	5.742
cblc-1	0.086	0.084	0.085	0.413	0.424	0.465	2.302
ZK563.2	0.145	0.157	0.132	0	0	0	-6.425
acp-1	0	0	0	0.296	0.289	0.29	7.078
pmt-1	0	0	0	0.181	0.128	0.123	6.194
tag-307	0.443	0.223	0.19	0	0	0	-6.311
pcp-5	0.089	0.045	0.082	0	0	0	-5.735
ZK742.3	3.667	3.445	3.865	0.091	0.092	0.093	-5.453

ZK742.4	2.455	2.544	2.635	0.063	0.062	0.069	-5.319
fbn-1	0	0	0	0.012	0.013	0.018	5.716
upp-1	0.033	0.023	0.035	0.339	0.334	0.388	3.263
ZK973.9	0.446	0.777	0.965	0.198	0.138	0.183	-2.489
ZK1055.6	0.088	0.033	0.081	0	0	0	-5.735
ZK1127.5	0	0	0	0.293	0.231	0.239	6.552
cin-4	0.048	0.066	0.045	0	0	0	-5.547
ufbp-1	0.522	0.501	0.586	0.052	0.051	0.053	-3.527
ZK1240.8	0.267	0.238	0.238	0	0	0	-6.187
ZK1248.13	0.078	0.08	0.086	0	0	0	-6.052
ZK1248.17	0	0	0	20.012	19.441	18.081	10.689
C55F2.3	453.533	430.153	450.534	132.523	114.336	119.595	-2.028
F41B5.11	3.326	3.346	3.605	0.064	0.065	0.069	-5.686
F49E7.t2	2.559	2.869	2.869	0	0	0	-6.187
R11B5.t1	2.557	2.111	2.116	0	0	0	-5.735
T09B4.t2	0	0	0	2.244	2.442	2.639	5.974
Y73B3A.17	0	0	0	0.05	0.055	0.058	6.194
B0344.1	2.543	2.554	2.296	0.456	0.521	0.529	-2.223
C33E10.9	0.416	0.316	0.161	1.622	1.622	1.648	3.195
D1065.2	0	0	0	0.205	0.247	0.228	6.194
F23C8.10	0	0	0	0.38	0.397	0.353	6.384
F25B4.3	2.118	2.045	2.757	0.511	0.59	0.584	-2.347
F44C4.6	0.211	0.222	0.21	0	0	0	-6.187
H28G03.5	2.556	2.678	2.837	0.554	0.535	0.511	-2.582
K07E8.2	0.261	0.251	0.207	0.025	0.023	0.026	-3.036
K08F11.6	0	0	0	0.135	0.115	0.155	5.716
R13D7.5	0.233	0.254	0.233	0	0	0	-5.547
T27C4.5	0.112	0.224	0.205	0	0	0	-6.187
srz-63	0.113	0.113	0.161	0	0	0	-5.735
C11H1.9	0.221	0.143	0.111	0	0	0	-5.902
F11E6.11	0.561	0.466	0.41	0	0	0	-6.63
F19B2.10	0.113	0.167	0.123	0	0	0	-6.052
nbet-1	0.21	0.3	0.272	0	0	0	-6.187
C02D5.4	0	0	0	0.129	0.139	0.187	5.716
F32D8.11	0.123	0.154	0.155	1.256	1.553	1.284	2.9
T14E8.4	0.145	0.123	0.197	0	0	0	-6.722
C56E6.7	14.559	11.922	10.904	2.567	2.345	2.774	-2.088
ZK822.6	0.035	0.034	0.036	0.672	0.652	0.622	3.789
C55A6.12	0	0	0	0.317	0.172	0.296	5.974
tag-229	0.202	0.211	0.288	0	0	0	-6.187
D1086.10	0	0	0	0.249	0.272	0.273	5.974
F42D1.4	0	0	0	0.711	0.713	0.714	5.716
F49C5.10	0.54	0.766	0.41	0	0	0	-6.63

C06C3.10	2.237	2.237	2.684	0.346	0.568	0.62	-2.218
F55C9.12	0	0	0	0.596	0.56	0.549	6.194
C30H6.12	0.444	0.446	0.376	0	0	0	-6.311
F26D10.13	0	0	0	1.322	1.193	1.019	7.184
Y45F10D.16	0.422	0.44	0.498	0.069	0.07	0.095	-2.456
Y87G2A.20	0.303	0.322	0.353	0	0	0	-6.187
R04A9.7	0.067	0.034	0.087	0	0	0	-5.547
ugt-35	0	0	0	0.176	0.161	0.151	6.384
F21H12.7	0.45	0.423	0.499	0.322	0.433	0.122	-2.114
K08D12.7	0	0	0	0.694	0.694	0.689	6.384
M02D8.7	0.133	0.122	0.105	0	0	0	-5.547
cpsf-4	0.368	0.399	0.342	0	0	0	-6.968
Y48G1C.12	0.113	0.156	0.126	0	0	0	-5.547
C33C12.11	1.129	1.324	1.896	0.343	0.563	0.265	-2.926
K09F6.11	0.3	0.279	0.277	0	0	0	-5.547
K10G6.5	0	0	0	0.577	0.574	0.571	5.974
fbxa-219	0	0	0	0.182	0.129	0.186	5.716
R12B2.8	1.335	1.559	1.88	0.135	0.136	0.134	-3.802
C06A5.12	0	0	0	0.488	0.482	0.439	5.974
D1007.18	0.367	0.355	0.341	0	0	0	-5.547
T01D1.7	0.568	0.878	0.928	0.111	0.131	0.108	-3.175
W10C8.6	1.455	1.889	1.418	0	0	0	-7.111
Y47G6A.31	0.656	0.834	0.755	0.065	0.079	0.089	-3.139
adpr-1	0.089	0.1	0.079	0	0	0	-5.547
Y102E9.6	0	0	0	0.332	0.313	0.32	5.716
F27B3.8	0	0	0	1.647	1.667	1.446	6.384
Y48A5A.3	0.331	0.301	0.318	0	0	0	-5.547
Y54G2A.42	0.445	0.665	0.519	0.134	0.157	0.111	-2.319
nsy-7	0.345	0.567	0.678	0.035	0.036	0.033	-4.344
K07H8.11	0	0	0	0.297	0.285	0.221	6.194
K09H11.10	3.234	3.444	3.368	17.811	15.118	16.826	2.199
C13A2.12	0.205	0.223	0.279	0	0	0	-6.89
C50E3.15	0.222	0.21	0.229	0	0	0	-5.735
T23B12.11	0.333	0.39	0.278	0	0	0	-6.311
ZC13.10	0.223	0.332	0.163	0	0	0	-5.735
C36B7.8	0	0	0	0.719	0.729	0.715	5.974
C29E4.14	0.422	0.488	0.405	0	0	0	-6.187
T01D1.8	0.665	0.775	0.499	0	0	0	-6.722
F26G1.9	0.063	0.064	0.062	1.134	1.124	1.12	3.951
B0303.16	0.998	0.863	0.935	0	0	0	-8.106
flp-28	16.913	10.326	16.636	0.007	0.006	0.064	-8
F52E1.14	5.923	4.346	4.993	0.427	0.417	0.469	-3.515
nhr-286	0.045	0.046	0.047	0.79	0.68	0.971	4.175

Y53F4B.45	1.452	1.245	1.325	0.116	0.176	0.162	-3.129
crb-3	0.167	0.262	0.161	4.774	4.894	4.394	4.623
Y62E10A.20	0.222	0.203	0.222	0	0	0	-6.052
ZK1005.2	102.223	104.663	101.341	5.245	5.265	5.249	-4.36
Y39D8C.2	91.443	92.244	91.514	1.032	1.22	1.05	-6.429
Y71H2AM.25	0	0	0	0.424	0.44	0.462	7.078
ZK686.6	1.544	1.542	1.22	0.223	0.234	0.283	-2.211
T07C4.12	0.229	0.219	0.288	3.551	3.781	3.144	3.306
F40F8.12	0.758	0.728	0.779	0	0	0	-6.722
F32B5.9	9.883	5.818	5.882	0	0	0	-7.962
T27A3.9	0	0	0	7.299	7.291	7.28	7.462
F54E7.10	1.446	1.589	1.587	0	0	0	-6.187
Y105C5A.28	0.377	0.399	0.362	0	0	0	-6.63
Y105C5A.31	0.999	0.988	0.911	0	0	0	-6.187
gadr-5	0.067	0.067	0.055	0	0	0	-5.735
Y37A1A.4	0	0	0	0.075	0.078	0.075	6.384
Y41E3.21	0.323	0.294	0.372	0	0	0	-5.735
T03F7.8	4.331	4.332	4.062	0	0	0	-6.722
Y71A12B.18	0.044	0.033	0.021	0	0	0	-5.547
Y75B12B.12	1.456	1.345	1.775	0	0	0	-6.187
Y41E3.22	1.229	1.222	0.911	0	0	0	-7.041
T02E1.9	3.456	3.786	3.486	0	0	0	-6.425
R01H2.8	0.226	0.545	0.578	0	0	0	-6.311
Y64G10A.13	0.522	0.556	0.5	0	0	0	-6.722
C54D2.6	0	0	0	0.234	0.244	0.241	5.716
rpr-1	1.323	1.445	1.129	0	0	0	-6.63
C29E4.15	0.232	0.288	0.223	0	0	0	-5.735
F46C3.6	0.045	0.035	0.047	1.223	1.235	1.289	4.462
ttr-42	0.544	0.568	0.363	0	0	0	-5.902
F46G11.6	0	0	0	0.381	0.315	0.348	6.194
Y102A5C.37	0	0	0	0.627	0.647	0.672	5.716
fbxc-12	0.432	0.244	0.216	0	0	0	-6.722
T03E6.9	0.013	0.013	0.016	0.255	0.223	0.213	3.47
ZK265.11	2.556	2.666	2.255	0	0	0	-5.902
C17D12.8	3.044	3.128	3.488	0	0	0	-7.302
H04D03.5	3.863	3.994	3.836	0	0	0	-7.471
F26D11.13	0	0	0	0.219	0.288	0.24	5.974
Y75B12B.13	3.233	3.222	3.201	0.452	0.482	0.421	-3.002
D1086.19	0.665	0.423	0.43	0	0	0	-5.735
T07D4.5	0	0	0	0.39	0.334	0.393	5.974
lsy-12	0	0	0	0.048	0.043	0.05	6.384
F49D11.10	0.032	0.021	0.024	0.37	0.347	0.395	3.864
C10A4.9	0.309	0.233	0.21	0	0	0	-5.735

oxy-5	0.194	0.145	0.194	0	0	0	-5.547
C08E8.8	0	0	0	1.576	1.559	1.588	7.695
ZK1037.13	0	0	0	0.866	0.822	0.817	8.126
Y17D7C.4	0	0	0	0.326	0.356	0.349	5.974
C45G9.15	2.7	2.789	2.777	0.634	0.678	0.633	-2.244
F59B2.14	0.095	0.091	0.095	0.523	0.512	0.54	2.337
C25D7.16	5.664	5.998	5.42	0.332	0.342	0.317	-4.179
Y6G8.9	0.122	0.168	0.184	0	0	0	-5.547
M03A1.8	0.564	0.757	0.436	0	0	0	-7.622
R07B5.10	0	0	0	1.063	1.434	1.491	5.974
Y47H10A.6	0.379	0.354	0.354	0	0	0	-5.547
T04F3.5	0	0	0	0.779	0.779	0.769	7.544
C15H11.13	0	0	0	0.321	0.311	0.315	5.716
C49G7.12	0	0	0	0.325	0.348	0.382	6.838
T20F5.8	0.222	0.113	0.282	0	0	0	-5.735
C06C6.10	0.932	0.966	0.948	0	0	0	-6.187
Y51A2A.15	0.777	0.811	0.848	0	0	0	-5.902
Y51A2B.11	0.301	0.333	0.393	0	0	0	-5.902
Y26D4A.21	0.233	0.221	0.275	0	0	0	-5.547
C45B11.8	0.334	0.321	0.35	0	0	0	-5.547
R102.11	0	0	0	1.415	1.449	1.443	8.229
T04A8.18	0.443	0.331	0.111	0	0	0	-5.735
ZC116.5	0.544	0.544	0.582	0	0	0	-6.187
C54D10.14	0.456	0.789	0.57	0.024	0.026	0.03	-4.321
C36A4.12	0.123	0.111	0.163	0	0	0	-6.63
Y48A6C.6	3.01	2.34	2.978	0.331	0.341	0.308	-3.376
F56A8.9	0.246	0.322	0.212	0	0	0	-5.902
F59B2.15	0.412	0.546	0.335	2.334	2.244	2.35	2.224
urm-1	0.404	0.455	0.404	0	0	0	-5.735
W10D9.6	0.445	0.445	0.233	0	0	0	-5.735
C16A11.10	0.323	0.331	0.118	0	0	0	-5.902
W09C2.7	0	0	0	1.295	1.251	1.27	6.838
Y48G1C.13	0.634	0.333	0.669	0	0	0	-6.425
Y54G2A.57	1.452	1.782	1.216	0.092	0.093	0.099	-3.702
T10H4.16	6.572	6.762	6.72	1.235	1.226	1.276	-2.483
F31C3.12	53.118	53.335	51.831	11.228	9.458	10.849	-2.353
ZK822.8	0.779	0.878	0.933	0	0	0	-6.311
T16H12.13	1.567	1.643	1.137	0	0	0	-7.669
R13A5.15	1.225	1.432	1.052	0.133	0.123	0.126	-3.144
C29E4.17	1.35	2.991	2.092	0	0	0	-7.574
Y50D7A.13	0.468	0.5	0.454	0	0	0	-6.531
F37C12.21	0.915	0.925	0.952	5.123	5.883	5.304	2.356
C44C10.13	5.113	5.322	5.819	0.329	0.339	0.386	-3.996

K11D9.5	1155.339	1123.999	1132.872	83.441	85.331	82.108	-3.901
R107.9	0.56	0.676	0.496	0	0	0	-5.735
B0496.11	13.331	14.766	15.149	0.839	0.829	0.887	-4.196
dsb-2	0.557	0.456	0.629	0.167	0.198	0.139	-2.268
ZC513.14	0.679	0.699	0.605	0	0	0	-6.968
T14B4.10	0	0	0	1.985	1.855	1.05	5.974
B0213.22	9.834	9.009	8.885	0.446	0.556	0.62	-3.893
B0213.23	3.544	3.544	3.9	0	0	0	-6.808
T27E4.11	0	0	0	0.929	0.939	0.992	5.716
C35B1.9	0.433	0.325	0.477	2.322	2.334	2.347	2.161
ZC155.8	0	0	0	0.524	0.514	0.544	5.716
F39E9.16	1.69	1.459	1.904	12.033	11.44	13.018	2.569
K08A2.9	77.866	79.562	77.162	11.14	12.46	11.99	-2.784
Y57G11C.1135	15.541	13.456	14.086	2.334	2.368	2.381	-2.673
F20G2.9	3.553	3.343	3.256	0.346	0.336	0.363	-3.239
Y48G8AL.16	1.783	1.933	1.593	0	0	0	-7.177
T13F3.10	0.333	0.443	0.321	1.755	1.711	1.737	2.292
F54D5.17	0	0	0	0.425	0.435	0.448	5.716
W05B10.6	0.233	0.432	0.227	0	0	0	-5.547
K10G4.14	2.675	2.679	2.511	0.521	0.522	0.596	-2.168
F19G12.11	0	0	0	0.825	0.847	0.908	5.974
C07B5.8	0.066	0.067	0.06	1.226	1.336	1.756	4.549
F11C1.10	0.409	0.421	0.494	0	0	0	-5.547
T26A8.6	1.325	1.335	1.511	0	0	0	-6.052
K10D6.6	0.246	0.378	0.234	0	0	0	-7.111
H16D19.5	0.133	0.144	0.146	0	0	0	-5.547
W01A8.10	0.534	0.532	0.521	0.156	0.147	0.121	-2.18
F26A10.4	0.567	0.544	0.838	0.092	0.093	0.095	-3.13
T23F11.7	1.9	1.978	1.471	0	0	0	-6.808
K11E4.7	2.122	2.445	2.139	0	0	0	-6.052
ZK867.4	1.556	1.677	1.367	0	0	0	-6.531
F14D12.7	0	0	0	1.393	1.931	1.539	6.194
F14F3.6	0	0	0	1.409	1.741	1.747	6.384
F11C1.11	0.093	0.092	0.094	1.245	1.333	1.283	3.47
ZC190.11	2.342	2.672	2.909	0	0	0	-7.177
F59B8.5	556.556	541.644	551.649	95.345	99.125	98.504	-2.6
M110.11	2.566	2.431	2.058	0	0	0	-6.311
C18C4.11	1.443	1.453	1.142	0	0	0	-5.902
Y53H1C.4	1.445	1.665	1.451	0	0	0	-5.735
F54F12.3	1.454	1.322	1.281	0.115	0.215	0.146	-3.13
B0454.12	0.668	0.444	0.553	0	0	0	-5.547
K03H9.5	1.554	1.655	1.351	0	0	0	-6.187
F25H8.8	1.9	1.889	1.858	0	0	0	-6.311

ZK40.3	2.457	2.988	2.262	0	0	0	-7.302
ZK593.13	0.236	0.276	0.263	2.402	2.302	2.02	2.737
Y92H12A.8	4.14	4.232	4.654	0	0	0	-7.177
C50F4.17	1.545	1.434	1.19	0	0	0	-5.902
C09B8.10	9.1	9.349	9.864	2.324	2.326	2.353	-2.165
ZK867.5	1.223	1.334	1.341	0	0	0	-6.052
F02D10.10	0	0	0	1.881	1.834	1.808	6.702
ZC518.6	3.623	3.902	3.391	0	0	0	-7.999
F46H6.7	3.422	3.222	3.808	0	0	0	-6.187
B0379.8	2.567	2.555	2.72	0	0	0	-6.187
C06G1.7	1.229	1.333	1.331	0	0	0	-6.052
Y17G7A.2	4.566	4.123	4.76	0	0	0	-6.968
K03H1.16	30.258	29.008	29.829	5.423	5.412	5.424	-2.57
C25F6.9	6.555	3.154	3.554	0	0	0	-7.523
T25F10.7	1.233	1.453	1.465	0	0	0	-6.89
F55E10.8	3.332	3.455	3.08	0	0	0	-6.187
Y71F9B.17	1.45	1.34	1.096	0	0	0	-5.735
Y23H5A.9	5.555	5.767	5.546	0.925	0.911	0.948	-2.625
T19B4.9	0.237	0.264	0.238	7.333	7.223	7.323	4.631
F35G12.14	3.024	2.77	2.045	0	0	0	-6.722
C09B7.5	1.669	1.811	1.877	0	0	0	-5.547
T04C12.15	0.052	0.053	0.052	0.744	0.755	0.711	3.47
C48E7.12	2.443	2.554	2.022	0	0	0	-6.311
K04A8.11	2.089	2.434	2.958	0.479	0.448	0.474	-2.718
F28E10.7	0	0	0	2.551	2.505	2.056	5.974
ZK863.10	0.434	0.634	0.338	10.658	10.458	10.849	4.835
F53H10.4	0.423	0.423	0.426	4.234	4.144	4.372	3.179
H22K11.5	0.623	0.634	0.635	5.662	5.555	5.207	2.884
Y110A2AL.16	7.998	7.882	7.683	0	0	0	-7.241
C15H7.6	1.335	1.867	1.518	0	0	0	-6.187
C03C10.10	0	0	0	1.941	1.412	1.319	5.974
C09F9.8	8.124	3.092	5.239	0	0	0	-8.071
C09D8.3	0.885	0.668	0.75	0	0	0	-5.735
B0432.17	4.335	4.113	4.5	0	0	0	-6.425
C10C5.9	2.779	2.499	2.677	0	0	0	-6.89
K07H8.14	1.789	1.567	1.533	0	0	0	-6.311
ZC64.5	1.322	1.211	1.093	0	0	0	-6.311
T24D8.10	6.777	6.117	6.681	33.822	34.114	34.829	2.258
C34F6.13	25.667	29.134	26.683	0	0	0	-10.346
C56E6.11	1.812	1.912	1.76	0	0	0	-6.187
K08E5.8	6.557	2.337	4.133	0	0	0	-7.523
T04F8.10	2.434	2.433	2.694	0	0	0	-6.63
F56G4.8	0.654	0.765	0.536	3.347	3.224	3.667	2.569

Y106G6A.6	1.332	1.442	1.161	0.235	0.224	0.238	-2.363
C05G5.8	7.026	7.226	7.588	0.447	0.467	0.472	-4.059
AC3.14	1.431	1.455	1.406	8.331	8.551	8.082	2.392
F58F12.6	1.678	1.812	1.799	0	0	0	-6.311
F43D9.7	1.443	1.414	1.074	0	0	0	-6.187
Y69E1A.9	0	0	0	3.114	3.114	3.181	7.078
Y37E3.26	0.919	0.91	0.995	5.336	5.446	5.586	2.349
C14F11.8	0	0	0	1.182	1.2	1.302	5.974
K04G11.7	2.455	2.333	2.291	0	0	0	-6.722
W06B11.5	0.467	0.459	0.49	0	0	0	-5.735
F54C9.15	1.712	1.71	1.789	0	0	0	-6.531
F55D10.6	0	0	0	1.491	114942	1.385	5.974
C14B9.11	221.323	215.343	210.327	43.422	43.411	41.441	-2.458
Y106G6A.7	3.776	3.388	3.173	0.215	0.135	0.155	-4.344
W09H1.7	8.756	8.118	8.798	1.345	1.345	1.347	-2.803
C04C11.6	2.112	2.009	2.338	0	0	0	-6.531
F10C1.12	0	0	0	2.061	2.061	2.821	7.078
T13H2.6	1.123	1.112	1.098	0	0	0	-6.89
F31E8.9	0.333	0.345	0.31	0	0	0	-5.735
F42H10.9	2.563	2.876	2.106	0	0	0	-7.302
B0496.12	2.899	2.566	2.202	0	0	0	-6.808
C33A11.5	7.545	7.567	7.532	1.433	1.456	1.431	-2.483
F35B12.11	0	0	0	1.309	1.209	1.092	5.716
F11E6.19	0	0	0	2.929	2.293	2.893	6.963
EGAP3.1	0	0	0	3.258	3.584	3.426	6.963
H08J11.3	4.788	6.634	3.836	0	0	0	-7.471
B0496.13	0.901	0.789	0.98	0	0	0	-5.547
W06A7.6	30.223	27.445	29.074	4.238	4.128	4.838	-2.699
F48C11.6	2.832	2.921	2.821	0	0	0	-5.735
ZK897.8	2.722	2.999	2.72	0	0	0	-6.89
F11C7.8	7.456	7.568	7.984	1.244	1.123	1.312	-2.697
F59B10.7	1.331	1.544	1.128	0.122	0.112	0.121	-3.28
T21B4.18	0.722	0.742	0.718	10.119	13.229	12.895	4
C35A11.5	1.088	1.442	1.015	0	0	0	-5.735
C33G3.8	13.634	14.656	17.66	2.352	2.355	2.358	-2.995
W08F4.14	10.446	9.073	9.873	0	0	0	-7.523
F08C6.9	2.933	2.783	2.683	0	0	0	-7.111
K04H4.9	1.656	1.335	1.513	0	0	0	-6.311
C14F11.9	0.804	0.678	0.828	0	0	0	-5.547
C56E6.12	1.667	1.44	1.036	0	0	0	-5.735
F42H10.10	3.79	4.113	3.851	0	0	0	-6.89
Y50D7A.14	1.636	1.644	1.638	0	0	0	-5.735
ZK867.8	0.566	0.676	0.56	0	0	0	-5.735

B0334.16	2.445	2.553	2.308	0	0	0	-5.735
T06A4.4	2.012	2.245	2.087	0	0	0	-6.722
T03G11.15	0	0	0	1.136	1.126	1.162	5.716
T04C12.16	1.877	1.787	1.471	0	0	0	-6.808
K11C4.7	1.079	1.551	1.05	0	0	0	-5.735
C02C6.4	1.333	1.453	1.382	0	0	0	-5.902
ZK381.39	1.099	1.122	1.157	0.134	0.123	0.152	-2.982
T27E4.14	0.911	0.922	0.914	7.336	7.996	7.551	2.9
VB0365.1	0	0	0	1.973	1.73	1.95	7.078
C29E6.9	5.066	3.229	4.07	0	0	0	-7.523
Y54G9A.12	0	0	0	0.836	0.836	0.856	5.716
T09B4.14	0	0	0	1.239	1.229	1.291	5.716
K10C3.8	0	0	0	1.381	1.391	1.385	6.384
F57B10.16	7.446	7.777	7.729	1.998	1.6	1.772	-2.23
C53B4.12	1.404	1.444	1.499	0	0	0	-6.052
T18H9.8	1.298	1.34	1.98	0	0	0	-6.425
R10E12.4	0.544	0.234	0.68	0	0	0	-5.902
W02A2.10	0	0	0	3.028	3.028	3.4	7.184
B0457.8	1.662	1.543	1.393	0	0	0	-5.902
C05E11.11	0	0	0	2.611	2.861	2.486	6.838
T28B11.7	0	0	0	0.555	0.545	0.546	5.716
F15C11.4	4.211	4.443	4.023	0	0	0	-6.63
B0379.9	2.224	1.49	1.498	0	0	0	-7.111
F28D1.19	1.221	1.225	1.454	0	0	0	-6.187
C05A9.3	0.768	0.778	0.781	8.118	8.767	8.846	3.362
M70.7	1.645	1.645	1.638	0	0	0	-5.735
C10H11.12	0.668	0.333	0.519	0	0	0	-6.311
H19M22.6	1.673	1.022	1.652	0	0	0	-6.968
ZK470.8	0.901	0.911	0.995	0	0	0	-5.547
B0524.8	6.545	5.224	6.577	0	0	0	-6.968
R07D5.4	1.338	1.458	1.833	0	0	0	-6.425
F28E10.9	1.877	1.434	1.142	0	0	0	-5.902
F09A5.10	3.779	3.967	3.669	0.822	0.823	0.848	-2.207
C09D8.4	1.443	1.554	1.022	0	0	0	-5.735
F46G11.10	1.297	1.923	0.967	0	0	0	-6.722
T14E8.5	1.334	1.544	1.807	0	0	0	-6.425
C52A11.5	3.306	3.188	2.316	0	0	0	-6.89
F56E3.8	0.777	0.565	0.72	0	0	0	-5.547
F49E10.12	0.218	0.282	0.287	1.61	1.613	1.635	2.337
Y24D9A.11	8433.423	8432.445	8448.499	1856.332	1856.452	1882.215	-2.281
R03A10.8	0	0	0	0.843	0.826	0.826	5.716
F13B12.13	2.344	2.765	2.116	0.333	0.322	0.301	-2.867
C54D2.9	0.128	5	0.128	2.718	2.752	2.184	3.789

C11G10.4	1.333	1.446	1.864	0	0	0	-6.531
C32C4.10	0	0	0	0.623	0.623	0.986	5.974
C30C11.6	1.543	1.432	1.12	0	0	0	-5.547
T23G5.10	4.851	4.926	4.513	0	0	0	-7.715
Y65B4BL.9	4.433	4.764	4.428	0.713	0.713	0.757	-2.625
Y111B2A.33	2.888	2.998	2.756	0	0	0	-6.187
F31C3.14	0.47	0.498	0.498	14.443	15.553	13.293	4.611
K08E5.9	1.554	1.655	1.269	0	0	0	-6.052
C16C2.6	0.801	0.811	0.898	0	0	0	-6.052
T10H10.4	0	0	0	1.39	1.898	1.639	6.194
T21B10.9	4.334	4.033	4.071	0.634	0.674	0.673	-2.672
C34F6.15	3.447	3.455	3.733	0	0	0	-6.052
R05D7.8	0.128	0.138	0.184	2.588	2.544	2.516	3.561
F42H10.11	1.334	1.211	1.246	0	0	0	-6.531
T06G6.14	0.249	0.229	0.288	2.222	2.122	2.219	2.737
F25B4.11	1.231	1.411	1.447	0.113	0.123	0.13	-3.468
B0457.9	1.244	1.211	1.269	0	0	0	-6.052
Y71F9B.18	0.891	0.999	0.803	0	0	0	-5.547
Y32F6A.7	1.403	1.412	1.465	0	0	0	-6.052
C04H4.4	1.345	1.346	1.851	0	0	0	-6.531
C09F12.6	222.043	224.542	218.068	17.713	18.773	18.731	-3.655
R04F11.10	0.912	0.909	0.992	0	0	0	-6.052
F31B12.5	1.235	1.446	1.918	0	0	0	-6.531
C33G3.9	0.112	0.123	0.134	2.259	2.529	2.292	3.789
C56E6.14	0	0	0	3.084	3.041	3.008	6.838
F47G6.6	1.365	1.342	1.333	0	0	0	-5.547
H22K11.7	1.223	1.445	1.105	0	0	0	-6.425
M79.9	1.655	1.678	1.445	0	0	0	-6.808
T06E4.17	0.666	0.617	0.564	3.133	3.155	3.134	2.315
F55C12.10	1.079	1.669	1.932	0.333	0.345	0.314	-2.676
R13A1.14	5.055	4.057	3.088	0	0	0	-7.302
B0035.19	0.902	0.781	0.926	0	0	0	-5.547
R03A10.9	0.216	0.226	0.257	2.446	2.126	2.639	3.149
F40F9.14	0.143	0.213	0.128	3.506	3.981	3.058	4.27
F01E11.8	1.444	1.656	1.19	0	0	0	-5.902
F09D5.1	0.954	0.555	0.952	0	0	0	-5.547
F22F4.8	1.212	1.656	1.44	0	0	0	-5.902
C34D10.3	0	0	0	3.584	3.543	3.518	7.375
C48B6.12	16.783	10.282	12.284	0	0	0	-8.036
F10G8.11	1.211	1.092	1.217	0	0	0	-6.531
F59D8.3	133.332	145.675	180.338	15.872	15.972	15.718	-3.634
T14F9.11	0.335	0.566	0.851	0	0	0	-6.425
Y73B3A.24	3.223	3.256	3.287	15.567	16.527	14.517	2.021

F42D1.8	0.124	0.134	0.138	5.156	5.652	5.188	4.919
T21C9.14	2.522	2.733	2.363	0	0	0	-6.808
H12I19.116	3.556	3.611	3.324	0	0	0	-6.187
F13H8.13	1.335	1.446	1.949	0	0	0	-6.425
F54D12.14	0	0	0	3.013	3.401	3.013	5.716
Y69E1A.10	0.822	0.812	0.85	0	0	0	-6.052
F58H1.12	1.422	1.409	1.465	0	0	0	-6.052
F55G11.13	2.676	2.445	2.031	0	0	0	-7.302
F26D12.99	8.567	8.989	8.685	0.587	0.597	0.571	-3.917
C17E4.15	1.878	1.984	1.516	0.116	0.176	0.162	-3.222
F38B7.13	1.343	1.654	1.388	0	0	0	-5.547
W10C8.9	1.733	1.633	1.415	0	0	0	-6.187
T01B6.7	0.258	0.288	0.278	1.349	1.569	1.9	2.569
F14F3.8	0	0	0	0.286	0.323	0.532	6.194
Y54G9A.13	5.124	5.167	5.812	1.324	1.376	1.37	-2.186
C17C3.22	0.146	0.136	0.16	68.497	60.187	66.185	8.372
C40C9.11	1.282	1.722	1.215	8.235	8.346	8.54	2.677
F40H3.8	0.667	0.612	0.611	0	0	0	-5.547
K02A4.3	0	0	0	2.12	2.197	2.301	7.184
ZC477.16	0.454	0.654	0.42	0	0	0	-5.735
W01A11.10	0.779	0.989	0.79	0	0	0	-5.902
F09E5.19	1.222	1.445	1.269	0	0	0	-6.052
F56D1.8	1.322	1.443	1.048	0	0	0	-6.311
R07E4.9	2.226	2.336	2.626	0	0	0	-6.722
B0379.10	1.733	1.756	1.719	0	0	0	-6.425
EGAP7.2	1.506	1.661	1.101	0	0	0	-5.547
R106.4	0	0	0	2.349	2.539	2.393	5.716
F58A4.18	0	0	0	1.644	1.654	1.644	5.716
F20D1.13	0	0	0	2.698	2.657	2.646	6.702
F55F3.5	1.54	1.443	1.022	0	0	0	-5.735
R03E9.6	0.644	0.677	0.643	3.123	3.223	3.299	2.209
B0304.10	9.112	9.998	9.091	0.359	0.529	0.586	-4.005
F22B3.13	1.877	1.557	1.606	0	0	0	-5.547
F26A10.10	1.445	1.676	1.304	0	0	0	-6.052
F46E10.14	1.034	1.032	1.022	0	0	0	-5.735
F33D4.12	52.122	52.123	52.405	7.546	7.346	7.594	-2.899
F18E2.10	0.334	0.331	0.383	1.999	1.933	1.966	2.187
F08G5.12	1.555	1.887	1.947	0	0	0	-5.902
M01G5.8	3.557	3.335	3.886	0	0	0	-6.052
C14F5.7	1.459	1.656	1.904	0.233	0.253	0.232	-3.031
C01F4.5	2.155	2.667	2.116	0	0	0	-5.547
ZC477.17	6.143	6.109	3.195	0	0	0	-7.361
B0334.17	1.453	1.321	1.073	0	0	0	-5.735

C04D8.2	1.923	3.944	3.944	0	0	0	-7.574
T14G11.6	198.345	189.008	188.524	35.458	33.276	34.765	-2.553
F13H6.9	0.436	0.346	0.355	1.679	1.346	1.579	2.02
C01F6.12	1.477	1.745	1.771	0	0	0	-5.735
F44E7.13	4.022	4.671	4.145	0.624	0.678	0.664	-2.718
C09D8.6	4.335	4.844	4.847	0	0	0	-6.531
K04D7.11	1.143	1.144	1.104	0	0	0	-5.735
C56E6.15	0.633	0.555	0.696	0	0	0	-5.735
K11D5.1	7.079	7.79	7.48	0	0	0	-7.177
T01G1.5	1.645	1.89	1.485	0	0	0	-6.187
ZK265.13	1.555	1.666	1.55	0	0	0	-5.547
F47B8.16	0.235	0.245	0.254	2.234	2.346	2.604	3.149
C36F7.12	1.566	1.995	1.454	0	0	0	-6.187
E01H11.4	1.889	1.678	1.889	0	0	0	-6.425
C31H1.96	1.555	1.345	1.598	0	0	0	-6.311
W10C8.10	1.335	1.545	1.544	0	0	0	-6.311
F55C10.9	0.679	0.989	0.609	0	0	0	-5.547
F18A11.8	1.843	1.335	1.847	0	0	0	-6.425
C45E1.6	4.113	4.679	4.695	0.435	0.245	0.446	-3.39
D1044.11	1.045	1.055	1.065	0	0	0	-5.735
K10D6.11	1.556	1.467	1.579	0.116	0.126	0.159	-3.309
C54D1.10	0	0	0	1.016	1.028	1.022	6.194
T06E8.3	3.244	4.639	4.265	0	0	0	-7.523
F55A12.13	0.789	0.878	0.933	0	0	0	-6.311
K03H9.8	1.679	1.787	1.858	0	0	0	-6.311
T19E7.24	2.761	2.799	2.135	0.212	0.323	0.292	-2.926
H21P03.6	1.334	1.166	1.134	0.085	0.086	0.081	-3.802
C54G4.11	0.789	0.545	0.735	0	0	0	-6.187
M195.6	0.79	0.756	0.769	0	0	0	-5.735
C18F3.7	1.557	1.155	1.139	0	0	0	-5.547
K03H4.3	0.557	0.777	0.683	0	0	0	-5.735
Y104H12D.7	2.557	2.888	2.285	0	0	0	-5.902
F26A10.11	0.674	0.698	0.638	0	0	0	-5.547
F25B4.12	0.474	0.494	0.44	3.01	3.23	3.01	2.613
F53B1.10	1.877	1.654	1.606	0	0	0	-5.547
F16A11.6	4.334	4.124	4.443	1.345	1.256	1.085	-2.128
C05D2.14	4.237	4.117	4.73	0.631	0.651	0.606	-3.038
F11E6.20	1.444	1.766	1.544	0	0	0	-6.63
K12C11.8	1.145	1.145	1.182	0	0	0	-5.902
C23H4.11	1.441	1.653	1.084	0	0	0	-5.547
K08A8.11	1.665	1.043	1.049	0	0	0	-5.547
T22A3.11	18.423	13.433	18.426	4.812	4.824	4.899	-2.019
B0336.14	0.457	0.537	0.98	0	0	0	-5.547

K08E3.13	84.335	82.335	87.781	9.029	9.309	9.087	-3.384
T14F9.12	4.563	4.928	4.214	0	0	0	-7.471
K03H1.18	0.318	0.313	0.381	2.566	2.336	2.604	2.598
F10C1.15	2.331	2.556	2.15	0	0	0	-6.531
T24F1.9	2.205	2.305	2.055	21.011	24.033	22.009	3.293
Y42H9B.5	3.988	3.954	3.476	0.71	0.727	0.775	-2.244
T21G5.7	5.442	5.552	5.232	0.909	0.949	0.994	-2.483
F26H11.9	0	0	0	3.566	3.966	3.56	6.194
C11G6.5	0.123	0.133	0.128	1.567	1.687	1.747	3.47
T02E9.16	4.011	3.503	3.001	0	0	0	-7.715
F29B9.14	0	0	0	1.501	1.589	1.519	6.194
C41A3.7	1.89	1.997	1.777	0	0	0	-7.111
H05O09.3	0.645	0.609	0.7	0	0	0	-6.052
T13B5.10	0.812	0.779	0.862	4.224	4.433	4.418	2.226
R151.15	0.899	0.879	0.929	0	0	0	-5.735
F58H1.15	1.667	1.788	1.215	0	0	0	-5.902
C29E6.12	0.91	0.912	0.966	5.127	5.097	5.66	2.411
W01A11.11	0.458	0.988	0.842	0	0	0	-5.735
K08A2.12	2.548	2.656	2.793	0	0	0	-6.187
B0403.9	5.448	9.421	5.401	0	0	0	-8.036
F14B8.11	8.098	8.918	8.796	45.234	47.123	46.165	2.275
W08F4.16	0.448	0.833	0.802	0	0	0	-6.722
F52D10.9	0.674	0.664	0.639	4.664	4.784	4.369	2.622
R12E2.18	0	0	0	3.717	3.771	3.792	6.963
M163.17	0	0	0	3.429	3.487	3.462	7.695
F52B10.14	0.677	0.867	0.702	0	0	0	-5.735
K01G5.12	0.335	0.312	0.323	1.856	1.878	1.837	2.219
K08E5.11	3.227	2.989	2.657	0	0	0	-6.89
AH10.8	3.145	3.764	3.557	0	0	0	-6.808
C56G2.18	11.122	9.332	9.139	0	0	0	-8.881
Y67D8C.16	1.112	1.201	1.269	0	0	0	-6.052
T03F7.12	2.234	2.444	2.584	0.468	0.433	0.465	-2.533
ZK20.8	0	0	0	3.121	3.21	3.512	7.283
K03C7.8	2.667	2.655	2.666	0	0	0	-5.547
C27A2.11	2.13	2.153	2.101	24.33	26.233	25.035	3.453
F13H6.13	0	0	0	0.469	0.466	0.456	5.716
Y51A2D.29	1.481	1.451	1.104	0	0	0	-5.735
C08G9.5	4.678	5.133	4.139	0	0	0	-7.041
D2089.6	1.222	1.211	1.269	0	0	0	-6.052
F53B2.14	0.945	0.57	0.966	0	0	0	-5.547
F20D1.15	0	0	0	3.44	3.396	3.844	7.283
F48A9.4	12.224	10.114	10.42	0.666	0.654	0.66	-4.053
C45G7.8	3.678	3.345	3.611	0	0	0	-6.187

F43G9.15	9.223	9.56	9.012	1.513	1.512	1.519	-2.667
F41D9.11	1.126	1.779	1.599	0	0	0	-7.177
C47E12.16	1.441	1.541	1.058	0	0	0	-5.735
Y39H10A.8	1.178	1.451	1.139	0	0	0	-5.547
ZK377.5	2.787	2.999	2.757	0	0	0	-7.111
F46F3.9	0.344	0.366	0.372	1.369	1.569	1.905	2.201
T27A10.8	234.239	235.034	237.087	21.534	23.564	23.544	-3.446
F48B9.10	0	0	0	4.292	4.215	4.231	8.072
K02G10.10	3.134	3.872	3.173	0	0	0	-6.722
B0001.11	1.564	1.554	1.406	0	0	0	-6.187
K08H10.16	12.207	11.023	12.069	3.552	3.345	3.201	-2.024
K12G11.9	3.777	3.868	3.681	0.724	0.834	0.868	-2.173
C06E8.7	8.562	6.167	8.173	0.727	0.717	0.768	-3.502
F56A11.10	0.126	0.166	0.156	2.677	2.676	2.668	3.789
C15A7.6	0	0	0	0.808	0.881	0.838	5.974
M01G5.10	5.445	5.776	5.273	0	0	0	-6.89
C38C5.2	1.433	1.453	1.818	0	0	0	-6.968
F14D12.8	1.346	1.674	1.396	0	0	0	-6.187
ZK1010.11	6.834	6.812	6.84	34.776	39.446	37.601	2.339
W01H2.6	4.339	3.455	3.947	0	0	0	-6.808
C53D6.19	0	0	0	2.485	2.685	2.855	5.716
D1054.20	3.242	2.134	3.124	0	0	0	-7.715
F55A4.12	0	0	0	1.516	1.516	1.571	6.194
C13B4.5	1.454	1.674	1.388	0	0	0	-5.547
C30F2.6	0	0	0	1.326	1.323	1.261	5.716
C29F3.9	33.121	30.123	33.256	0.817	0.827	0.868	-5.341
R53.10	2.344	2.544	2.335	0	0	0	-6.425
T04C9.7	0.777	1.223	0.66	0	0	0	-7.041
F18A11.9	2.89	3.119	2.856	0	0	0	-6.89
C25F6.16	0.565	0.455	0.458	2.249	2.342	2.191	2.11
T04C10.9	0.612	0.613	0.685	3.345	3.457	3.512	2.209
T25D3.6	4.888	4.989	4.76	0.331	0.321	0.313	-3.917
C06G1.12	2.756	2.554	2.556	0	0	0	-7.041
ZC64.13	1.231	1.441	1.088	0	0	0	-5.735
T07D1.10	2.332	2.155	2.192	0	0	0	-6.722
Y54E10A.24	0.591	0.561	0.508	3.227	3.347	3.688	2.7
Y17G7A.3	3.448	3.256	3.808	0.813	0.335	0.868	-2.221
H21P03.7	0	0	0	0.621	0.614	0.612	5.974
Y71F9B.19	0.555	0.775	0.52	0	0	0	-5.735
K03H4.4	1.331	1.209	1.088	0	0	0	-5.735
Y105E8A.42	0.69	0.694	0.691	0	0	0	-5.547
Y75B8A.50	0.679	0.944	0.932	0	0	0	-5.547
Y51H7C.23	4.222	4.555	4.847	0	0	0	-6.531

C09D8.8	1.023	1.11	1.096	0	0	0	-5.735
F47G3.8	3.447	3.323	3.651	0.267	0.637	0.669	-2.526
C30A5.13	1.382	1.3	1.322	0	0	0	-6.052
K03E5.6	1.546	1.589	1.575	0	0	0	-6.187
R04F11.16	1.542	1.2	1.174	0	0	0	-5.902
D2024.14	1.412	1.489	1.421	0	0	0	-6.63
F07C3.14	0.976	0.68	0.974	0	0	0	-6.187
K11H3.10	1.934	1.811	1.82	0	0	0	-6.425
W04D2.9	6.432	6.432	6.347	1.123	1.124	1.107	-2.611
F12F3.6	0.656	0.645	0.65	0	0	0	-5.547
C12D12.9	0	0	0	1.843	1.835	1.874	6.838
Y18D10A.28	1.444	1.325	1.451	0	0	0	-5.735
K08F8.10	4.557	4.761	4.709	1.142	1.223	1.225	-2.043
K03H4.5	0.644	0.623	0.629	0	0	0	-5.547
K02A4.7	0.955	0.912	0.974	0	0	0	-6.187
C46H11.12	0.668	0.346	0.793	4.127	4.997	4.746	2.436
T13B5.11	0	0	0	2.804	2.836	2.83	5.974
Y39H10A.9	2.459	2.769	2.929	0	0	0	-5.735
F25D7.6	1.702	1.689	1.785	0	0	0	-6.311
C12D8.23	0.237	0.247	0.272	6.79	6.24	6.974	4.369
F47B10.10	1.313	1.334	1.043	0	0	0	-5.735
F44E8.44	4.334	4.973	4.165	0	0	0	-6.531
W06B3.5	2.435	2.457	2.468	0	0	0	-5.547
F12F6.15	1.231	1.211	1.127	0.135	0.157	0.193	-2.606
F26A10.14	1.655	1.889	1.328	0	0	0	-5.902
F11A6.9	0.25	0.246	0.297	10.447	10.667	10.679	4.852
ZK617.14	0.568	0.522	0.592	0	0	0	-5.547
C16D9.14	0	0	0	2.968	2.868	2.959	6.384
M04C9.9	88.456	83.445	87.859	22.728	21.723	20.782	-2.193
F42C5.13	1.267	1.233	1.278	0	0	0	-6.052
T06E8.4	1.91	1.12	1.98	0	0	0	-6.425
F28A12.5	0	0	0	1.22	1.798	1.722	6.702
F02E8.8	0	0	0	2.631	2.614	2.613	6.838
H40L08.4	0.256	0.246	0.263	2.567	2.547	2.693	3.149
F11D5.18	0	0	0	2.166	2.662	2.872	6.963
K08A8.15	2.567	2.666	2.556	0.613	0.633	0.655	-2.044
C15H7.8	1.343	1.368	1.313	0	0	0	-6.052
D2030.14	1.021	1.222	1.015	0	0	0	-5.735
F01G12.12	0.756	0.734	0.699	3.346	3.546	3.583	2.201
T05C12.14	0.334	0.312	0.377	1.91	1.943	1.933	2.201
Y50E8A.21	0	0	0	3.777	3.702	3.708	6.552
Y48A6C.7	2.522	2.533	2.515	0	0	0	-5.547
C49A1.14	0	0	0	2.243	2.143	2.325	5.716

K11E4.9	3.766	3.877	3.173	0	0	0	-6.63
F17E5.4	1.334	1.322	1.005	0.141	0.121	0.107	-3.222
F08G5.14	0	0	0	3.418	3.48	3.452	7.765
T25D10.6	5.602	3.335	3.057	0	0	0	-7.844
C14A11.11	31.224	27.224	30.386	7.478	7.867	7.802	-2.074
F28C1.13	2.349	2.113	2.877	0	0	0	-7.111
F58A6.12	0	0	0	1.321	1.311	1.107	5.716
F58A4.20	2.457	2.347	2.683	0.41	0.433	0.437	-2.676
Y58A7A.11	3.445	3.776	3.022	0	0	0	-6.052
ZC250.9	0	0	0	1.841	1.941	1.998	7.184
F55B12.14	0	0	0	3.963	3.928	3.939	7.622
H22K11.9	1.934	1.234	1.946	0	0	0	-6.531
F30H5.7	2.335	2.024	2.784	0.659	0.613	0.666	-2.163
C44E12.4	0.933	0.901	0.929	0	0	0	-6.052
T26A5.10	2.897	1.567	1.897	0	0	0	-7.523
F36G9.18	0	0	0	40.193	34.456	39.862	10.671
T25D3.7	0.513	0.555	0.595	0	0	0	-5.547
T27E4.18	0	0	0	4.636	4.936	4.649	6.384
C44H4.10	195.914	193.339	198.937	8.29	8.19	8.903	-4.595
T27B1.4	1.433	1.489	1.41	0	0	0	-6.052
F53B1.12	4.215	2.533	2.499	0	0	0	-7.802
ZK180.11	0	0	0	1.232	1.212	1.242	5.716
F26F4.14	0	0	0	2.143	2.126	2.184	6.702
F52B10.16	0	0	0	1.349	1.386	1.337	5.974
F49E10.15	0.756	0.878	0.765	0	0	0	-5.735
W01A8.11	5.18	3.797	3.808	0	0	0	-7.622
K08A8.16	0.61	0.632	0.66	0	0	0	-5.547
T24B8.14	11.257	12.777	12.566	1.233	1.633	1.302	-3.324
Y73B6BL.276	1.55	1.23	1.022	0	0	0	-5.735
F12F6.16	0.556	0.992	0.762	0	0	0	-5.902
T08G2.6	1.535	1.568	1.55	0	0	0	-5.547
F22D3.8	1.335	1.882	1.215	0	0	0	-5.902
F54D12.16	2.453	2.788	2.38	0	0	0	-5.547
M01G4.6	0	0	0	6.48	6.319	6.315	7.078
Y39A1A.28	4.344	4.223	4.008	0.611	0.645	0.623	-2.772
Y105E8A.43	1.333	1.433	1.403	0	0	0	-5.547
F25H8.12	7.451	4.522	6.551	0	0	0	-7.715
ZC455.16	1.445	1.655	1.35	0	0	0	-6.052
F14F3.11	2.099	2.213	2.596	0.22	0.23	0.296	-3.13
W06A7.8	5.446	5.118	5.788	0.426	0.256	0.26	-4.462
B0563.14	0	0	0	3.026	3.059	3.088	7.078
F28E10.12	0.987	0.945	0.907	0	0	0	-5.547
F42H10.13	3.446	3.339	3.9	0.728	0.724	0.784	-2.374

W01B6.15	0.235	0.223	0.254	5.222	5.142	5.207	4.143
C13G3.8	2.877	2.456	2.026	0	0	0	-6.63
T04C9.8	0	0	0	1.312	1.812	1.873	6.384
F56D1.11	1.422	1.774	1.451	0	0	0	-6.722
F11E6.21	2.545	2.878	2.07	0	0	0	-6.63
K04G11.8	1.019	1.209	1.094	8.86	8.96	8.604	2.823
T16G1.16	4.667	4.877	4.67	1.226	1.283	1.228	-2.016
T28B11.13	0	0	0	3.016	3.902	3.39	7.283
B0285.16	0.759	0.755	0.702	0	0	0	-5.735
Y76F7A.4	4.667	5	4.57	0	0	0	-7.302
T16G1.17	0.433	0.478	0.44	0	0	0	-5.547
T13H10.11	1.322	1.043	1.002	0	0	0	-6.531
T03G11.20	0.889	0.9	0.842	0	0	0	-6.052
T25F10.11	0.788	0.744	0.74	0	0	0	-5.547
F25B5.11	2.023	2.456	2.158	0	0	0	-6.808
Y62F5A.13	3.133	4.771	4.111	0.237	0.337	0.37	-3.468
W03G11.16	0.77	0.917	0.979	0	0	0	-6.187
F59G1.12	4.118	2.446	2.172	0	0	0	-7.622
C14B9.14	1.745	1.756	1.771	0	0	0	-5.735
F25H10.6	0	0	0	1.544	1.524	1.542	6.702
C02E7.16	3.3	3.3	3.3	0	0	0	-7.417
F32B5.12	1.337	1.224	1.684	0	0	0	-6.425
K09A11.9	0	0	0	2.699	2.694	2.63	6.384
Y42A5A.9	0.153	0.153	0.13	2.701	2.081	2.006	3.638
R13H4.10	1.56	1.45	1.964	0.487	0.407	0.438	-2.244
T24D3.3	2.1	1.444	2.558	0	0	0	-5.902
Y40B10A.10	1.644	1.511	1.544	114.45	116.694	113.469	6.067
C15F1.14	0.627	0.627	0.668	7.55	7.67	7.042	3.25
T07A5.8	2.455	2.626	2.257	0	0	0	-6.722
T12B3.8	1.422	1.342	1.444	0	0	0	-6.187
Y22F5A.10	2.322	2.445	2.208	0	0	0	-6.722
C34G6.9	4.887	4.234	4.045	0.756	0.725	0.738	-2.556
F26F12.17	2.668	3.831	2.889	0	0	0	-7.177
C17H12.33	0.447	0.447	0.686	0	0	0	-5.735
F52H2.12	1.054	1.044	1.01	0	0	0	-5.547
T19A5.10	1.734	1.711	1.719	0	0	0	-6.425
T05A12.7	1.033	1.223	1.01	0	0	0	-5.547
Y51A2D.33	0	0	0	1.211	1.241	1.092	5.716
R186.12	0	0	0	3.312	3.398	3.31	5.974
T10H4.19	1.164	1.134	1.163	0	0	0	-5.735
E01G4.8	6.112	6.561	6.093	0	0	0	-6.722
F08G5.15	3.342	3.332	3.173	0	0	0	-6.722
H34C03.20	10.228	8.998	8.427	0	0	0	-7.471

T06H11.15	0.912	0.902	0.993	0	0	0	-6.187
F49E10.16	1.445	1.766	1.041	0	0	0	-5.547
K07C10.7	0	0	0	1.32	1.382	1.302	5.974
F52H3.9	3.554	3.167	3.121	0	0	0	-6.052
D2023.16	0.327	0.372	0.373	5.667	5.681	5.105	3.47
R166.9	1.034	1.221	1.089	0.233	0.209	0.243	-2.244
Y71D11A.8	2.723	2.722	2.781	0	0	0	-6.425
F11D5.20	0.124	0.124	0.138	1.119	1.239	1.887	3.47
T01B4.6	0	0	0	2.55	2.5	2.504	6.194
W01A11.12	0.735	0.446	0.758	0	0	0	-5.735
DL2.1	0	0	0	2.533	2.333	2.225	6.384
C04G6.14	122.822	121.228	127.802	15.106	15.116	15.158	-3.189
F45H7.11	0.488	0.482	0.454	0	0	0	-6.311
W07E11.5	9.097	9.113	9.65	0.425	0.445	0.446	-4.424
C14F11.22	15.321	14.456	14.056	3.231	3.451	3.058	-2.308
F17C8.11	0.216	0.206	0.264	1.458	1.479	1.808	2.569
H36N01.2	1.162	1.326	1.644	0	0	0	-6.311
W06B11.9	0	0	0	1.137	1.373	1.103	5.974
H36L18.3	1.779	1.668	1.349	0	0	0	-5.902
C56C10.14	4.411	4.502	4.76	0.96	0.93	0.904	-2.483
Y54G2A.64	0	0	0	4.11	4.101	4.15	7.622
AC3.15	0.445	0.544	0.518	2.623	2.622	2.657	2.201
F32A11.9	67.452	67.288	65.233	6.407	6.437	6.472	-3.445
F45D3.9	1.812	1.801	1.875	9.634	9.656	9.616	2.23
C04F6.8	1.331	1.322	1.084	0	0	0	-5.547
R13H8.3	1.778	1.556	1.142	0	0	0	-5.902
ZK550.42	1.033	1.443	1.049	0	0	0	-5.547
T13H2.8	1.22	1.044	1.032	0	0	0	-6.187
F11D5.21	0.812	0.805	0.855	0	0	0	-6.187
F31B9.5	1.035	1.233	1.036	0	0	0	-5.735
K12D12.9	3.611	3.112	2.971	0	0	0	-7.177
F42E11.6	5.443	5.851	5.1	1.542	1.322	1.162	-2.221
F13D11.15	2.776	2.879	2.364	0	0	0	-6.89
F17H10.7	0	0	0	1.778	1.718	1.771	6.384
F25B4.14	1.005	1.443	1.215	0	0	0	-5.902
F09E5.20	3.455	3.278	3.283	0	0	0	-6.052
F47F6.11	9.121	9.788	9.139	0.453	0.436	0.434	-4.384
K11D2.6	1.444	1.766	1.561	0	0	0	-6.052
F26A10.20	6.227	6.337	6.69	1.5	1.413	1.466	-2.283
C48E7.14	1.522	1.555	1.529	0	0	0	-6.187
F21C10.16	1.228	1.023	1.777	0.466	0.488	0.434	-2.114
K11E4.10	2.789	2.893	2.769	0	0	0	-6.722
W04G3.14	2.332	2.433	2.221	0	0	0	-5.547

B0350.73	0.556	0.332	0.892	0	0	0	-5.902
Y48B6A.20	0	0	0	1.232	1.225	1.205	5.716
F13H6.23	0.668	0.509	0.85	0	0	0	-6.052
F29F11.13	2.545	2.556	2.89	0	0	0	-7.302
C13B9.5	0.556	0.622	0.644	0	0	0	-5.547
B0495.20	3.773	3.897	3.173	0	0	0	-7.302
F42D1.11	0.129	0.15	0.139	6.789	6.349	6.889	5.316
T19F4.7	0.657	0.337	0.692	0	0	0	-5.735
C54D2.12	0.967	0.883	0.959	0	0	0	-5.547
K08E4.11	0.124	0.123	0.127	1.567	1.877	1.736	3.47
Y69A2AR.41	2.327	2.333	2.72	0	0	0	-6.311
K01A11.7	1.037	1.036	1.08	0	0	0	-5.735
T24H7.7	1.733	1.778	1.703	0	0	0	-6.187
C55B6.6	0.633	0.655	0.619	3.567	3.457	3.704	2.421
D1007.22	1.122	1.446	1.174	0	0	0	-5.902
F28F5.13	2.334	2.444	2.019	0	0	0	-6.531
K02E10.9	0	0	0	2.673	2.697	2.675	5.974
T27B1.6	0	0	0	1.393	1.325	1.377	6.838
R03A10.11	0.149	0.159	0.186	1.423	1.413	1.429	2.737
F41C6.13	1.443	1.664	1.092	0	0	0	-5.547
K04G2.13	1.223	1.332	1.164	0	0	0	-6.187
ZK867.16	2.564	2.335	2.194	0	0	0	-7.361
F53G12.15	6.335	6.456	6.9	1.675	1.585	1.529	-2.273
B0414.12	3.443	3.377	3.324	0	0	0	-6.187
T08G11.9	2.322	2.606	2.666	0	0	0	-7.111
C15B12.12	0.448	0.811	0.831	0	0	0	-6.187
F29G9.13	7.112	7.664	7.43	1.878	1.678	1.852	-2.104
K09G1.5	0.444	0.777	0.82	0	0	0	-5.902
T19E7.26	6.788	6.411	6.107	0	0	0	-6.808
Y51B9A.12	0.747	0.757	0.768	5.132	5.252	5.249	2.622
C41G11.15	0.085	0.084	0.085	1.381	1.371	1.313	3.638
F47A4.6	0	0	0	1.342	1.135	1.171	5.716
C16B8.8	0.32	0.15	0.199	2.022	2.012	2.045	3.149
C18H9.10	1.812	1.229	1.858	0	0	0	-6.311
K10H10.14	0	0	0	1.014	1.037	1.031	6.552
Y42A5A.10	0	0	0	4.025	4.046	4.012	7.544
W01C8.13	1.124	1.145	1.163	0	0	0	-5.735
R07H5.12	0.901	0.912	0.961	0	0	0	-6.187
F55A12.15	4.245	4.233	3.218	0	0	0	-7.302
D1053.6	1.332	1.1	1.251	0	0	0	-5.902
W08D2.11	0.363	0.643	0.635	8.442	8.562	8.245	3.564
T05E11.10	0.778	0.888	0.806	3.576	3.676	3.616	2.03
C56G3.4	1.347	1.337	1.692	0	0	0	-6.311

Y14H12A.3	5.544	5.211	5.389	0	0	0	-6.63
C50C3.15	0	0	0	0.742	0.72	0.733	6.384
F08G5.16	1.443	1.332	1.851	0	0	0	-6.531
F26A1.17	0.943	0.439	0.901	0	0	0	-5.735
C33D12.13	1.143	1.155	1.128	0	0	0	-5.735
D1054.22	0.435	0.546	0.889	0	0	0	-5.547
C35C5.19	0.318	0.328	0.383	2.246	2.245	2.621	2.598
H14N18.6	1.233	1.442	1.207	6.455	6.322	6.188	2.224
C40H5.15	0.789	0.889	0.914	0.093	0.095	0.092	-3.309
T19H12.13	5.551	5.991	5.077	1.544	1.677	1.302	-2.061
C28A5.9	1.99	1.34	1.976	0	0	0	-6.187
F26E4.18	23.331	22.133	27.132	3.625	3.783	3.255	-3.159
F10F2.13	0.139	0.129	0.191	1.57	1.356	1.963	3.149
F43G9.18	14.366	13.364	13.379	1.03	1.024	1.1	-3.695
F58F6.9	1.223	1.773	1.325	0	0	0	-5.735
F35B12.14	0	0	0	2.001	2.066	2.02	6.552
ZK287.15	0.555	0.554	0.614	0	0	0	-5.902
Y77E11A.20	4.22	4.12	4.028	0	0	0	-6.187
Y15E3A.9	1.669	1.669	1.269	0	0	0	-5.902
H02I12.11	1.322	1.766	1.433	0	0	0	-5.547
Y73B6BL.281	2.983	4.45	2.825	0	0	0	-7.471
C34F11.15	0	0	0	0.568	0.348	0.775	5.716
Y37F4.13	6.665	6.365	6.454	36.333	32.344	35.304	2.328
F45H7.13	0	0	0	0.603	0.626	0.601	6.384
C07G1.13	5.986	7.766	5.157	0	0	0	-7.417
Y54G2A.66	0.557	0.6	0.579	0	0	0	-5.902
Y71H2AL.3	1.227	1.612	1.692	0	0	0	-5.735
T12G3.9	0	0	0	3.754	3.795	3.79	7.462
Y71A12B.29	2.099	2.122	2.369	0.433	0.443	0.434	-2.526
R02D5.18	1.323	1.448	1.823	0	0	0	-5.902
F35B12.15	0.943	0.908	0.934	0	0	0	-5.735
Y48B6A.21	0.512	0.599	0.515	4.444	4.564	4.398	2.933
F01G12.15	2.224	2.512	2.596	0	0	0	-6.311
F14H8.10	2.222	1.544	1.823	0	0	0	-5.902
K02D7.8	0.221	0.231	0.214	2.239	2.359	2.925	3.47
C24H11.11	0	0	0	2.001	2.056	2.02	6.552
T24B8.19	0	0	0	1.79	1.79	1.726	6.194
K07C5.15	0.878	0.89	0.54	0	0	0	-5.547
ZC412.12	0	0	0	6.4	6.003	6.34	7.283
T24A11.7	0	0	0	1.741	1.409	1.747	6.384
W07G1.16	0	0	0	0.927	0.927	0.981	5.974
F15A8.11	0	0	0	1.233	1.113	1.302	5.716
K08A2.15	2.779	2.244	2.259	0	0	0	-5.547

C07A9.16	26.556	26.613	29.618	5.315	5.235	5.352	-2.58
T14G10.10	2.788	2.666	2.364	0	0	0	-6.89
T27F7.6	2.776	2.655	2.158	0	0	0	-6.808
T19A5.11	0	0	0	1.239	1.289	1.205	6.194
F57F5.7	1.511	1.5	1.523	0	0	0	-6.311
C17G10.12	2.345	2.044	2.461	0	0	0	-6.968
ZC518.7	0	0	0	2.197	2.17	2.17	6.552
R06C1.12	2.222	2.334	2.347	0	0	0	-5.902
K06C4.23	0	0	0	0.603	0.533	0.7	6.384
F12F3.8	1.235	1.454	1.516	0.156	0.126	0.162	-3.222
F35H8.11	3.349	4.167	3.195	0	0	0	-7.361
C09B9.82	1.712	1.758	1.777	8.347	8.675	8.534	2.141
D2024.17	3.112	3.092	3.173	0.789	0.735	0.775	-2.128
M79.13	1.544	1.623	1.38	0	0	0	-6.052
T02C12.8	9.674	11.456	9.359	0	0	0	-7.574
F49B2.8	0	0	0	0.964	0.964	0.908	6.194
Y48G8AR.6	1.552	1.452	1.163	0	0	0	-5.735
Y17G7A.4	9.952	7.679	8.96	0	0	0	-7.715
C52G5.8	1.563	1.367	1.348	0	0	0	-5.735
Y59A8B.32	2.113	2.1	2.813	0.354	0.367	0.37	-2.925
C08D8.6	0.899	0.879	0.886	7.123	7.788	7.19	2.859
F31B12.7	1.444	1.432	1.206	0	0	0	-6.531
F28F9.13	2.92	2.95	2.904	32.834	23.346	19.858	2.648
ZK270.3	0.911	0.955	0.943	0	0	0	-6.187
T27D12.10	1.457	1.634	1.67	9.341	9.123	9.136	2.302
R11E3.15	1.34	1.43	1.015	0	0	0	-5.735
K08A8.22	1.322	1.452	1.24	0	0	0	-6.531
C31H2.13	1.312	1.322	1.385	0	0	0	-6.311
C38C5.3	1.333	1.379	1.396	0	0	0	-6.187
C02B4.13	0	0	0	1.655	1.853	1.847	6.384
ZC123.8	0	0	0	0.268	0.638	0.675	5.716
C55H1.4	3.244	3.542	3.227	0	0	0	-6.052
C48D5.8	7.556	7.766	7.1	1.122	1.176	1.103	-2.743
C05H8.4	1.618	1.622	1.68	0	0	0	-6.311
K03H4.6	1.332	1.122	1.198	0	0	0	-5.902
F11A1.13	3.716	4.536	5.712	0	0	0	-7.999
F38H4.15	2.044	2.3	2.281	0.542	0.462	0.424	-2.52
Y54G9A.16	11.218	10.567	10.618	0	0	0	-7.574
C16B8.9	1.132	1.311	1.12	0	0	0	-5.547
T06F4.16	1.738	1.756	1.785	0.309	0.356	0.339	-2.456
R08B4.13	0.967	0.912	0.926	0	0	0	-5.547
F29G9.14	16.923	17.43	11.424	0	0	0	-7.759
F14F11.5	0	0	0	2.862	2.166	2.872	5.974

C13D9.13	0.318	0.338	0.383	2.146	2.256	2.621	2.598
T09B4.17	1.447	1.422	1.485	0	0	0	-6.187
ZK180.15	0	0	0	1.336	1.436	1.356	5.716
C34E7.8	0	0	0	1.136	1.216	1.162	5.716
C03A3.6	0.751	0.703	0.77	0	0	0	-5.547
ZK180.16	0.116	0.113	0.106	6.341	6.651	6.147	5.544
W06H8.14	0	0	0	3.437	3.372	3.344	7.283
B0334.19	3.9	3.234	3.935	0.443	0.453	0.434	-3.235
B0350.76	5.447	5.567	5.745	1.124	1.145	1.122	-2.443
F38B6.18	1.341	1.114	1.112	0	0	0	-5.735
C08G9.9	3.679	3.887	3.13	0	0	0	-7.302
C24A1.6	1.502	1.236	1.587	0	0	0	-6.311
ZK970.11	2.345	2.456	2.552	0	0	0	-6.425
F35G2.16	4.026	4.132	4.533	0	0	0	-7.844
Y37E3.29	2.656	2.767	2.573	0.249	0.229	0.293	-3.188
Y46G5A.45	4.443	4.745	4.291	0	0	0	-6.722
F22B3.18	5.346	5.656	5.921	0.749	0.719	0.794	-2.984
F46F3.16	1.344	1.334	1.166	0	0	0	-5.902
H39E23.4	1.224	1.114	1.705	0	0	0	-6.311
ZK973.23	2.222	1.452	1.382	0	0	0	-5.902
R03E9.9	1.113	1.1	1.861	0.452	0.566	0.245	-2.925
C14F5.10	0	0	0	2.681	2.607	2.698	7.375
Y38F2AL.10	6.456	7.157	5.193	0	0	0	-7.471
H03A11.11	1.225	1.224	1.533	0	0	0	-6.311
F11D5.27	1.533	1.234	1.574	0	0	0	-7.111
K10D6.17	2.099	3.113	2.869	0	0	0	-7.177
C05B10.18	1.812	1.733	1.719	0	0	0	-6.425
Y51H7C.32	4.226	4.115	4.57	0	0	0	-6.311
T07F10.11	2.445	2.421	2.418	0.157	0.167	0.172	-3.802
C34F11.16	1.668	1.888	1.574	0	0	0	-6.052
F38A3.5	1.556	1.765	1.331	0	0	0	-6.052
F48D6.5	0	0	0	1.138	1.279	1.179	5.716
F45D3.12	2.554	2.113	2.83	0	0	0	-7.177
H01A20.4	0	0	0	0.991	0.914	0.901	6.384
Y37B11A.5	0	0	0	2.312	2.124	2.013	5.974
F09C3.9	26.117	16.66	18.659	0	0	0	-8.106
Y23H5A.11	0.834	0.844	0.889	0	0	0	-5.547
T10H10.6	0.813	0.821	0.828	6.337	6.234	6.368	2.797
F44D12.18	2.222	2.445	2.15	0	0	0	-6.531
F40B5.7	8.547	8.455	8.068	0	0	0	-7.361
C17C3.23	0	0	0	4.429	4.495	4.413	7.375
C25F9.17	1.344	1.163	1.166	0	0	0	-5.902
R13H8.4	0.765	0.865	0.645	4.296	4.196	4.965	2.737

D2045.10	0	0	0	3.911	3.914	3.921	6.702
F54A5.5	0.235	0.245	0.254	2.639	2.564	2.387	3.024
Y69E1A.13	2.322	2.755	2.187	0	0	0	-6.808
F26D12.100	3.945	3.374	3.831	0	0	0	-7.759
Y73B6BL.283	1.345	1.336	1.32	0	0	0	-5.547
Y43F8B.26	3.223	3.013	3.3	0.55	0.436	0.434	-2.982
F42H10.15	3.503	4.711	3.713	0	0	0	-7.999
T04F3.11	1.789	1.999	1.078	0	0	0	-5.902
F46F3.18	1.022	1.455	1.251	0	0	0	-5.902
Y70C5A.5	0	0	0	2.329	2.295	2.297	5.974
Y116A8C.466	1.225	1.113	1.523	0	0	0	-6.311
Y48C3A.23	0.359	0.349	0.389	3.567	3.625	3.653	3.024
Y42H9B.9	1.335	1.123	1.723	0.113	0.322	0.31	-2.533
B0001.12	0.854	0.737	0.889	0	0	0	-5.547
F25F2.5	13.033	16.332	15.009	1.726	1.716	1.759	-3.198
K10D6.20	0	0	0	1.947	1.925	1.982	6.963
ZK662.8	0.543	0.245	0.228	0	0	0	-5.547
R173.11	1.345	1.544	1.36	0	0	0	-5.735
T19A5.12	6.564	6.766	6.449	0.63	0.65	0.7	-3.288
C53C9.5	0.745	0.754	0.742	5.543	5.522	5.495	2.713
C06E7.93	6.123	6.231	6.146	0.517	0.527	0.571	-3.518
ZK792.10	1.328	1.563	1.278	6.228	6.348	6.771	2.273
Y39D8B.5	1.563	1.873	1.269	0	0	0	-6.052
C30D11.4	1.32	1.34	1.45	5.232	5.245	5.207	2.222
T02C5.7	1.933	0.769	0.901	0	0	0	-5.547
C01C4.6	1.788	1.556	1.515	0	0	0	-5.547
C36E8.10	4.444	4.644	4.433	1.032	1.44	1.033	-2.198
C01B10.47	65.338	65.335	65.754	18.444	16.455	17.408	-2.03
W01A11.15	1.655	1.787	1.913	0.176	0.156	0.156	-3.611
F17A9.7	20.13	20.113	20.981	5.133	5.255	5.2	-2.126
C24A8.12	0	0	0	1.038	1.028	1.085	5.716
C02G6.5	6.987	7.423	6.474	0	0	0	-8.385
B0457.15	0.233	0.456	0.892	0	0	0	-5.902
Y57G11C.1140	0.929	0.919	0.993	6.34	6.266	6.016	2.468
B0457.16	5.532	5.027	5.427	0	0	0	-8.642
F46E10.15	0.952	0.922	0.926	0	0	0	-5.547
F32B4.10	3.221	3.022	3.046	0	0	0	-6.311
T23E1.7	2.453	2.778	2.031	0	0	0	-6.722
C18D1.19	3.033	2.786	2.066	0	0	0	-7.523
H24G06.4	3.789	3.222	3.06	0	0	0	-5.902
T07D1.11	0.856	0.789	0.889	0	0	0	-5.547
F09C8.5	1.224	1.224	1.565	0	0	0	-6.311
C52G5.10	0.801	0.812	0.888	0	0	0	-5.902

C10F3.15	0.91	0.678	0.907	0	0	0	-5.547
K08A8.26	0.823	0.998	0.874	0	0	0	-5.902
C39H7.70	2.779	2.877	2.556	0	0	0	-7.041
F16B3.4	2.335	2.444	2.992	0	0	0	-6.187
F46F3.20	1.545	1.668	1.575	0	0	0	-6.187
T19H12.14	0.891	0.833	0.894	0	0	0	-5.547
C10C6.12	1.332	1.332	1.093	0	0	0	-6.311
B0222.14	1.779	1.501	1.587	0	0	0	-6.187
Y4C6B.100	65.339	64.229	64.862	13.345	16.124	15.839	-2.148
F31C3.16	0.123	0.123	0.127	2.449	2.119	2.939	4.312
F56E3.20	0.335	0.567	0.627	0	0	0	-5.735
Y67D8C.19	1.344	1.782	1.142	0	0	0	-5.902
K08B12.13	49.344	48.094	48.361	7.844	7.348	7.811	-2.742
C10G11.15	1.881	2.922	2.874	0	0	0	-7.715
F22B3.19	0.126	0.143	0.13	4.454	4.874	4.428	4.782
C14F11.30	0.158	0.168	0.18	2.452	2.233	2.159	3.369
K09A11.10	2.277	2.439	2.221	0	0	0	-6.531
Y17G7B.24	1.12	0.901	0.966	0	0	0	-5.547
C25F6.26	3.224	3.554	3.835	0.843	0.878	0.819	-2.326
C05E7.6	0	0	0	4.953	4.958	4.913	6.384
K02A4.12	0	0	0	2.444	2.439	2.484	6.702
F16F9.17	0	0	0	2.252	2.273	2.245	5.974
M01A8.3	5.464	6.433	3.765	0	0	0	-7.844
T14F9.19	0.622	0.611	0.602	0	0	0	-6.187
ZK381.57	0.955	0.933	0.901	0	0	0	-5.547
M01A8.4	25.224	23.444	25.432	1.263	1.463	1.627	-4.063
Y51B9A.13	2.335	2.844	2.842	0	0	0	-6.052
F13D11.19	0	0	0	0.498	0.461	0.477	6.194
C04D8.4	4.818	4.634	4.559	0	0	0	-7.802
Y73B6BL.286	2.334	3.222	2.221	0	0	0	-5.547
C34F11.18	4.1	4.127	4.657	0.437	0.447	0.468	-3.367
M04B2.12	3.988	3.688	3.415	0.344	0.315	0.354	-3.324
F18A11.12	1.335	1.545	1.496	0	0	0	-6.187
C07H6.18	1.336	1.222	1.62	0	0	0	-6.311
K02B2.38	2.448	2.348	2.766	0.228	0.238	0.278	-3.309
C53B7.12	0.665	0.775	0.48	0	0	0	-6.187
C18F3.11	1.433	1.522	1.304	0	0	0	-6.63
F16F9.19	2.333	1.022	1.288	0	0	0	-5.902
C18F3.12	4.934	3.23	3.912	0	0	0	-7.622
ZC308.6	2.433	2.677	2.202	0	0	0	-6.808
R13H8.5	1.432	1.542	1.238	0	0	0	-5.735
Y38F2AL.11	2.324	2.033	2.45	0	0	0	-7.177
B0212.9	0	0	0	2.303	2.234	2.034	5.716

ZC477.19	1.434	1.622	1.904	0	0	0	-6.63
ZK945.15	1.328	1.345	1.785	0	0	0	-5.902
M04B2.13	0	0	0	1.458	1.483	1.436	6.963
C02C6.11	1.32	1.533	1.503	0	0	0	-5.902
W09C2.12	0.292	0.22	0.297	3.305	3.233	3.051	3.149
F52H2.13	0	0	0	2.089	2.095	2.084	7.078
F11D5.31	1.178	1.024	0.79	0	0	0	-5.902
C14F5.11	0	0	0	1.685	1.649	1.697	6.838
C37A2.9	6.113	5.333	6.347	0.944	0.923	0.923	-2.866
M176.14	1.733	1.567	1.711	0	0	0	-5.735
F08G5.21	0	0	0	1.271	1.171	1.071	5.716
C24A8.13	5.345	5.675	5.532	0.444	0.424	0.44	-3.705
W01A11.18	0.611	0.61	0.685	0	0	0	-6.052
Y15E3A.13	0	0	0	2.193	2.133	2.188	6.384
B0457.17	0.679	0.833	0.88	0	0	0	-5.735
ZK669.9	2.311	2.322	2.347	0	0	0	-5.902
C30D11.5	2.128	2.438	2.821	0	0	0	-5.735
F26D11.19	0.569	0.778	0.92	0	0	0	-6.052
Y54G2A.70	0.623	0.412	0.657	3.523	3.723	3.367	2.187
C26E6.15	0	0	0	3.44	3.964	3.684	5.974
F57A10.9	1.883	1.081	1.08	0	0	0	-5.735
T24B8.21	1.133	1.222	1.166	0	0	0	-5.902
T21C12.11	1.556	1.799	1.425	0	0	0	-6.531
W06B11.11	1.623	1.227	1.65	0	0	0	-6.425
F15A2.14	0.433	0.412	0.431	0	0	0	-5.547
C30B5.16	1.224	1.533	1.554	0	0	0	-6.311
T09B4.19	0.458	0.656	0.779	3.12	3.24	3.994	2.224
C29A12.21	0	0	0	0.73	0.74	0.75	5.716
T06F4.19	0.235	0.234	0.238	3.223	3.234	3.255	3.561
C48B6.13	3.124	3.099	3.414	0.422	0.433	0.449	-2.982
C33D12.15	1.67	1.777	1.19	0	0	0	-5.735
C04F1.5	1.233	1.211	1.269	0	0	0	-6.052
F54D5.22	0	0	0	1.382	1.32	1.302	5.974
ZC250.11	0.335	0.604	0.629	0	0	0	-5.547
F46F3.23	0.633	0.546	0.629	0	0	0	-5.547
F10D11.8	0.236	0.246	0.261	1.346	1.456	1.635	2.474
C37H5.16	0.941	0.931	0.91	10.336	12.776	11.606	3.542
K08A8.28	1.788	1.911	1.877	0	0	0	-6.531
C04A2.13	0.434	0.456	0.598	0	0	0	-5.547
ZK370.11	1.444	1.554	1.437	0	0	0	-6.722
C27C12.10	0.409	0.543	0.481	0	0	0	-5.547
K11C4.13	2.555	3.444	2.468	0	0	0	-7.523
Y38F1A.13	1.445	1.043	1.009	0	0	0	-6.722

C13C4.12	0.335	0.375	0.355	2.56	2.57	2.021	2.337
Y67A6A.3	65.328	65.228	65.28	500.112	511.222	507.245	2.842
C50C3.18	1.663	1.893	1.043	0	0	0	-5.735
C04A2.14	0.885	0.999	0.715	0	0	0	-5.735
W09C2.13	3.909	3.233	3.808	0	0	0	-7.041
M117.13	1.457	1.877	1.445	0	0	0	-6.808
C14H10.5	3.229	3.129	3.936	0.326	0.324	0.364	-3.489
W10C8.16	1.456	1.554	1.496	0	0	0	-6.187
T05B11.12	6.335	6.226	6.606	1.225	1.533	1.55	-2.191
K04A8.17	0	0	0	1.32	1.3	1.302	5.974
F59B10.10	1.222	1.222	1.215	0	0	0	-5.902
T27B1.8	0.216	0.276	0.257	1.348	1.256	1.759	2.569
F29G9.17	0.878	0.69	0.889	0	0	0	-5.547
T09A5.21	0	0	0	3.89	3.805	3.869	7.462
F48B9.12	1.443	1.663	1.331	7.225	7.445	7.511	2.363
K07C6.17	0	0	0	7.49	7.497	7.439	7.375
K08A8.30	1.234	1.434	1.956	0	0	0	-6.63
F47F6.14	3.334	3.877	3.173	0	0	0	-6.89
R05G9.4	1.778	1.067	1.088	0	0	0	-5.735
C34E11.19	2.453	2.46	2.739	0	0	0	-7.111
C11D2.101	2.443	2.553	2.277	0	0	0	-6.187
Y67D2.10	4.344	5.566	5.331	0	0	0	-6.531
T27D1.4	1.233	1.223	1.269	0	0	0	-6.052
Y113G7A.21	2.101	1.234	1.142	0	0	0	-5.902
M60.14	0.87	0.81	0.9	6.456	6.678	6.407	2.686
B0457.19	5.135	6.455	5.346	0	0	0	-9.605
W04G3.16	1.349	1.119	1.904	0	0	0	-5.902
F46E10.17	0.619	0.419	0.194	1.249	1.149	1.494	2.737
C54G7.16	2.231	2.331	2.308	17.326	18.223	17.259	2.776
C40H1.13	2.456	2.115	2.497	0	0	0	-5.735
BE10.7	2.554	2.665	2.031	0	0	0	-6.722
B0350.84	0.678	0.666	0.565	0	0	0	-5.547
R03H4.11	5.879	5.989	5.853	0.104	0.132	0.11	-5.718
C27A12.12	0	0	0	1.149	1.139	1.179	5.716
Y15E3A.15	0.224	0.118	0.777	0	0	0	-6.311
K02E2.16	1.555	1.668	1.015	0	0	0	-5.735
T01A4.6	0.433	0.444	0.402	2.321	2.445	2.063	2.187
M176.15	1.978	2.292	1.939	0	0	0	-7.177
F28D9.5	1.557	1.448	1.777	0	0	0	-6.531
F28D1.22	0.633	0.779	0.6	0	0	0	-5.547
Y105C5B.1417	1.345	1.303	1.325	0	0	0	-5.735
Y53C10A.23	2.556	2.054	2.045	0	0	0	-6.722
C05A9.11	2.513	2.552	2.821	0.743	0.721	0.723	-2.044

C34H3.23	12.335	14.653	11.337	1.438	1.488	1.479	-3.041
T10B9.13	5.116	5.136	5.553	1.323	1.566	1.356	-2.131
Y69H2.26	0.812	0.456	0.894	0	0	0	-5.547
Y23H5B.13	0	0	0	2.976	2.759	2.56	6.194
F10C1.20	1.133	1.771	1.111	0	0	0	-5.547
E01H11.6	2.554	2.655	2.116	0	0	0	-6.63
K11D9.8	0.568	0.667	0.745	0.095	0.098	0.091	-3.031
C30C11.13	1.333	1.333	1.35	0	0	0	-6.052
F47E1.16	0	0	0	1.76	1.76	1.736	6.384
C06A8.17	0	0	0	1.791	1.912	1.979	6.552
F26A10.27	1.123	1.332	1.174	0	0	0	-5.902
DY3.12	2.565	2.568	2.608	0.423	0.444	0.446	-2.606
F25F6.4	2.615	2.333	2.391	0	0	0	-7.417
ZK829.13	0	0	0	1.227	1.272	1.332	6.194
F07C3.16	0	0	0	0.361	0.561	0.661	5.716
T09F5.20	0.523	0.226	0.597	0	0	0	-6.311
F44E5.15	0.565	0.678	0.421	0	0	0	-7.361
T24H7.8	1.022	1.124	1.008	0	0	0	-6.968
M04C3.5	0.234	0.323	0.267	0	0	0	-5.902
F59C6.18	0.035	0.045	0.047	0.611	0.621	0.648	3.47
C44C11.6	1.33	1.044	1.02	0	0	0	-5.902
C31H2.14	1.44	1.556	1.002	0	0	0	-6.531
ZK757.10	1.632	1.662	1.618	24.278	23.773	23.271	3.727
Y73F8A.1173	0.566	0.775	0.436	0	0	0	-5.735
T01B7.13	1.335	1.555	1.511	0.335	0.333	0.387	-2.044
T26E4.18	1.334	1.555	1.104	0	0	0	-5.735
Y52B11A.19	0.564	0.786	0.88	0	0	0	-6.531
C14A6.16	0.057	0.078	0.095	0.016	0.017	0.012	-3.031
F43G6.16	0.722	0.744	0.797	0	0	0	-5.902
W02G9.10	0.413	0.433	0.434	3.345	3.456	3.026	2.672
C33C12.12	0.612	0.557	0.693	0	0	0	-6.425
F17C11.22	0.063	0.065	0.069	0.544	0.445	0.469	2.598
F36D3.16	0.665	0.543	0.416	0	0	0	-7.302
F34H10.9	0.545	0.389	0.669	0.082	0.083	0.088	-2.982
C49H3.16	1.088	1.113	1.75	0.13	0.103	0.103	-4.135
K12G11.14	1.331	1.541	1.106	2.899	4.113	5.807	2.265
H21P03.10	0.812	0.822	0.897	0	0	0	-6.425
D1046.16	1.689	1.999	1.547	0	0	0	-7.574
C01F6.15	0	0	0	2.847	2.467	2.589	6.194
C03D6.9	1.023	1.223	1.086	0	0	0	-6.425
C04F12.17	0.645	0.554	0.694	0	0	0	-5.902
C12C8.8	2.923	3.238	2.79	0	0	0	-6.808
C18D1.21	2.234	2.114	2.364	0.424	0.445	0.425	-2.533

C34E11.20	0	0	0	5.899	5.991	5.169	8.126
C45E1.10	1.133	1.131	1.114	0	0	0	-6.187
C54G4.16	0.967	0.956	0.952	0	0	0	-5.735
F12F6.20	0	0	0	0.639	0.639	0.839	5.716
F16A11.13	8.333	8.566	8.299	1.932	1.968	1.947	-2.191
F20D12.10	0	0	0	1.675	1.675	1.627	6.384
F21F12.3	1.455	1.335	1.985	0.357	0.338	0.377	-2.474
F27D4.11	1.489	1.565	1.465	0	0	0	-5.547
F55H12.9	0.334	0.324	0.344	5.71	5.61	5.097	3.707
M7.15	2.221	2.334	2.355	0	0	0	-6.311
R10A10.3	1.223	1.133	1.154	0	0	0	-6.311
R166.10	0	0	0	2.177	2.118	2.152	6.384
T05A6.20	1.995	1.115	1.549	0	0	0	-6.808
W06D12.9	0	0	0	1.384	1.336	1.308	6.384
Y17G7B.25	1.222	1.023	1.503	0.117	0.127	0.171	-3.13
Y44A6C.3	0	0	0	1.461	1.561	1.261	5.716
Y47G6A.36	7.712	10.735	9.76	0	0	0	-8.642
Y51H4A.939	4.238	4.103	4.59	0	0	0	-8.467
Y69E1A.15	0	0	0	2.566	2.437	2.466	5.716
Y73B6A.15	0.204	0.206	0.205	9.888	10.228	9.799	5.265
Y75B7AL.9	2.335	2.55	2.995	0	0	0	-6.531
Y79H2A.14	0	0	0	2.326	2.136	2.358	5.716
ZK185.9	0.733	0.712	0.708	0	0	0	-5.735
ZK270.5	0	0	0	1.245	1.45	1.752	6.194
ZK484.11	0	0	0	2.827	2.827	2.841	6.838
Y57G11A.226	151.223	133.673	153.271	7.205	7.305	7.052	-4.552
C25G4.16	0	0	0	1.59	1.898	1.726	6.194
LLC1.121	0.123	0.111	0.199	0	0	0	-6.052
K09F6.13	2.101	1.981	1.11	0	0	0	-8.205
K10H10.15	22.123	21.213	23.134	355.655	355.665	357.651	3.835
F59E12.15	0.458	0.667	0.533	0	0	0	-7.302
C49C8.8	0.565	0.578	0.505	0	0	0	-6.722
C54E4.12	11.234	12.981	12.079	1.33	1.34	1.4	-3.214
ZK185.10	0.222	0.201	0.231	0	0	0	-5.735
C01F6.16	1.221	1.133	1.127	0.223	0.221	0.275	-2.122
F28E10.16	0	0	0	0.104	0.14	0.174	5.974
Y69A2AR.48	1.343	1.459	1.293	0	0	0	-6.187
B0222.15	0	0	0	1.811	1.111	1.298	7.184
K11D5.2	2.122	2.223	2.306	0.504	0.534	0.509	-2.268
F31F7.9	42.227	43.733	44.748	13.112	10.123	11.163	-2.117
Y54G2A.77	0.234	0.255	0.215	0	0	0	-6.187
ZK250.13	0.556	0.431	0.221	0	0	0	-7.471
K08D10.15	0.091	0.092	0.091	0.622	0.612	0.62	2.569

H32C10.101	0	0	0	1.953	1.527	1.964	6.194
F54E2.8	23.035	22.33	25.046	5.786	5.796	5.641	-2.265
C54G6.8	1.552	1.443	1.002	0	0	0	-5.902
Y48G8AL.20	0.564	0.712	0.641	0.04	0.06	0.076	-3.139
F42G8.19	0.322	0.333	0.486	0	0	0	-6.531
M199.136	0	0	0	0.211	0.211	0.235	6.194
C06A12.19	0	0	0	0.347	0.473	0.373	5.716
B0348.10	0.346	0.444	0.367	0	0	0	-5.547

clec-143	0	0	0	0.065	0.063	0.061	5.731
C49A1.5	0	0	0	0.141	0.189	0.158	6.293
ptr-22	0	0	0	0.165	0.171	0.132	5.374
skn-1	0.403	0.425	0.436	6.144	6.114	6.343	3.128
T19H5.6	0	0	0	0.053	0.065	0.063	7.084
F31F7.1	0	0	0	0.198	0.199	0.153	6.394
C25F9.9	0	0	0	0.138	0.184	0.179	5.674
oac-43	0.004	0.003	0.001	0.042	0.035	0.074	3.989
ZK993.5	0.032	0.045	0.042	0.261	0.267	0.281	2.579
hlh-30	0	0	0	0.212	0.212	0.334	6.464
M04C9.2	0	0	0	0.192	0.182	0.163	5.996
lbp-7	0	0	0	0.147	0.132	0.108	6.438
T23E7.6	0.434	0.554	0.641	6.324	6.244	6.287	3.292
daf-16	0	0	0	0.267	0.211	0.329	6.502
Y73B6BL.37	2.464	2.774	2.759	18.317	17.512	16.692	2.472
B0454.8	0.014	0.015	0.016	0.154	0.135	0.153	3.169
F44E5.4	1.287	1.933	0.957	0	0	0	-6.712
C52D10.3	1.344	1.534	1.817	0	0	0	-6.435
nhr-17	3.316	3.178	2.336	0	0	0	-6.859
Y94H6A.10	0.343	0.212	0.125	0	0	0	-6.849
oac-14	0.054	0.046	0.047	0	0	0	-5.912
R11A5.3	0.333	0.424	0.136	0	0	0	-6.331
Y53G8B.2	0.043	0.024	0.035	0	0	0	-6.152
fbxb-106	0.047	0.035	0.051	0	0	0	-5.795
clec-223	1.879	1.435	1.146	0	0	0	-5.912
Y82E9BL.3	3.769	3.977	3.679	0.832	0.833	0.858	-2.217
clec-184	1.453	1.564	1.012	0	0	0	-5.755

Note: $P < 0.05$

Table S9. Effect of simulated microgravity on gene expressions

Gene	Expression (Folds)
clec-143	2.59 ± 0.33
clec-139	2.56 ± 0.36
F54B11.11	2.65 ± 0.39
fipr-1	2.75 ± 0.33
C49A1.5	2.59 ± 0.15
ptr-22	2.46 ± 0.18
skn-1	2.72 ± 0.27
sodh-2	2.83 ± 0.12
R09H10.2	2.72 ± 0.18
T19H5.6	2.73 ± 0.21
C33C12.11	3.19 ± 0.27
F31F7.1	3.11 ± 0.29
C17B7.5	3.44 ± 0.21
C25F9.9	3.41 ± 0.24
oac-43	3.17 ± 0.27
ZK993.5	3.18 ± 0.38
hlh-30	3.25 ± 0.24
M04C9.2	3.42 ± 0.23
lbp-7	3.58 ± 0.23
F42A8.1	3.53 ± 0.2
T23E7.6	3.66 ± 0.09
daf-16	3.64 ± 0.12
F49C12.10	3.9 ± 0.11
Y73B6BL.37	4.26 ± 0.15
F15E6.3	4.19 ± 0.09
B0454.8	6.13 ± 0.26
F44E5.4	-6.42 ± 0.12

F19B2.5	-5.98 ± 0.14
C52D10.3	-4.82 ± 0.16
nhr-17	-4.35 ± 0.23
Y94H6A.10	-4.38 ± 0.13
oac-14	-4.16 ± 0.23
aqp-1	-4.04 ± 0.07
R11A5.3	-3.49 ± 0.2
Y53G8B.2	-3.16 ± 0.3
R11F4.2	-3.16 ± 0.25
fbxb-106	-3.14 ± 0.28
C07G1.7	-3.17 ± 0.29
clec-223	-2.76 ± 0.37
fbxa-66	-2.81 ± 0.36
Y82E9BL.3	-2.76 ± 0.32
clec-184	-2.79 ± 0.14
ugt-18	2.69 ± 0.18

Table S10. Data on effect of intestinal RNA knockdown of *linc-50* on gene expressions

	daf-16	*skn-1*	*hlh-30*
Control	83.8 ± 7.9	88.6 ± 3.4	84.2 ± 6.6
Simulated microgravity	236.3 ± 8.3	258.4 ± 5.9	259.3 ± 7

	daf-16	*skn-1*	*hlh-30*
VP303(L4440)	73.2 ± 6.6	76.6 ± 9.6	86.8 ± 5.2
linc-50(RNAi)	279.2 ± 8.9	241.4 ± 7.7	276.8 ± 5.9

Table S11. Data on effect of RNAi knockdown of *linc-50* on expression of DAF-16::GFP or SKN-1::GFP in simulated microgravity treated nematodes

% Relative fluorescence intensity	control	L4440	*linc-50(RNAi)*
	100 ± 6	187 ± 7	314 ± 13
% Animals with DAF-16::GFP nucleus localization	control	L4440	*linc-50(RNAi)*
	8.7 ± 4.2	47 ± 3.6	87 ± 2.2
% Relative fluorescence intensity	control	L4440	*linc-50(RNAi)*
	100 ± 7	187 ± 9	314 ± 15
% Animals with DAF-16::GFP nucleus localization	control	L4440	*linc-50(RNAi)*
	5.6 ± 3.1	45 ± 4.3	82 ± 3.4

Table S12. Data on genetic interaction between *linc-50* and DAF-16, SKN-1, or HLH-30 in regulating the toxicity of simulated microgravity in inducing ROS production

	Control	Simulated microgravity
VP303(L4440)	95.6 ± 3.4	173.4 ± 5.3
linc-50(RNAi)	91.2 ± 1.6	94.8 ± 6.2
daf-16(RNAi)	95.8 ± 6.3	287.8 ± 4.1
skn-1(RNAi)	97.6 ± 4.2	288.2 ± 3.3
hlh-30(RNAi)	94 ± 4.3	278.2 ± 5.9
daf-16(RNAi);linc-50(RNAi)	95.4 ± 5.8	280.8 ± 2.5
linc-50(RNAi)skn-1(RNAi)	91.6 ± 5.7	286.2 ± 5.7
linc-50(RNAi)hlh-30(RNAi)	93 ± 7.3	280.6 ± 5.4

Table S13. Data on qRT-PCR analysis of efficiency for intestine-specific RNAi knockdown of *daf-16*, *skn-1*, or *hlh-30*

	VP303(L4440)	*daf-16(RNAi)*
daf-16	100 ± 2.1	18.9 ± 2.7
	VP303(L4440)	*skn-1(RNAi)*
skn-1	100 ± 1.7	17.8 ± 2.6
	VP303(L4440)	*hlh-30(RNAi)*
hlh-30	100 ± 2.6	19.5 ± 3.3

Table S14. Data on qRT-PCR analysis of efficiency for intestinal RNAi knockdown in *daf-16(RNAi);linc-50(RNAi)*, *daf-16(RNAi);skn-1(RNAi)*, *daf-16(RNAi);hlh-30(RNAi)*, *skn-1(RNAi)hlh-30(RNAi)*, *linc-50(RNAi)skn-1(RNAi)*, and *linc-50(RNAi)hlh-30(RNAi)* nematodes

daf-16 expression	VP303(L4440)	*daf-16(RNAi); linc-50(RNAi)*	*daf-16(RNAi); skn-1(RNAi)*	*daf-16(RNAi); hlh-30(RNAi)*
	100 ± 3.2	14.88 ± 6.4	11.23 ± 5.7	10.99 ± 4.3
skn-1 expression	VP303(L4440)	*daf-16(RNAi); skn-1(RNAi)*	*skn-1(RNAi); hlh-30(RNAi)*	*linc-50(RNAi); skn-1(RNAi)*
	100 ± 5.9	12.92 ± 3.8	11.09 ± 2.9	13.78 ± 3.6
hlh-30 expression	VP303(L4440)	*daf-16(RNAi); hlh-30(RNAi)*	*skn-1(RNAi); hlh-30(RNAi)*	*linc-50(RNAi); hlh-30(RNAi)*
	100 ± 1.9	9.67 ± 6.9	13.12 ± 6.3	9.21 ± 3.7
linc-50 expression	VP303(L4440)	*daf-16(RNAi); linc-50(RNAi)*	*linc-50(RNAi); skn-1(RNAi)*	*linc-50(RNAi); hlh-30(RNAi)*
	100 ± 7.1	15.23 ± 2	13.44 ± 3.1	10.88 ± 7.2

Table S15. Data on genetic interaction among DAF-16, SKN-1, and HLH-30 in regulating the toxicity of simulated microgravity in inducing ROS production

	Control	Simulated microgravity
VP303(L4440)	93.8 ± 4.9	173.8 ± 3.7
daf-16(RNAi)	85.8 ± 2.5	273.2 ± 5.8
skn-1(RNAi)	89.2 ± 3.6	265.8 ± 6.1
hlh-30(RNAi)	91.8 ± 5.1	274.2 ± 8.4
daf-16(RNAi);skn-1(RNAi)	92.1 ± 5.5	355.8 ± 4.8
daf-16(RNAi);hlh-30(RNAi)	87.6 ± 3.7	361.2 ± 8.3
skn-1(RNAi)hlh-30(RNAi)	96.6 ± 4.6	358.6 ± 4

Table S16. Data on effect of intestinal RNAi knockdown of *linc-50* on expressions of *daf-16*, *skn-1*, and *hlh-30* under the normal condition

	daf-16	*skn-1*	*hlh-30*
VP303(L444)	109 ± 6	106 ± 4	119 ± 6.5
linc-50(RNAi)	189 ± 3.3	109 ± 5.1	191 ± 4.4

Table S17. Primer information for qRT-PCR

Gene	Forward primer (5'-3')	Reverse primer (5'-3')
linc-2	ATTTTTTCACGTCACTAA	TATATTTTTTAAAGGGTG
linc-7	CTGTTTGTTGGTGACGGCG	GCCGGAAGTGCGTGATCATA
linc-8	GAATGGCTGTCCCTCTTGCT	ACAACTGCCAAAGGACGCTA
linc-9	TGTCGAGCAGCGCTG	CTTGGATCATTTTCGGAG
linc-13	CAGAATGGGAGTTCTTGAGC	AAAGTGTTTACTACGGTCGA
linc-14	TCCTCTTGTCTTCTCTGGCTT	GGTTGATTACTTGTTGGCAGTC
linc-18	TCCTCGTTGCAATCACGATTTT	CGATTTGTGTCCTCCCACCA
linc-28	CATTTCGTTTTGCGTGAC	GCTTCTCATGTTTATTGA
linc-32	CGATTCACACCGTAAATC	AGACATACAATATTTATT
linc-37	CGTCCTGTCAATGCAATGTG	TGGTGTGAACTCATTACTTGTG
linc-46	TTGACGCCAAAACCAATGCC	GCAACACTCGTTGACTCTCAC
linc-50	ACGTCGCCAGACATCTGATT	ATTGTGCTCCGTCTGAGCTT
linc-61	GCCGATTTACAGTCTGTGTGC	TCTGGTGCCTGTAGAGGTGA
linc-78	CAAATTCGCTTGGCAGAT	TCCGGATAACTAAATTTA
linc-84	TGGAGGAGCTCTTGTGGAGT	TGTTGCGCCAGTGAGGATTA
linc-107	AACGTGGTGATGGTCAAGTCA	GCAAAGAGAGGGAATGAGTGGT
linc-125	CTCGGATGTCACGTGTTCCA	GTAACTCCTCCCCAACGCAA
linc-138	TAATAGGCGTTGCCCTTCGG	TTCGTCTCTTCGACACGTCC
linc-139	GTCAGGGAATTGCGGGGTAA	CCCTATTGGTACGCGGTTCA
linc-150	CACCGGCACTTTTCAGCATC	CGGGAAGGTGAGAAAACCGA
tts-1	AAACTTGACCGGCTCAACCA	TATCAAAAACCCGTCGTCGC
daf-16	AGGTGTTACACGTGGCCAAT	TGGCTTCTTACGACAACGCT
skn-1	AGGCTCAACCTCAGAACATG	TACGAGTAGGCGGTCATTTC
hlh-30	ATCGCATCTTCCACCGATCC	GCCGCTGCTCGTCCTATAAT
tba-1	TCAACACTGCCATCGCCGCC	TCCAAGCGAGACCAGGCTTCAG
